# Epidemiology of *Trichomonas vaginalis* infection in the Middle East and North Africa: systematic review, meta-analyses, and meta-regressions

**DOI:** 10.1016/j.ebiom.2024.105250

**Published:** 2024-07-17

**Authors:** Manale Harfouche, Wafaa Sekkal Gherbi, Asalah Alareeki, Ahmed S. Alaama, Joumana G. Hermez, Alex Smolak, Laith J. Abu-Raddad

**Affiliations:** a Infectious Disease Epidemiology Group, Weill Cornell Medicine-Qatar, Cornell University, Qatar Foundation-Education City, Doha, Qatar; b World Health Organization Collaborating Centre for Disease Epidemiology Analytics on HIV/AIDS, Sexually Transmitted Infections, and Viral Hepatitis, Weill Cornell Medicine-Qatar, Cornell University, Qatar Foundation-Education City, Doha, Qatar; c Department of Communicable Diseases, HIV/Hepatitis/STIs Unit, World Health Organization Regional Office for the Eastern Mediterranean, Cairo, Egypt; d Department of Population Health Sciences, Weill Cornell Medicine, Cornell University, New York, NY, USA; e Department of Public Health, College of Health Sciences, QU Health, Qatar University, Doha, Qatar; f College of Health and Life Sciences, Hamad bin Khalifa University, Doha, Qatar

**Keywords:** *Trichomonas vaginalis*, Trichomoniasis, Sexually transmitted infection, Prevalence, Middle east and North Africa

## Abstract

**Background:**

Trichomoniasis, caused by the parasite *Trichomonas vaginalis* (TV), remains an underappreciated sexually transmitted infection (STI), primarily due to inadequate understanding of its epidemiology and public health implications. This study aimed to characterize TV epidemiology in the Middle East and North Africa (MENA).

**Methods:**

Systematic review and analysis of evidence sourced from international, regional, and national databases were conducted. Findings were reported following PRISMA guidelines. Random-effects meta-analyses and meta-regressions were performed to determine pooled mean prevalence, investigate associations with prevalence, and identify sources of between-study heterogeneity.

**Findings:**

The review identified 263 relevant publications, encompassing 462 TV prevalence measures. The pooled mean TV prevalence was estimated as follows: 4.7% (95% CI: 3.9–5.6%) in the general population of women, 17.2% (95% CI: 5.4–33.6%) among intermediate-risk populations, 10.3% (95% CI: 6.2–15.3%) among female sex workers, 13.9% (95% CI: 12.3–15.6%) among symptomatic women, 7.4% (95% CI: 1.9–15.5%) among infertility clinic attendees, 2.3% (95% CI: 0.1–6.3%) among women with miscarriages or ectopic pregnancies, and 1.6% (95% CI: 0.8–2.7%) among STI clinic attendees. Limited data were found for men. Multivariable meta-regressions explained >40% of the prevalence variation, unveiling a hierarchical prevalence pattern by population type, an inverse correlation with national income, and a prevalence decline at a rate of 1% per calendar year.

**Interpretation:**

Despite conservative sexual norms, MENA has a substantial TV prevalence, comparable to the global TV prevalence. The unexpectedly high prevalence of this curable infection may, in part, be attributed to limited access to and underutilization of STI screening and treatment services.

**Funding:**

This work was supported by the Qatar Research, Development, and Innovation Council [ARG01-0522-230273] and by the Biomedical Research Program at 10.13039/100019460Weill Cornell Medicine-Qatar.

**Research in context**

**Evidence before this study**

Trichomoniasis, caused by the parasite *Trichomonas vaginalis* (TV), remains an underestimated sexually transmitted infection (STI) due to inadequate understanding of its epidemiology, public health impact, and limited resource allocation. The World Health Organization (WHO) estimated 156.3 million new cases of TV infection in 2020, surpassing the individual counts of chlamydia, gonorrhea, and syphilis. TV infection is linked to various health issues, particularly affecting women, including conditions such as pelvic inflammatory disease, infertility, and perinatal morbidity. WHO's “Global Health Sector Strategy on STIs” targets a 50% reduction in TV incidence by 2030. A PubMed search, employing a broad search strategy (“*Trichomonas vaginalis*" [MeSH] AND “Review" [Publication Type]), with no language or year limitations, found no systematic reviews or meta-analyses on TV infection in the Middle East and North Africa (MENA) or other regions.

**Added value of this study**

This study applied rigorous methodologies to reveal considerable data on TV prevalence in MENA, enabling diverse analyses and establishing an understanding of the infection's epidemiology. TV prevalence in the general population of women reached 4.7%, surpassing expectations for a region known for its conservative views on sexual behavior. While TV prevalence showed an annual decline of 1%, this modest rate of decline falls short of the WHO’s goal to halve TV incidence by 2030. Symptomatic women experienced markedly high TV prevalence, underscoring the disproportionate disease burden among women compared to men. TV prevalence exhibited an inverse relationship with national income and followed a hierarchical pattern, with higher rates among high-risk groups such as female sex workers.

**Implications of all the available evidence**

TV prevalence in MENA closely resembles global rates, indicating an under-recognized disease burden with psychosocial and economic consequences. The unexpectedly elevated prevalence of this curable infection could, to some extent, be attributed to limited access to and underutilization of STI screening and treatment services. Action is necessary to curb TV transmission and address its associated disease burden in MENA, as the current response falls short of aligning with the WHO's Global Health Sector Strategy on STIs. Challenges like enduring STI stigma, political sensitivities, and socio-cultural barriers hinder progress in establishing an appropriate public health agenda and a supportive environment for sexual health. To effectively address this burden, specialized and culturally sensitive programs tailored towards addressing gender-specific needs must be developed.

## Introduction

Trichomoniasis, caused by the protozoan parasite *T. vaginalis* (TV), is a sexually transmitted infection (STI) that affects humans as the sole natural host.^1^ While there have been few documented cases of non-sexual transmission, sexual contact remains the predominant mode of transmission.^2^ Despite its high prevalence as an STI, TV infection is under-recognized and underappreciated due to inadequate understanding of its epidemiology and public health implications, coupled with insufficient resource allocation to address the gaps in evidence on a global scale.^1^^,^^3^^,^^4^

In 2020, the World Health Organization (WHO) estimated that there were 156.3 million new cases of trichomoniasis, surpassing the individual counts of other prominent STIs such as chlamydia, gonorrhea, and syphilis.^5^^,^^6^ The majority of TV infections are asymptomatic, making early detection challenging and perpetuating TV transmission within populations.^1^^,^^2^^,^^7^

TV infection has been linked over the last three decades to an increasing spectrum of clinical manifestations and adverse reproductive health outcomes, with a pronounced impact on women.^1^, ^2^, ^3^^,^^7^, ^8^, ^9^, ^10^, ^11^, ^12^ Symptomatic presentations encompass vaginal discharge, itching, discomfort, dyspareunia, dysuria, pelvic inflammatory disease, cervical neoplasia, and infertility.^1^, ^2^, ^3^^,^^7^, ^8^, ^9^, ^10^, ^11^, ^12^, ^13^ Pregnant women with TV infection face an increased risk of preterm birth and low birth weight infants.^1^^,^^8^^,^^11^^,^^14^ In men, TV infection is associated with conditions such as urethritis, epididymitis, prostatitis, and reduced sperm motility.^1^^,^^2^^,^^7^^,^^10^^,^^11^^,^^15^, ^16^, ^17^ TV may play a role in facilitating the sexual acquisition and transmission of HIV.^18^^,^^19^

Despite the clinical, social, and economic burdens of STIs,^20^, ^21^, ^22^ inadequate understanding of their epidemiology in the global context and their impact on sexual, reproductive, and psychosocial health has lowered their priority on country-level health policy agendas.^23^, ^24^, ^25^ To address this concern, the WHO developed the “Global Health Sector Strategy on STIs”^23^^,^^24^ in alignment with the Sustainable Development Goals^26^ to control or eliminate STIs as a public health concern by 2030 through the integration of preventive, therapeutic, and control frameworks.^23^^,^^24^ The strategy targets a 50% reduction in TV incidence by 2030.^24^

The first strategic direction of this strategy indicates “the need to understand the STI epidemic as a basis for advocacy, political commitment, national planning, resource mobilization and allocation, implementation, and program improvement".^23^ In line with this strategic approach, this study conducted a systematic review aimed at characterizing the epidemiology of TV infection in the Middle East and North Africa (MENA) region. This region, marked at present by armed conflicts, economic crises, and large-scale forced displacements that could impact STI transmission, is also characterized by conservative sexual norms and a scarcity of sexual health programs.^27^, ^28^, ^29^, ^30^, ^31^, ^32^ The specific objectives of this study encompassed a systematic review and synthesis of evidence on TV prevalence, estimation of pooled mean prevalence among diverse populations and subpopulations, and investigation of population–level associations, trends, and sources of heterogeneity across studies.

## Methods

### Data sources and search strategy

Evidence regarding TV prevalence in MENA was systematically reviewed as informed by the Cochrane Collaboration methods.^33^^,^^34^ The findings were reported according to the Preferred Reporting Items for Systematic Reviews and Meta-analyses (PRISMA) guidelines.^34^, ^35^, ^36^ A detailed PRISMA checklist can be found in Supplementary Table S1 of the Supplementary Material. The study protocol was not registered in PROSPERO as it employed published methodologies specifically developed for conducting systematic reviews and meta-analyses of prevalence of STIs, both globally and within the MENA region.^25^^,^^37^, ^38^, ^39^, ^40^, ^41^, ^42^, ^43^, ^44^, ^45^, ^46^, ^47^, ^48^

The literature search included international databases (PubMed, Embase, Scopus, and Web of Science), a regional database (WHO Index Medicus for the Eastern Mediterranean Region), and national databases (the Iraqi Academic Scientific Journals’ database, the Scientific Information Database of Iran, and the PakMediNet of Pakistan), and was conducted up to March 1, 2024. The search criteria were broad, encompassing index terms expanded to cover all subheadings and free-text terms, with no language or year restrictions. The detailed search strategies are included in Supplementary Table S2.

The list of MENA countries considered in this study can be found in Supplementary Box S1. This definition for the MENA region conforms to earlier conventions applied in infectious disease research^28^^,^^47^^,^^49^, ^50^, ^51^, ^52^ and aligns with the definitions set forth by WHO’s Regional Office for the Eastern Mediterranean (WHO-EMRO) and the Joint United Nations Programme on HIV/AIDS (UNAIDS).

### Study selection process and inclusion and exclusion criteria

The search results were imported into EndNote (Thomson Reuters, USA) and underwent deduplication and screening. Initially, titles and abstracts were screened to identify reports that were relevant or potentially relevant. Subsequently, the full-texts of these identified reports were retrieved and screened for relevance. Reports were considered relevant if they presented primary data on TV prevalence in a MENA country, specifically based on laboratory testing. Reports were excluded if they relied on self-reporting of TV infection, involved studies with fewer than 10 participants, or reported testing of upper genital tract specimens. Additionally, case reports, case series, reviews, and editorials were excluded from the analyses. Bibliography screening of relevant articles and literature reviews was also conducted manually to search for additional potentially eligible reports.

Given the documented limitations in diagnostic methods for TV infection,^1^^,^^53^, ^54^, ^55^ a standardized set of inclusion and exclusion criteria for diagnostic methods was developed with the input of an expert in evaluating the validity and reliability of TV diagnostic methods (Professor Jane Schwebke at the University of Alabama at Birmingham) (Supplementary Box S2). These criteria aimed to include only valid and reliable methods. An additional set of rigorous inclusion and exclusion criteria was applied to studies that met the standard criteria. The same expert conducted a detailed and individualized scrutiny of the laboratory methods employed in each included study. This meticulous process resulted in the exclusion of additional studies where the quality of the diagnostic methods could not be fully validated.

To strike a balance between the necessity for valid and reliable diagnostic methods and the need for a sufficiently large number of prevalence measures to facilitate meaningful analyses, the study's analyses were conducted and results were reported using both the standard and stringent inclusion criteria.

In this article, the term “report/publication” refers to a document (such as an article or public health report) containing TV prevalence measures for one or more populations, whereas the term “study” refers to a specific prevalence measure within a particular population. Duplicate study findings were considered only once, prioritizing the more detailed publication.

### Data extraction and data synthesis

Overall prevalence measures (i.e., encompassing the entire sample) and their stratifications were extracted and double-extracted by MH, WSG, AA, and AS from relevant reports, with the condition that each stratum had a sample size of ≥10. This extraction process followed a pre-piloted list of variables, which can be found in Supplementary Box S3. Stratified data extraction adhered to a predefined descendant hierarchy, including population type, sex, age group, and year of data collection. Population type classification, based on the risk of exposure to TV infection, is outlined in Box 1. In cases where studies reported a combined TV prevalence for both men and women, classification was determined by the dominant sex within the sample, provided it exceeded 60%. TV prevalence measures among children aged below 15 years were extracted but were not included in subsequent analyses.

Box 1 **Definitions of population type classifications.**

1. **General populations** (populations at low risk): these include populations at low risk of exposure to *T. vaginalis* such as antenatal clinic attendees, blood donors, and pregnant women, among others.
2. **Intermediate-risk populations**: these include populations who presumably have frequent sexual contact with populations engaging in high sexual risk behavior, and have therefore a higher risk of exposure to *T. vaginalis* than the general population. These comprise prisoners, people who inject drugs, truck drivers, and migrant workers, among others.
3. **Female sex workers:** these include reproductive-age women that are engaged in sex work, that is the exchange of sex for money (sex work as a profession).
4. **Male sex workers and men who have sex with men**: these include men who engage in same-sex sexual activities, specifically anal sex, and men who are engaged in providing sex in return for payment.
5. **Symptomatic women:** these include women with clinical manifestations related to *T. vaginalis* infection or suspected of having *T. vaginalis* infection, such as those with vaginal discharge.
6. **Symptomatic men:** these include men with clinical manifestations related to *T. vaginalis* infection or suspected of having *T. vaginalis* infection, such as those with urethritis.
7. **Symptomatic patients of mixed sexes:** these include populations with undetermined sex with clinical manifestations related to *T. vaginalis* infection or suspected of having *T. vaginalis* infection, such as those with vaginal discharge or urethral discharge.
8. **Infertility clinic attendees:** these were included in a separate category given the uncertainty around their risk of exposure to *T. vaginalis* infection, and the possible biological link between *T. vaginalis* infection and infertility.
9. **Women with miscarriages or ectopic pregnancies:** these were included in a separate category given the uncertainty around their risk of exposure to *T. vaginalis* infection, and the possible biological link between *T. vaginalis* infection and miscarriage or ectopic pregnancy.
10. **STI clinic attendees**: these include patients attending STI clinics.
11. **HIV-positive individuals and individuals in HIV-discordant couples**: these include populations who are HIV-positive or are in a spousal relationship with an HIV-positive individual.
12. **Other populations**: these include populations not satisfying above definitions, or populations with an undetermined risk of acquiring *T. vaginalis* infection.

Abbreviations: STI = Sexually transmitted infection, HIV = Human immunodeficiency virus.

Studies that used the same assay for different biological specimens within a given population were included only once, with a preference for TV detection in vaginal discharge for women, followed by vaginal swabs and then endocervical swabs. For men, TV detection was considered only when done in urine specimens using nucleic acid amplification test (NAAT)/polymerase chain reaction (PCR) methods (Supplementary Box S3). Studies employing different assays on the same biological specimens were extracted separately. This approach was designed to assess the impact of diagnostic methods on measured TV prevalence and to generate adjustment factors^56^, ^57^, ^58^ for estimating TV-related disease burdens in future mathematical modeling studies.

### Precision, risk of bias, and publication bias assessments

Evaluations of the precision and risk of bias (ROB) of relevant studies were conducted by three independent authors (MH, AA, and WSG) with input from LJA. These evaluations drew from the Cochrane approach,^33^ study quality factors relevant to prevalence studies,^59^^,^^60^ and a methodology refined over a series of systematic reviews dedicated to examining the prevalence of STIs.^25^^,^^37^, ^38^, ^39^, ^40^, ^41^, ^42^, ^43^, ^44^, ^45^, ^46^, ^47^, ^48^ This methodology, tailored and refined for the research questions in the present study, consisted of one criterion for assessing study precision and two for evaluating ROB.

Other criteria were not explicitly considered, as they were either already satisfied for quality by the study's design and criteria for inclusion/exclusion, or pertained to a distinct, yet more pertinent, research question within the study, as detailed in Supplementary Table S3. For instance, the evaluation of the validity and reliability of the instrument used to measure TV prevalence was conducted through both the standardized and rigorous sets of inclusion and exclusion criteria for the laboratory methods as described above. Moreover, the impact of assay type on prevalence was investigated through the meta-regression analyses.

Studies were considered to have “high precision” (as opposed to low precision) if they included the testing of at least 200 participants for TV infection. For an expected TV prevalence of 4% in the general population and a sample size of 200, the 95% confidence interval (CI) is 1.1–7.7%, which provides an acceptable level of precision for a prevalence measure.^25^

Studies were categorized as having “low” or “high” ROB within two quality domains: the rigor of sampling methodology (probability-based vs. non-probability-based) and the response rate—proportion of individuals selected to participate in the study who completed the study and provided the required testing data (≥80% vs. <80%). Studies with missing information in any specific domain were classified as having “unclear” ROB for that domain. The results of the precision and ROB assessments were included in the meta-regression analyses to examine their impact on the observed TV prevalence, following established methodologies.^25^^,^^44^^,^^47^^,^^52^

Publication bias in meta-analyses was assessed using Doi plots along with the Luis Furuya-Kanamori (LFK) index whenever the number of pooled studies exceeded three.^61^ An asymmetrical Doi plot indicated potential publication bias; the spread of the prevalence measures may not be due to chance alone.^61^ An LFK index value exceeding ±1 was considered indicative of the presence of publication bias.^61^

### Meta-analyses

The extracted stratified TV prevalence measures were pooled using the DerSimonian-Laird random-effects model,^62^ employing the Freeman-Tukey double arcsine transformation to stabilize the variance,^63^^,^^64^ after examining the applicability of this transformation.^65^ Pooled mean estimates and their corresponding 95% CIs were calculated for each population type and for each assay type, provided that each category contained at least three prevalence measures. To visually represent the pooling outcomes, forest plots were generated.

It has been demonstrated that the DerSimonian-Laird method can introduce bias when pooling a small number of studies in a context of high heterogeneity.^66^, ^67^, ^68^ Consequently, a sensitivity meta-analysis was conducted using the Hartung-Knapp-Sidik-Jonkman method^66^, ^67^, ^68^ to address this limitation whenever the number of studies involved in the meta-analysis was ≤5.

Heterogeneity was assessed using Cochran's Q statistic, with a p-value < 0.1 confirming the presence of heterogeneity across studies. The I^2^ statistic was used to quantify the extent of between-study variation attributed to true differences in prevalence across studies rather than chance. The prediction interval was employed to estimate the distribution of true prevalence around the pooled mean.^62^^,^^69^ Meta-analyses were conducted using R version 4.41.3,^70^ utilizing the “meta” package.^71^

Recognizing the existence of heterogeneity among the prevalence measures, the pooled means should be regarded as an average summary measure.^25^^,^^37^ The meta-regression analyses described below investigated and explained the sources of this heterogeneity in terms of epidemiological factors and study methods.

### Meta-regressions

Both univariable and multivariable random-effects meta-regression analyses were conducted to explore sources of between-study heterogeneity and identify factors (predictors) associated with TV prevalence. Specifically, linear random-effects meta-regressions, including a random intercept term, were performed on the log-transformed TV prevalence measures.^72^ A log transformation, rather than a logit transformation, was utilized to generate prevalence ratios, which offer a clearer and more accessible epidemiological interpretation compared to prevalence odds ratios. Linearity assumption of the meta-regression model was assessed using the Ramsey RESET Test.^73^ Both the random error and random-effects term were assumed to be normally distributed.

The selection of factors in the regressions was based on their epidemiological relevance and prior understanding of HIV/STI epidemiology,^25^^,^^37^^,^^39^^,^^43^^,^^44^ as outlined in Supplementary Box S4. Consideration was also given to potential biases stemming from the application of study methods.

Based on the number of predictors, the exclusion of variables with collinearities, and the specific goal of the model, which is interpretation, all variables with a p-value ≤ 0.2 in the univariable analyses were considered eligible for inclusion in the multivariable model, and the results were reported for this model. In the multivariable model, a p-value of ≤0.05 was deemed to indicate a statistically significant association with TV prevalence. The goodness of fit was determined by the adjusted R^2^.

For the year of data collection, which had 19% missing values, imputation was conducted using the year of publication data, adjusted by the median difference between the year of publication and the year of data collection. Meta-regressions were conducted in Stata/SE version 16^74^ using the “metareg” package.^72^

### Role of the funding source

The funders of the study had no role in study design, data collection, data analysis, data interpretation, or writing of the report. The corresponding authors had full access to all the data in the study and had final responsibility for the decision to submit for publication.

## Results

### Search results and scope of evidence

**Fig. 1:**
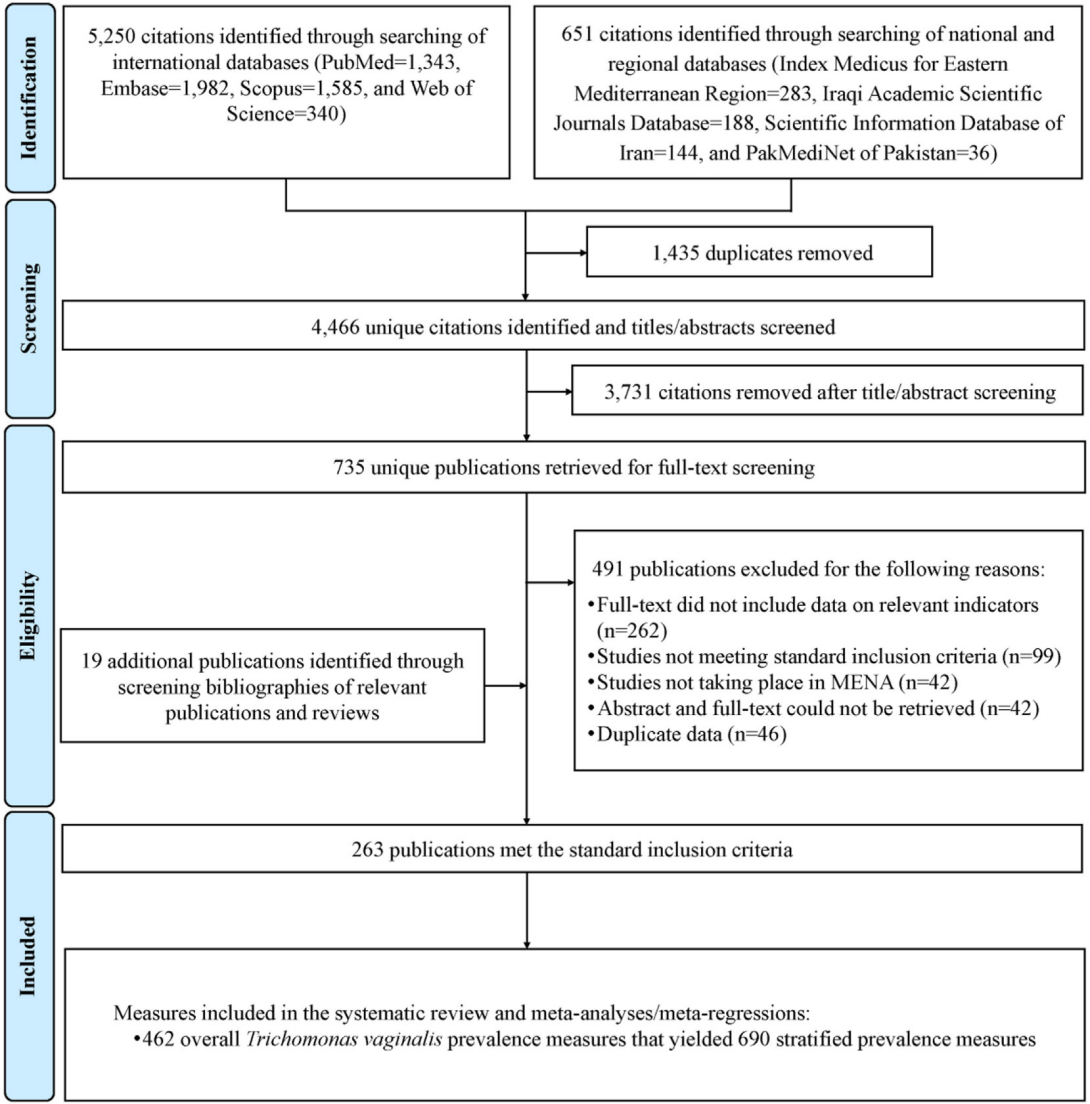
Flowchart depicting the study selection process for assessing *Trichomonas vaginalis* prevalence in the Middle East and North Africa, following PRISMA guidelines.^35^^,^^36^ Abbreviations: MENA = Middle East and North Africa.

Fig. 1 illustrates the PRISMA selection process employed in this study. The searches conducted in international databases yielded a total of 5250 publications, with 1343 retrieved from PubMed, 1982 from Embase, 1585 from Scopus, and 340 from Web of Science. The searches carried out in regional and national databases identified an additional 651 publications. These were sourced from the Index Medicus for Eastern Mediterranean Region (283 publications), Iraqi Academic Scientific Journals Database (188 publications), Scientific Information Database of Iran (144 publications), and PakMediNet of Pakistan (36 publications).

Following the elimination of duplicate publications, title and abstract screening, as well as full-text screening, a total of 244 publications met the standard inclusion criteria (Fig. 1). An additional 19 relevant publications were identified through the examination of bibliographies in relevant articles and reviews. In summary, a total of 263 publications satisfied the standard inclusion criteria and were thus included in this study, as listed in Supplementary Box S5. These included publications were then subjected to the stringent inclusion criteria, leading to the exclusion of 115 additional publications. This rigorous process resulted in the selection of 148 publications that met the stringent inclusion criteria, as listed in Supplementary Box S6.

Accordingly, 263 publications met the standard inclusion criteria from which 462 overall TV prevalence measures (i.e., encompassing the entire sample) were extracted, including 690 stratified prevalence measures (Fig. 1). Moreover, 148 publications met the stringent inclusion criteria from which 263 overall TV prevalence measures were extracted, including 400 stratified prevalence measures.

The evidence pertaining to TV prevalence, in accordance with the standard inclusion criteria, incorporated data from 13 out of the 23 MENA countries. Iran was the primary contributor, with 87 publications reporting 148 overall prevalence measures derived from 149,568 individuals. Iraq closely followed, with 87 publications documenting 139 overall TV prevalence measures derived from 36,427 individuals. All studies tested urogenital specimens, and nearly all studies were on women (440, 95.2%). The prevalence measures covered a broad timeframe, with the earliest measure published in 1962, but the majority of them were recent. Specifically, a total of 291 (63.0%) overall prevalence measures were reported after the year 2010.

### *Trichomonas vaginalis* prevalence overview

Table 1 presents a summary of the stratified TV prevalence measures, classified by population type and assay type, showcasing both the interquartile range and median values. While a wide array of populations was covered in the available prevalence measures, 46.8% pertained to symptomatic women, followed by 38.6% relating to general populations. A smaller subset, constituting only 2.0% of the prevalence measures, specifically focused on female sex workers.

**Table 1:** Results of meta-analyses for *Trichomonas vaginalis* prevalence in the Middle East and North Africa across diverse populations, stratified by assay type.

Population type	Outcome measures	Sample size	TV prevalence (%)		Pooled mean TV prevalence	Heterogeneity measures		
	Total n	Total N	IQR	Median	Mean (%) (95% CI)	Q^a^ (p-value)	I^2^^b^ (%) (95% CI)	Prediction interval^c^ (%)
**General populations**								
NAAT/PCR	30	10,625	0.7–9.3	2.4	4.3 (2.2–7.0)	401.9 (p < 0.001)	92.8 (90.8–94.4)	0.0–25.5
Culture	104	23,166	1.0–7.8	3.8	5.0 (3.6–6.5)	1204.0 (p < 0.001)	91.4 (90.2–92.6)	0.0–26.4
Wet mount	132	75,409	1.3–8.5	3.4	4.5 (3.4–5.8)	3884.4 (p < 0.001)	96.6 (96.3–96.9)	0.0–24.4
**Overall**	**266**	**109,200**	**1.1–8.5**	**3.6**	**4.7 (3.9–5.6)**	**5774.9 (p < 0.001)**	**95.4 (95.1–95.7)**	**0.0–24.9**
**Intermediate risk populations**								
NAAT/PCR	4	847	0.0–64.9	32.5	21.2 (0.0–69.9)	144.8 (p < 0.001)	97.9 (96.5–98.8)	0.0–100.0
Culture	3	553	14.6–23.5	18.9	17.4 (8.0–29.4)	12.2 (p = 0.002)	83.7 (50.7–94.6)	0.0–100.0
Wet mount	3	960	8.5–17.8	9.1	13.3 (4.5–25.8)	30.9 (p < 0.001)	93.5 (84.5–97.3)	0.0–100.0
**Overall**	**10**	**2360**	**8.1–27.6**	**14.6**	**17.2 (5.4–33.6)**	**310.4 (p < 0.001)**	**97.1 (96.0–97.9)**	**0.0–83.6**
**Female sex workers**								
NAAT/PCR	9	3481	5.7–14.9	12.2	12.3 (6.6–19.4)	257.0 (p < 0.001)	96.9 (95.5–97.8)	0.0–43.9
Culture	5	1621	2.9–11.6	11.2	7.2 (2.8–13.3)	64.1 (p < 0.001)	93.8 (88.3–96.7)	0.0–38.3
**Overall**	**14**	**5102**	**5.3–13.1**	**11.7**	**10.3 (6.2–15.3)**	**342.4 (p < 0.001)**	**96.2 (94.9–97.2)**	**0.0–35.5**
**Symptomatic women**								
NAAT/PCR	49	7272	13.5–32.0	21.4	23.5 (18.7–28.8)	1538.8 (p < 0.001)	96.9 (96.4–97.3)	0.5–63.0
Culture	102	24,435	6.0–20.9	10.3	14.0 (11.0–17.1)	3063.7 (p < 0.001)	96.7 (96.3–97.0)	0.0–52.4
Wet mount	167	152,769	4.8–20.0	11.3	11.5 (9.6–13.6)	8691.1 (p < 0.001)	98.1 (98.0–98.2)	0.0–43.2
Rapid test	5	650	10.2–14.0	12.5	11.0 (8.1–14.3)	6.4 (p = 0.17)	37.7 (0.0–76.8)	4.0–20.8
**Overall**	**323**	**185,126**	**5.8–21.9**	**12.4**	**13.9 (12.3–15.6)**	**18,942.9 (p < 0.001)**	**98.3 (98.2–98.4)**	**0.0–49.8**
**Symptomatic men**								
NAAT/PCR	2	175	16.9–17.4	17.2	17.1 (11.9–23.6)^d^	–	–	–
Wet mount	1	90	–	–	10.0 (4.7–18.1)	–	–	–
**Overall**	**3**	**265**	**13.4–17.2**	**16.7**	**14.6 (10.0–19.8)**	**2.6 (p = 0.28)**	**21.8 (0.0–91.9)**	**0.0–67.8**
**Symptomatic patients (mixed sexes)**								
NAAT/PCR	3	108	3.6–8.5	7.1	4.2 (0.0–13.8)	5.9 (p = 0.054)	65.8 (0.0–90.2)	0.0–100.0
**Overall**	**3**	**108**	**3.6–8.5**	**7.1**	**4.2 (0.0–13.8)**	**5.9 (p = 0.054)**	**65.8 (0.0–90.2)**	**0.0–100.0**
**Infertility clinic attendees**								
NAAT/PCR	2	238	3.3–6.6	5.0	3.4 (1.5–6.5)^d^	–	–	–
Culture	3	287	5.9–21.6	10.0	12.8 (0.8–34.7)	38.9 (p < 0.001)	94.9 (88.3–97.7)	0.0–100.0
Wet mount	2	150	2.9–5.4	4.2	4.7 (1.9–9.4)^d^	–	–	–
**Overall**	**7**	**675**	**1.8–9.2**	**6.7**	**7.4 (1.9–15.5)**	**84.5 (p < 0.001)**	**92.9 (87.9–95.8)**	**0.0–44.5**
**Women with miscarriages or ectopic pregnancies**								
NAAT/PCR	4	319	0.0–3.5	1.4	1.1 (0.0–4.3)	6.9 (p = 0.074)	56.7 (0.0–85.6)	0.0–21.7
Culture	1	81	–	–	11.1 (5.2–20.0)	–	–	–
Wet mount	2	180	1.7–5.0	3.4	2.2 (0.6–5.6)^d^	–	–	–
**Overall**	**7**	**580**	**0.0–6.1**	**2.8**	**2.3 (0.1–6.3)**	**28.7 (p < 0.001)**	**79.1 (57.1–89.8)**	**0.0–20.7**
**STI clinic attendees**								
NAAT/PCR	5	2891	0.8–1.2	1.2	1.1 (0.4–2.1)	14.7 (p = 0.005)	72.7 (31.6–89.1)	0.0–5.5
Culture	1	500	–	–	3.2 (1.8–5.1)	–	–	–
Wet mount	2	560	2.6–5.0	3.8	2.9 (1.6–4.6)^d^	–	–	–
**Overall**	**8**	**3951**	**1.1–3.1**	**1.9**	**1.6 (0.8–2.7)**	**38.3 (p < 0.001)**	**81.7 (65.1–90.4)**	**0.0–6.1**
**HIV-positive individuals and individuals in HIV-discordant couples**								
NAAT/PCR	1	273	–	–	38.1 (32.3–44.1)	–	–	–
Culture	1	50	–	–	60.0 (45.2–73.6)	–	–	–
**Overall**	**2**	**323**	**43.6–54.5**	**49.1**	**41.5 (36.1–47.0)**^d^	**–**	**–**	**–**
**Other populations**^e^								
NAAT/PCR	5	1319	4.8–4.9	4.8	5.3 (3.7–7.2)	6.1 (p = 0.20)	33.8 (0.0–75.0)	1.4–11.2
Culture	23	8905	1.7–8.4	3.8	4.8 (2.3–8.1)	529.0 (p < 0.001)	95.8 (94.7–96.7)	0.0–26.3
Wet mount	19	42,991	2.7–5.9	4.4	5.5 (2.6–9.1)	678.5 (p < 0.001)	97.3 (96.7–97.9)	0.0–27.9
**Overall**	**47**	**53,215**	**2.2–7.8**	**4.4**	**5.1 (3.3–7.1)**	**1602.7 (p < 0.001)**	**97.1 (96.7–97.5)**	**0.0–23.5**

Prevalence measures conformed to the standard inclusion criteria for diagnostic methods.

Abbreviation: CI = Confidence interval, HIV = Human immunodeficiency virus, IQR =Interquartile range, NAAT = Nucleic acid amplification test, PCR = Polymerase chain reaction, STI = Sexually transmitted infection, TV = *Trichomonas vaginalis*.

A minimum of three studies was required to perform a meta-analysis.

a Q: The Cochran's Q statistic is a measure assessing the existence of heterogeneity in pooled outcome measures, here TV prevalence.

b I^2^: A measure that assesses the magnitude of between-study variation that is due to true differences in TV prevalence across studies rather than chance.

c Prediction interval: A measure that estimates the distribution (95% interval) of true TV prevalence around the estimated mean.

d Two prevalence measures are not sufficient to conduct a random-effects meta-analysis. The pooled measure was calculated as the arithmetic mean of the two measures and their 95% confidence intervals.

e Other populations include populations with an undetermined risk of acquiring TV infection such as women with premature labor, cancer patients, patients suffering from diabetes, and mixed at-risk populations, among others.

### Precision, risk of bias, and publication bias assessments

Supplementary Table S4 provides a summary of the precision and ROB assessments of each included study. Among the extracted overall prevalence measures, 259 (56.1%) were derived from studies demonstrating high precision, defined by sample sizes of ≥200 participants. The vast majority of studies (419, 90.7%) employed non-probability-based sampling methods. In 443 studies (95.9%), the response rate was classified as unclear. Only nine studies (1.9%) exhibited low ROB in both quality domains, while three studies (0.6%) exhibited high ROB in both quality domains.

Publication bias assessment is summarized in Supplementary Table S5. While some meta-analyses showed no evidence of publication bias, others displayed asymmetrical Doi plots and LFK index values exceeding ±1, indicating the presence of publication bias.

### Pooled mean estimates of *Trichomonas vaginalis* prevalence

The pooled mean TV prevalence by population type and assay type under the standard inclusion criteria is summarized in Table 1. The mean TV prevalence was 4.7% (95% CI: 3.9–5.6%) among the general population (99.2% of studies on general populations were on women), 17.2% (95% CI: 5.4–33.6%) among intermediate-risk populations, 10.3% (95% CI: 6.2–15.3%) among female sex workers, 13.9% (95% CI: 12.3–15.6%) among symptomatic women, 14.6% (95% CI: 10.0–19.8%) among symptomatic men, 7.4% (95% CI: 1.9–15.5%) among infertility clinic attendees, 2.3% (95% CI: 0.1–6.3%) among women with miscarriages or ectopic pregnancies, and 1.6% (95% CI: 0.8–2.7%) among STI clinic attendees.

The pooled mean TV prevalence by population type and assay type under the stringent inclusion criteria is summarized in Supplementary Table S6. The results showed similar estimates to those under the standard inclusion criteria, but with wider 95% CIs due to the smaller number of included studies. The mean TV prevalence was 5.7% (95% CI: 4.3–7.2%) among general populations, 32.6% (95% CI: 5.7–67.8%) among intermediate-risk populations, 9.1% (95% CI: 3.2–17.5%) among female sex workers, 14.5% (95% CI: 12.4–16.7%) among symptomatic women, 14.6% (95% CI: 10.0–19.8%) among symptomatic men, 5.0% (95% CI: 1.9–9.1%) among infertility clinic attendees, 2.6% (95% CI: 0.0–7.3%) among women with miscarriages or ectopic pregnancies, and 2.9% (95% CI: 2.1–3.9%) among STI clinic attendees.

The sensitivity analysis using the Hartung-Knapp-Sidik-Jonkman method^66^, ^67^, ^68^ whenever the number of studies involved in the meta-analysis was ≤5 is shown in Supplementary Table S7. The analysis showed similar pooled estimates but with more conservative 95% CIs and p-values compared to the main analysis.

Forest plots depicting the outcomes of the meta-analyses are found in Supplementary Fig. S1. The majority of these meta-analyses exhibited heterogeneity (p-value < 0.1). This heterogeneity was primarily attributed to true variations in prevalence rather than to random chance, as evidenced by the I^2^ values exceeding 50%. This observation was reinforced by the wide prediction intervals, underscoring the heterogeneity in TV prevalence across the included studies.

### Predictors of *Trichomonas vaginalis* prevalence and sources of between-study heterogeneity

The outcomes of both univariable and multivariable meta-regression analyses for TV prevalence under the standard inclusion criteria are detailed in Table 2, Table 3, and Supplementary Table S8. Two distinct multivariable models were employed: one incorporating the year of data collection as a categorical variable, and another treating it as a linear term. The Ramsey RESET test indicated no evidence of a non-linear relationship (p-value = 0.599). Additional sensitivity analyses were conducted to address potential collinearity between the MENA subregion variable and national income, as well as between the year of data collection and year of publication. Remarkably, all models consistently explained more than 40% of the variation in prevalence observed across the studies and generated similar results.

The results indicated that, in comparison to the general population, TV prevalence was higher among intermediate-risk populations, female sex workers, symptomatic women, and HIV-positive individuals and individuals in HIV-discordant couples (Table 2). However, TV prevalence was lower among STI clinic attendees. There was no evidence of variation in prevalence by sex. TV prevalence decreased with increasing age and exhibited variations across the MENA subregions. Notably, upper-middle-income countries and high-income countries showed lower TV prevalence when contrasted with lower income countries (Table 3). TV prevalence declined at a rate of approximately 1% per calendar year.

**Table 2:** Univariable and multivariable meta-regression analyses for *Trichomonas vaginalis* prevalence in the Middle East and North Africa, incorporating MENA subregion and year of data collection as variables.

	Outcome measures	Sample size	Univariable analysis				Multivariable analyses			
	Total n	Total N	PR (95% CI)	p-value	LR test p-value	Adjusted R^2^	Model 1^a^		Model 2^b^	
							APR (95% CI)	p-value	APR (95% CI)	p-value
Population characteristics										
Population type										
General populations	266	109,200	1.00	–	<0.001	20.2	1.00	–	1.00	–
Intermediate-risk populations	10	2360	3.29 (1.67–6.48)	0.001			2.25 (1.25–4.03)	0.007	2.15 (1.20–3.85)	0.010
Female sex workers	14	5102	1.87 (1.08–3.24)	0.026			3.30 (1.76–6.17)	<0.001	3.06 (1.64–5.70)	<0.001
Symptomatic women	323	185,126	2.59 (2.16–3.11)	<0.001			1.93 (1.63–2.28)	<0.001	1.95 (1.65–2.30)	<0.001
Symptomatic men	3	265	3.01 (0.94–9.61)	0.064			1.26 (0.47–3.39)	0.65	1.11 (0.41–3.01)	0.83
Symptomatic patients (mixed sexes)	3	108	1.71 (0.35–8.32)	0.51			0.59 (0.15–2.40)	0.46	0.65 (0.16–2.65)	0.55
Infertility clinic attendees	7	675	1.33 (0.57–3.11)	0.50			0.52 (0.25–1.09)	0.084	0.51 (0.24–1.08)	0.08
Women with miscarriages or ectopic pregnancies	7	580	1.18 (0.40–3.49)	0.77			0.54 (0.21–1.38)	0.20	0.58 (0.22–1.49)	0.26
STI clinic attendees	8	3951	0.35 (0.16–0.75)	0.007			0.29 (0.15–0.56)	<0.001	0.29 (0.15–0.55)	<0.001
HIV-positive individuals and individuals in HIV-discordant couples	2	323	9.96 (2.51–39.58)	0.001			8.73 (2.77–27.52)	<0.001	8.51 (2.70–26.82)	<0.001
Other populations^c^	47	53,215	1.01 (0.72–1.43)	0.94			0.99 (0.73–1.34)	0.95	1.00 (0.74–1.35)	0.99
Age group										
<20 years	27	1260	1.00	–	<0.001	3.4	1.00	–	1.00	–
20–29 years	50	5745	1.17 (0.62–2.19)	0.64			1.33 (0.80–2.21)	0.27	1.34 (0.81–2.24)	0.25
30–39 years	47	5009	1.11 (0.59–2.11)	0.74			1.20 (0.72–2.01)	0.49	1.21 (0.72–2.02)	0.47
40–49 years	29	2129	0.74 (0.36–1.52)	0.41			0.77 (0.43–1.39)	0.39	0.77 (0.43–1.39)	0.39
≥50 years	15	2494	0.28 (0.10–0.77)	0.014			0.49 (0.21–1.13)	0.095	0.49 (0.21–1.13)	0.095
Mixed ages	522	344,268	0.66 (0.38–1.15)	0.15			0.85 (0.54–1.34)	0.49	0.85 (0.54–1.34)	0.49
Sex										
Women	669	356,664	1.00	–	0.74	0.0	–	–	–	–
Men	16	4040	0.82 (0.45–1.50)	0.51			–	–	–	–
Mixed sexes	5	201	0.75 (0.21–2.68)	0.66			–	–	–	–
MENA subregion^d^										
Fertile crescent	413	65,583	1.00	–	<0.001	15.0	1.00	–	1.00	–
Horn of Africa	22	6276	1.53 (0.96–2.44)	0.075			1.41 (0.94–2.11)	0.10	1.40 (0.93–2.12)	0.11
Maghreb	13	7927	0.78 (0.43–1.41)	0.41			0.74 (0.43–1.28)	0.29	0.80 (0.47–1.38)	0.43
Gulf	10	124,975	0.21 (0.10–0.42)	<0.001			0.22 (0.12–0.40)	<0.001	0.23 (0.13–0.42)	<0.001
Iran	205	149,568	0.44 (0.36–0.53)	<0.001			0.69 (0.58–0.82)	<0.001	0.70 (0.58–0.83)	<0.001
Pakistan	27	6576	0.44 (0.27–0.71)	0.001			0.43 (0.27–0.69)	<0.001	0.43 (0.27–0.69)	<0.001
National income										
LIC and LMIC	187	47,363	1.00	–	<0.001^e^	5.5	–	–	–	–
UMIC	493	188,567	0.63 (0.52–0.77)	<0.001			–	–	–	–
HIC	10	124,975	0.20 (0.09–0.42)	<0.001			–	–	–	–
Study methodology characteristics										
Assay type										
NAAT/PCR	114	27,548	1.00	–	0.023	1.2	1.00	–	1.00	–
Culture	243	59,598	0.75 (0.57–0.98)	0.032			0.81 (0.65–1.01)	0.064	0.77 (0.62–0.96)	0.023
Wet mount	328	273,109	0.67 (0.52–0.87)	0.003			0.67 (0.54–0.83)	<0.001	0.64 (0.51–0.79)	<0.001
Rapid test	5	650	1.02 (0.37–2.82)	0.96			0.56 (0.25–1.22)	0.14	0.51 (0.23–1.12)	0.094
Sample size										
<200	272	21,288	1.00	–	<0.001	19.3	1.00	–	1.00	–
≥200	418	339,617	0.39 (0.33–0.46)	<0.001			0.44 (0.38–0.52)	<0.001	0.44 (0.38–0.52)	<0.001
Sampling method										
Probability based	50	18,061	1.00	–	0.15	0.2	1.00	–	1.00	–
Non-probability based	640	342,844	1.30 (0.91–1.85)	0.15			0.91 (0.67–1.24)	0.56	0.87 (0.64–1.18)	0.36
Response rate										
≥80%	25	10,042	1.00	–	0.037	0.9	1.00	–	1.00	–
<80%	6	505	4.05 (1.31–12.54)	0.015			3.29 (1.27–8.53)	0.015	3.88 (1.49–10.12)	0.006
Unclear	659	350,358	1.61 (0.97–2.69)	0.066			1.60 (0.99–2.61)	0.057	1.69 (1.04–2.77)	0.035
Temporal trend										
Year of data collection category										
<2000	102	35,646	1.00	–	0.088	0.5	1.00	–	–	–
2000–2009	253	244,359	0.75 (0.57–0.99)	0.042			0.71 (0.57–0.89)	0.003	–	–
≥2010	335	80,900	0.89 (0.68–1.15)	0.37			0.79 (0.63–0.99)	0.042	–	–
Year of data collection	690	360,905	0.99 (0.98–1.00)	0.24	0.24	0.0	–	–	0.99 (0.98–1.00)	0.017
Year of publication category										
<2005	110	36,657	1.00	–	0.84^f^	0.0	–	–	–	–
2005–2014	291	133,322	0.93 (0.71–1.21)	0.58			–	–	–	–
≥2015	289	190,926	0.93 (0.72–1.22)	0.61			–	–	–	–
Year of publication	690	360,905	0.99 (0.98–1.00)	0.055	0.055^f^	0.4	–	–	–	–

Prevalence measures conformed to the standard inclusion criteria for diagnostic methods.

Abbreviations: APR = Adjusted prevalence ratio, CI = Confidence interval, HIC = High-income country, HIV = Human immunodeficiency virus, MENA = Middle East and North Africa, NAAT = Nucleic acid amplification test, LIC = Low-income country, LMIC = Low-middle-income country, LR test = Likelihood ratio test, PCR = Polymerase chain reaction, PR = Prevalence ratio, STI = Sexually transmitted infection, UMIC = Upper-middle-income country.

The PR represents the exponentiated beta coefficient calculated by the meta-regression model.

a Adjusted R^2^ in the final multivariable model 1 = 45.9%. Model 1 includes population type, age group, MENA subregion, assay type, sample size, sampling method, response rate, and year of data collection as a categorical variable. Other variables were not included either because their p-values in the univariable model were greater than 0.2 or due to collinearity with another variable included in the model.

b Adjusted R^2^ in the final multivariable model 2 = 45.7%. Model 2 includes population type, age group, MENA subregion, assay type, sample size, sampling method, response rate, and year of data collection as a continuous linear term. Other variables were not included either because their p-values in the univariable model were greater than 0.2 or due to collinearity with another variable included in the model.

c Other populations include populations with an undetermined risk of acquiring *Trichomonas vaginalis* infection such as women with premature labor, cancer patients, patients suffering from diabetes, and mixed at-risk populations, among others.

d Countries included in each MENA subregion are as follows: Fertile Crescent (Egypt, Iraq, Jordan, Lebanon, Palestine, Syria); Horn of Africa (Djibouti, Somalia, Sudan, Yemen); Maghreb (Algeria, Libya, Morocco, Tunisia); and Gulf (Bahrain, Kuwait, Oman, Qatar, Saudi Arabia, United Arab Emirates).

e National income was not included in the multivariable model due to collinearity with MENA subregion variable.

f Year of publication was not included in the multivariable model due to collinearity with year of data collection variable.

**Table 3:** Sensitivity analysis. Univariable and multivariable meta-regression analyses for *Trichomonas vaginalis* prevalence in the Middle East and North Africa, incorporating national income (in place of MENA subregion) and year of data collection as variables.

	Outcome measures	Sample size	Univariable analysis				Multivariable analyses			
	Total n	Total N	PR (95% CI)	p-value	LR test p-value	Adjusted R^2^	Model 1^a^		Model 2^b^	
							APR (95% CI)	p-value	APR (95% CI)	p-value
Population characteristics										
Population type										
General populations	266	109,200	1.00	–	<0.001	20.2	1.00	–	1.00	–
Intermediate-risk populations	10	2360	3.29 (1.67–6.48)	0.001			2.15 (1.20–3.86)	0.010	2.05 (1.14–3.67)	0.016
Female sex workers	14	5102	1.87 (1.08–3.24)	0.026			2.30 (1.29–4.12)	0.005	2.19 (1.23–3.90)	0.008
Symptomatic women	323	185,126	2.59 (2.16–3.11)	<0.001			2.13 (1.81–2.51)	<0.001	2.14 (1.82–2.52)	<0.001
Symptomatic men	3	265	3.01 (0.94–9.61)	0.064			1.37 (0.50–3.72)	0.54	1.19 (0.44–3.23)	0.74
Symptomatic patients (mixed sexes)	3	108	1.71 (0.35–8.32)	0.51			0.80 (0.20–3.27)	0.76	0.89 (0.22–3.63)	0.87
Infertility clinic attendees	7	675	1.33 (0.57–3.11)	0.50			0.55 (0.26–1.17)	0.12	0.54 (0.26–1.15)	0.11
Women with miscarriages or ectopic pregnancies	7	580	1.18 (0.40–3.49)	0.77			0.61 (0.23–1.59)	0.31	0.66 (0.25–1.71)	0.39
STI clinic attendees	8	3951	0.35 (0.16–0.75)	0.007			0.32 (0.16–0.62)	0.001	0.32 (0.16–0.61)	0.001
HIV-positive individuals and individuals in HIV-discordant couples	2	323	9.96 (2.51–39.58)	0.001			8.15 (2.56–25.92)	<0.001	8.00 (2.51–25.45)	<0.001
Other populations^c^	47	53,215	1.01 (0.72–1.43)	0.94			1.03 (0.76–1.40)	0.83	1.04 (0.77–1.41)	0.81
Age group										
<20 years	27	1260	1.00	–	<0.001	3.4	1.00	–	1.00	–
20–29 years	50	5745	1.17 (0.62–2.19)	0.64			1.35 (0.81–2.26)	0.24	1.38 (0.83–2.31)	0.22
30–39 years	47	5009	1.11 (0.59–2.11)	0.74			1.24 (0.74–2.08)	0.42	1.26 (0.75–2.11)	0.39
40–49 years	29	2129	0.74 (0.36–1.52)	0.41			0.78 (0.43–1.42)	0.42	0.79 (0.44–1.43)	0.44
≥50 years	15	2494	0.28 (0.10–0.77)	0.014			0.45 (0.19–1.05)	0.064	0.46 (0.20–1.06)	0.069
Mixed ages	522	344,268	0.66 (0.38–1.15)	0.15			0.76 (0.48–1.19)	0.22	0.76 (0.49–1.20)	0.24
Sex										
Women	669	356,664	1.00	–	0.74	0.0	–	–	–	–
Men	16	4040	0.82 (0.45–1.50)	0.51			–	–	–	–
Mixed sexes	5	201	0.75 (0.21–2.68)	0.66			–	–	–	–
MENA subregion^d^										
Fertile crescent	413	65,583	1.00	–	<0.001^e^	15.0	–	–	–	–
Horn of Africa	22	6276	1.53 (0.96–2.44)	0.075			–	–	–	–
Maghreb	13	7927	0.78 (0.43–1.41)	0.41			–	–	–	–
Gulf	10	124,975	0.21 (0.10–0.42)	<0.001			–	–	–	–
Iran	205	149,568	0.44 (0.36–0.53)	<0.001			–	–	–	–
Pakistan	27	6576	0.44 (0.27–0.71)	0.001			–	–	–	–
National income										
LIC and LMIC	187	47,363	1.00	–	<0.001	5.5	1.00	–	1.00	–
UMIC	493	188,567	0.63 (0.52–0.77)	<0.001			0.72 (0.61–0.86)	<0.001	0.72 (0.61–0.86)	<0.001
HIC	10	124,975	0.20 (0.09–0.42)	<0.001			0.20 (0.11–0.37)	<0.001	0.21 (0.11–0.38)	<0.001
Study methodology characteristics										
Assay type										
NAAT/PCR	114	27,548	1.00	–	0.023	1.2	1.00	–	1.00	–
Culture	243	59,598	0.75 (0.57–0.98)	0.032			0.80 (0.63–1.00)	0.048	0.75 (0.60–0.95)	0.014
Wet mount	328	273,109	0.67 (0.52–0.87)	0.003			0.67 (0.54–0.83)	<0.001	0.63 (0.51–0.78)	<0.001
Rapid test	5	650	1.02 (0.37–2.82)	0.96			0.48 (0.22–1.06)	0.068	0.44 (0.20–0.97)	0.042
Sample size										
<200	272	21,288	1.00	–	<0.001	19.3	1.00	–	1.00	–
≥200	418	339,617	0.39 (0.33–0.46)	<0.001			0.44 (0.38–0.52)	<0.001	0.45 (0.38–0.52)	<0.001
Sampling method										
Probability based	50	18,061	1.00	–	0.15	0.2	1.00	–	1.00	–
Non-probability based	640	342,844	1.30 (0.91–1.85)	0.15			0.93 (0.68–1.27)	0.66	0.89 (0.65–1.21)	0.45
Response rate										
≥80%	25	10,042	1.00	–	0.037	0.9	1.00	–	1.00	–
<80%	6	505	4.05 (1.31–12.54)	0.015			6.00 (2.35–15.35)	<0.001	7.18 (2.81–18.36)	<0.001
Unclear	659	350,358	1.61 (0.97–2.69)	0.066			2.12 (1.32–3.41)	0.002	2.27 (1.41–3.66)	0.001
Temporal trend										
Year of data collection category										
<2000	102	35,646	1.00	–	0.088	0.5	1.00	–	–	–
2000–2009	253	244,359	0.75 (0.57–0.99)	0.042			0.69 (0.55–0.86)	0.001	–	–
≥2010	335	80,900	0.89 (0.68–1.15)	0.37			0.76 (0.61–0.94)	0.013	–	–
Year of data collection	690	360,905	0.99 (0.98–1.00)	0.24	0.24	0.0	–	–	0.99 (0.98–1.00)	0.002
Year of publication category										
<2005	110	36,657	1.00	–	0.84^f^	0.0	–	–	–	–
2005–2014	291	133,322	0.93 (0.71–1.21)	0.58			–	–	–	–
≥2015	289	190,926	0.93 (0.72–1.22)	0.61			–	–	–	–
Year of publication	690	360,905	0.99 (0.98–1.00)	0.055	0.055^f^	0.4	–	–	–	–

Prevalence measures conformed to the standard inclusion criteria for diagnostic methods.

Abbreviations: APR = Adjusted prevalence ratio, CI = Confidence interval, HIC = High-income country, HIV = Human immunodeficiency virus, MENA = Middle East and North Africa, NAAT = Nucleic acid amplification test, LIC = Low-income country, LMIC = Low-middle-income country, LR test = Likelihood ratio test, PCR = Polymerase chain reaction, PR = Prevalence ratio, STI = Sexually transmitted infection, UMIC = Upper-middle-income country.

The PR represents the exponentiated beta coefficient calculated by the meta-regression model.

a Adjusted R^2^ in the final multivariable model 1 = 44.7%. Model 1 includes population type, age group, national income, assay type, sample size, sampling method, response rate, and year of data collection as a categorical variable. Other variables were not included either because their p-values in the univariable model were greater than 0.2 or due to collinearity with another variable included in the model.

b Adjusted R^2^ in the final multivariable model 2 = 44.7%. Model 2 includes population type, age group, national income, assay type, sample size, sampling method, response rate, and year of data collection as a continuous linear term. Other variables were not included either because their p-values in the univariable model were greater than 0.2 or due to collinearity with another variable included in the model.

c Other populations include populations with an undetermined risk of acquiring *Trichomonas vaginalis* infection such as women with premature labor, cancer patients, patients suffering from diabetes, and mixed at-risk populations, among others.

d Countries included in each MENA subregion are as follows: Fertile Crescent (Egypt, Iraq, Jordan, Lebanon, Palestine, Syria); Horn of Africa (Djibouti, Somalia, Sudan, Yemen); Maghreb (Algeria, Libya, Morocco, Tunisia); and Gulf (Bahrain, Kuwait, Oman, Qatar, Saudi Arabia, United Arab Emirates).

e MENA subregion was not included in the multivariable model due to collinearity with national income variable.

f Year of publication was not included in the multivariable model due to collinearity with year of data collection variable.

In terms of the impact of study methods on TV prevalence, TV prevalence varied based on the type of assay used in the study (Table 2). Studies employing NAAT/PCR assays reported higher TV prevalence compared to other diagnostic methods. A small-study effect was evident, with studies involving larger sample sizes (≥200 participants) reporting approximately 55% lower TV prevalence. Studies with low (<80%) or unclear response rates showed higher TV prevalence. However, there was no evidence indicating variation in TV prevalence based on the sampling method.

The results of both univariable and multivariable meta-regression analyses for TV prevalence under the stringent inclusion criteria are presented in Table 4. These analyses yielded outcomes similar to those observed under the standard inclusion criteria. However, the prevalence ratios in this case had wider 95% CIs due to the smaller number of included studies, and as a result, statistical significance was not attained for several of the associations.

**Table 4:** Univariable and multivariable meta-regression analyses for *Trichomonas vaginalis* prevalence in the Middle East and North Africa, utilizing exclusively prevalence measures that met the stringent inclusion criteria for diagnostic methods.

	Outcome measures	Sample size	Univariable analysis				Multivariable analyses			
	Total n	Total N	PR (95% CI)	p-value	LR test p-value	Adjusted R^2^	Model 1^a^		Model 2^b^	
							APR (95% CI)	p-value	APR (95% CI)	p-value
Population characteristics										
Population type										
General populations	134	65,212	1.00	–	<0.001	18.0	1.00	–	1.00	–
Intermediate-risk populations	4	947	4.54 (1.70–12.12)	0.003			4.36 (1.85–10.25)	0.001	4.31 (1.82–10.22)	0.001
Female sex workers	8	2163	1.33 (0.64–2.75)	0.45			2.07 (0.90–4.80)	0.088	1.81 (0.78–4.18)	0.17
Symptomatic women	208	42,737	2.41 (1.90–3.06)	<0.001			2.07 (1.66–2.58)	<0.001	2.10 (1.69–2.62)	<0.001
Symptomatic men	3	265	2.61 (0.82–8.28)	0.10			1.48 (0.53–4.12)	0.45	1.36 (0.48–3.81)	0.56
Infertility clinic attendees	5	478	0.90 (0.33–2.42)	0.83			0.38 (0.15–0.95)	0.039	0.37 (0.15–0.92)	0.033
Women with miscarriages or ectopic pregnancies	4	349	0.82 (0.23–2.95)	0.76			0.55 (0.17–1.77)	0.32	0.62 (0.19–2.00)	0.42
STI clinic attendees	3	1294	0.53 (0.17–1.70)	0.29			0.72 (0.26–2.01)	0.53	0.63 (0.23–1.76)	0.38
HIV-positive individuals and individuals in HIV-discordant couples	1	50	10.87 (1.58–74.62)	0.015			7.27 (1.36–38.76)	0.020	8.12 (1.51–43.78)	0.015
Other populations^c^	30	11,352	0.97 (0.63–1.47)	0.87			1.09 (0.74–1.60)	0.67	1.18 (0.81–1.74)	0.39
Age group										
<20 years	13	462	1.00	–	0.048	2.2	1.00	–	1.00	–
20–29 years	32	3837	1.19 (0.45–3.18)	0.73			1.85 (0.79–4.31)	0.16	1.75 (0.75–4.11)	0.19
30–39 years	30	3359	1.29 (0.48–3.47)	0.62			1.92 (0.82–4.50)	0.13	1.83 (0.78–4.29)	0.17
40–49 years	19	1607	0.82 (0.28–2.37)	0.71			1.29 (0.51–3.21)	0.59	1.24 (0.49–3.10)	0.65
≥50 years	8	2267	0.24 (0.06–0.99)	0.049			0.49 (0.14–1.68)	0.26	0.45 (0.13–1.57)	0.21
Mixed ages	298	113,315	0.85 (0.34–2.12)	0.73			1.17 (0.53–2.59)	0.70	1.09 (0.49–2.42)	0.83
Sex										
Women	389	123,729	1.00	–	0.80	0.0	–	–	–	–
Men	11	1118	1.09 (0.55–2.18)	0.80			–	–	–	–
MENA subregion^d^										
Fertile crescent	263	45,103	1.00	–	<0.001^e^	14.7	–	–	–	–
Horn of Africa	5	1600	3.24 (1.34–7.82)	0.009			–	–	–	–
Maghreb	8	4656	0.76 (0.36–1.64)	0.49			–	–	–	–
Gulf	2	2656	0.14 (0.03–0.59)	0.007						
Iran	107	66,784	0.45 (0.35–0.57)	<0.001			–	–	–	–
Pakistan and Afghanistan	15	4048	0.43 (0.24–0.78)	0.005			–	–	–	–
National income										
LIC and LMIC	117	28,367	1.00	–	<0.001	3.6	1.00	–	1.00	–
UMIC and HIC	283	96,480	0.64 (0.50–0.82)	<0.001			0.63 (0.51–0.79)	<0.001	0.62 (0.5–0.77)	<0.001
Study methodology characteristics										
Assay type										
NAAT/PCR	80	15,814	1.00	–	0.22	0.4	–	–	–	–
Culture	137	33,646	0.78 (0.57–1.08)	0.14			–	–	–	–
Wet mount	179	74,858	0.73 (0.54–0.99)	0.046			–	–	–	–
Rapid test	4	528	1.12 (0.37–3.39)	0.84			–	–	–	–
Sample size										
<200	165	11,918	1.00	–	<0.001	19.6	1.00	–	1.00	–
≥200	235	112,929	0.40 (0.32–0.49)	<0.001			0.48 (0.39–0.59)	<0.001	0.49 (0.39–0.60)	<0.001
Sampling method										
Probability based	32	9605	1.00	–	0.34	0.1	–	–	–	–
Non-probability based	368	115,242	1.23 (0.81–1.86)	0.34			–	–	–	–
Response rate										
≥80%	19	6620	1.00	–	0.046	1.3	1.00	–	1.00	–
<80%	5	146	4.62 (1.33–16.10)	0.016			6.53 (2.12–20.14)	0.001	6.96 (2.22–21.78)	0.001
Unclear	376	118,081	1.61 (0.90–2.87)	0.11			1.96 (1.06–3.61)	0.032	1.86 (0.99–3.47)	0.052
Temporal trend										
Year of data collection category										
<2000	40	11,574	1.00	–	0.16	0.5	1.00	–	–	–
2000–2009	145	63,430	0.73 (0.49–1.10)	0.14			0.68 (0.48–0.96)	0.028	–	–
≥2010	215	49,843	0.91 (0.61–1.34)	0.63			0.83 (0.59–1.16)	0.28	–	–
Year of data collection	400	124,847	1.00 (0.99–1.02)	0.84	0.84	0.0	–	–	0.99 (0.98–1.01)	0.44
Year of publication category										
<2005	41	11,172	1.00	–	0.30^f^	0.2	–	–	–	–
2005–2015	163	70,279	0.78 (0.52–1.16)	0.21			–	–	–	–
>2015	196	43,396	0.91 (0.61–1.35)	0.63			–	–	–	–
Year of publication	400	124,847	1.00 (0.98–1.01)	0.58	0.58^f^	0.0	–	–	–	–

The analyses incorporated national income (in place of MENA subregion) and the year of data collection as variables.

Abbreviations: APR = Adjusted prevalence ratio, CI = Confidence interval, HIC = High-income country, HIV = Human immunodeficiency virus, MENA = Middle East and North Africa, NAAT = Nucleic acid amplification test, LIC = Low-income country, LMIC = Low-middle-income country, LR test = Likelihood ratio test, PCR = Polymerase chain reaction, PR = Prevalence ratio, STI = Sexually transmitted infection, UMIC = Upper-middle-income country.

The PR represents the exponentiated beta coefficient calculated by the meta-regression model.

a Adjusted R^2^ in the final multivariable model 1 = 40.8%. Model 1 includes population type, age group, national income, sample size, response rate, and year of data collection as a categorical variable. Other variables were not included either because their p-values in the univariable model were greater than 0.2 or due to collinearity with another variable included in the model.

b Adjusted R^2^ in the final multivariable model 2 = 39.8%. Model 2 includes population type, age group, national income, sample size, response rate, and year of data collection as a continuous linear term. Other variables were not included either because their p-values in the univariable model were greater than 0.2 or due to collinearity with another variable included in the model.

c Other populations include populations with an undetermined risk of acquiring *Trichomonas vaginalis* such as women with premature labor, cancer patients, patients suffering from diabetes, and mixed at-risk populations, among others.

d Countries included in each MENA subregion are as follows: Fertile Crescent (Egypt, Iraq, Jordan, Lebanon, Palestine, Syria); Horn of Africa (Djibouti, Somalia, Sudan, Yemen); Maghreb (Algeria, Libya, Morocco, Tunisia); and Gulf (Bahrain, Kuwait, Oman, Qatar, Saudi Arabia, United Arab Emirates).

e MENA subregion was not included in the multivariable model due to collinearity with national income variable.

f Year of publication was not included in the multivariable model due to collinearity with year of data collection variable.

## Discussion

This study identified a considerable TV prevalence of 4.7% among the general population of the MENA region, with the vast majority of studies focusing on women. This prevalence level is comparable to the estimated global prevalence among women, which stands at 4.9%.^5^^,^^6^ This finding is unexpected for a region known for its conservative sexual norms, but aligns with the unexpectedly high prevalence of chlamydia^25^ and gonorrhea^41^ recently observed in this region. The findings shed light on a substantial yet often overlooked disease burden of curable STIs, which may have serious clinical, psychosocial, and economic implications, particularly in the absence of adequate sexual health and STI programs.^27^, ^28^, ^29^, ^30^, ^31^, ^32^

Despite the MENA region's limited response to STIs and the absence of specific programs targeting TV infection, a declining trend in TV prevalence has been observed. However, this decline is occurring at a slow rate of only 1% per calendar year, far below what is needed to meet the WHO's target of reducing TV incidence by 50% by 2030.^24^ The drivers of this decline are uncertain, but it is possible that this could be an indirect benefit of increasing HIV programs and some initiatives for improving STI management and control in certain countries in the region.^28^

While the findings are consistent with the presence of sexual networks where TV is being transmitted alongside other curable STIs, they may not strictly indicate substantial levels of risky sexual behaviors. Notably, the prevalence of incurable viral STIs in MENA tends to be lower than in other regions.^31^^,^^47^^,^^49^^,^^75^^,^^76^ The considerable TV prevalence may instead reflect inadequate access to, and utilization of, STI screening and treatment services, particularly since testing for this infection typically occurs only when symptoms are present, in the context of a lack of established and specific testing guidelines.^77^ Limited availability of diagnosis and specific treatment for curable STIs could contribute to unusually high prevalence levels, as has been observed in other regions.^78^, ^79^, ^80^ TV infection is often asymptomatic,^1^^,^^2^^,^^7^ and if left untreated, can persist for an extended duration, thereby increasing the potential for transmission within the population.

TV infection is associated with adverse reproductive health outcomes.^1^, ^2^, ^3^^,^^7^, ^8^, ^9^, ^10^, ^11^, ^12^^,^^14^ However, this study found no evidence indicating a higher TV prevalence among infertility clinic attendees and women with miscarriages or ectopic pregnancies as compared to the general population. This is in contrast to the evidence in MENA for higher prevalence of both chlamydia^25^ and gonorrhea.^41^ However, distinguishing the specific role of particular STIs or other factors in different reproductive outcomes remains a challenging task.^42^^,^^81^^,^^82^ This finding may have been also influenced by the relatively small number of studies identified for TV prevalence among these specific populations.

TV prevalence was notably high among symptomatic women but relatively low among STI clinic attendees. This observation aligns with the documented higher disease burden of this infection among women,^1^, ^2^, ^3^^,^^7^, ^8^, ^9^, ^10^, ^11^, ^12^^,^^83^ and may suggest a comparatively lower impact of this infection on urethritis in men. STI clinic attendees often consist of men presenting with urethral discharge, primarily attributed to gonorrhea.^84^, ^85^, ^86^ Interestingly, the pooled mean prevalence of gonorrhea among symptomatic men in MENA was high, at 43%.^41^

TV prevalence demonstrated an inverse relationship with national income, consistent with the global trend for TV prevalence.^5^^,^^6^^,^^87^ This may reflect better access to healthcare and greater awareness of STIs in higher-income countries. In contrast to findings from other regions,^5^^,^^6^^,^^87^ no significant difference in prevalence by sex was observed. However, the number of prevalence measures involving men was limited, which may have hindered the ability to discern a potential sex-based effect on prevalence.

TV prevalence exhibited a hierarchical pattern, with higher levels observed in higher-risk populations, such as female sex workers. This aligns with the typical pattern seen in the prevalence of other STIs.^21^^,^^25^^,^^28^^,^^37^^,^^47^^,^^88^ These findings underscore the need for targeted interventions in key populations to mitigate TV transmission and the associated disease burden.^21^^,^^25^^,^^28^ Studies using culture, wet mount, and rapid tests reported lower TV prevalence compared to those employing NAAT/PCR, suggesting a need for standardizing the use of NAAT/PCR in TV testing.

This study has limitations. The diagnostic methods for TV infection have well-documented limitations, which can introduce bias into observed TV prevalence.^1^^,^^53^, ^54^, ^55^ To mitigate this limitation, we developed standard inclusion criteria with guidance from an expert in laboratory methods for TV infection. Consequently, 99 publications reporting TV prevalence were excluded (Fig. 1) because they did not meet these criteria due to concerns regarding the reliability of the diagnostic methods employed.

Furthermore, even among studies meeting the standard inclusion criteria, a comprehensive assessment of the laboratory methods used in each included study was conducted, resulting in the establishment of highly stringent inclusion criteria. The study's analyses were presented using both the standard and stringent inclusion criteria. Both sets of analyses produced consistent findings.

The number of prevalence measures exhibited large variation among countries, with Iran and Iraq accounting for a large proportion of the publications on TV prevalence. Data were available for only 13 out of the 23 countries in MENA. The vast majority of studies focused on women, with a limited number of studies on men. Most studies reported TV prevalence in the general population and among symptomatic women, while only a small proportion of studies investigated prevalence among female sex workers, infertility clinic attendees, and women with miscarriages or ectopic pregnancies.

The number of studies in specific populations, such as intermediate-risk populations, STI clinic attendees, and HIV-positive individuals and individuals in HIV-discordant couples, was relatively limited. Therefore, the pooled estimates for these groups may underestimate the confidence intervals, as confirmed by the sensitivity analysis using the Hartung-Knapp-Sidik-Jonkman method.^66^, ^67^, ^68^ The small number of studies also potentially compromises the representativeness of estimates for these groups, results in wide confidence intervals for their associations, and introduces the possibility of sparse-data bias.^89^ Unlike studies for other STIs, such as gonorrhea and chlamydia,^45^ no study reported TV prevalence among contacts of individuals with TV.

TV prevalence in the population at large was approximated by the pooled estimate of all available prevalence measures for the general population. However, the estimation of prevalence in the whole MENA population would be more robust with the incorporation of probability weights for the different population strata and demographic and country categories. Heterogeneity in prevalence was evident across the studies, but approximately half of this heterogeneity was subsequently explained by epidemiological factors or study methods through meta-regression analyses.

Variations were noted in sample sizes and response rates among the studies. These factors were found to be associated with the reported prevalence, indicating potential methodological limitations in the available studies. A pronounced small-study effect^25^ was observed, with studies featuring a sample size of at least 200 reporting a lower prevalence by approximately 55%. This was confirmed by the analysis that demonstrated the presence of publication bias. Convenience sampling was the predominant method used in the available studies, as opposed to probability-based sampling methods.

In general, studies employing less robust methods tended to report higher TV prevalence, whereas studies of higher quality reported lower prevalence. There were instances of unusually high prevalence values reported, even in populations expected to have a low risk of infection, suggesting potential undisclosed biases in sample recruitment. These limitations suggest that the estimated pooled prevalence may potentially overestimate the true TV prevalence. This highlights the importance of enhancing study methods for investigating STIs in MENA.

This study has notable strengths. It employed a comprehensive search strategy with no time or language restrictions and conducted meticulous assessments of laboratory methods, extensive data extraction, and evaluations of the included studies' quality, potential sources of bias, and their impact on reported prevalence. The study also identified and analyzed a substantial volume of data, providing insights into TV epidemiology in MENA for the first time. The analytical approaches employed and the wide-ranging results have made meaningful contributions to our understanding of TV epidemiology across diverse populations and settings. Consequently, these findings hold practical implications for the development and expansion of STI and sexual health programs in this region.

In conclusion, despite sexually conservative norms, the MENA region exhibited a considerable prevalence of TV infection, on par with the global prevalence of this infection. The unexpectedly high prevalence for such a curable infection may, in part, be attributed to inadequate access to and utilization of STI screening and treatment services. While a declining trend in prevalence was observed, the rate of decline was sluggish, falling far short of the WHO's target to reduce TV incidence by 50% by 2030.^24^ Despite the study's advancement of our understanding of TV epidemiology in this region, several evidence gaps persist, including the absence of data from several MENA countries and limited data on specific populations. It is imperative for STI studies in MENA to bolster their quality, incorporate state-of-the-art methods in STI epidemiological research, and investigate other relevant aspects for TV infection, such as the availability of treatment, its effectiveness, and response to treatment in this region.

The observed TV epidemiology in MENA underscores the critical need for a tailored, comprehensive response specific to the region. Enhancing the current public health strategy with effective, gender-specific, and culturally appropriate programs is essential, alongside efforts to overcome STI stigma and implement key interventions. These interventions should include the integration of STI services with broader health programs and routine testing for key populations.

## Contributors

MH, WSG, AA, and AS conducted the systematic search, screened records, and performed data extraction and double extraction. MH conducted the data analyses. MH and LJA wrote the first draft of the manuscript. LJA conceived the study, oversaw the data extraction, analyses, and interpretation of the results. All authors contributed to discussions, interpretation of the results, and drafting and revising the manuscript. MH and LJA have accessed and verified the underlying data. All authors read and approved the final version of the manuscript.

## Data sharing statement

The data for this study were extracted from published literature. Pertinent data are presented in both the manuscript and its Supplementary Material file.

## Declaration of interests

The authors declare no competing interests.

## Appendix ASupplementary data

Supplementary Fig. S1, Box S1–S6, and Tables S1–S8
