# Supplementary material for: Epidemiology of Trichomonas vaginalis infection in the Middle East and North Africa: systematic review, meta-analyses, and meta-regressions
Source: eBioMedicine. 2024 Jul 17;106:105250. doi: 10.1016/j.ebiom.2024.105250 (PMC11286989; doi:10.1016/j.ebiom.2024.105250)
Supplement: Supplementary Fig. S1, Box S1–S6, and Tables S1–S8 [file mmc1.docx]

**Supplementary Material**

Table of Contents

[**Table S1.** Preferred Reporting Items for Systematic Reviews and Meta-analyses (PRISMA) checklist.^1,2^ 3](#_Toc166761639)

[**Table S2.** Data sources and terms of search strategies employed for the systematic review of *Trichomonas vaginalis* epidemiology in the Middle East and North Africa. 6](#_Toc166761640)

[**Box S1.** Countries encompassed within the Middle East and North Africa definition and their respective subregional categorizations. 7](#_Toc166761641)

[**Box S2.** Standard inclusion and exclusion criteria for *Trichomonas vaginalis* infection diagnostic methods. 8](#_Toc166761642)

[**Box S3.** Variables extracted from the publications that met the inclusion criteria. 9](#_Toc166761643)

[**Table S3.** Range of quality components applicable to prevalence studies and their applicability to this systematic review study.^3,4^ 10](#_Toc166761644)

[**Box S4.** Factors (variables) chosen *a priori* and integrated into both the univariable and multivariable meta-regression analyses in this study. 12](#_Toc166761645)

[**Box S5.** List of publications that satisfied the standard inclusion criteria and from which *Trichomonas vaginalis* prevalence measures were extracted. 13](#_Toc166761646)

[**Box S6.** List of publications that satisfied the stringent inclusion criteria and from which *Trichomonas vaginalis* prevalence measures were extracted. 20](#_Toc166761647)

[**Table S4.** Summary of precision and risk of bias assessments for studies reporting *Trichomonas vaginalis* prevalence in the Middle East and North Africa. 24](#_Toc166761648)

[**Table S5.** Assessment of publication bias using Doi plots and the LFK index. 25](#_Toc166761649)

[**Table S6.** Results of meta-analyses for *Trichomonas vaginalis* prevalence in the Middle East and North Africa, utilizing exclusively prevalence measures that met the stringent inclusion criteria for diagnostic methods. The results encompass diverse populations and are stratified by assay type. 26](#_Toc166761650)

[**Table S7.** Sensitivity analysis. Results of meta-analyses for *Trichomonas vaginalis* prevalence in the Middle East and North Africa using the Hartung-Knapp-Sidik-Jonkman method.^6-8^ 28](#_Toc166761651)

[**Figure S1.** Forest plots displaying the pooled mean *Trichomonas vaginalis* prevalence among diverse populations in the Middle East and North Africa. 30](#_Toc166761652)

[**Table S8.** Sensitivity analysis. Univariable and multivariable meta-regression analyses for *Trichomonas vaginalis* prevalence in the Middle East and North Africa, incorporating national income (in place of MENA subregion) and year of publication (in place of year of data collection) as variables. Prevalence measures conformed to the standard inclusion criteria for diagnostic methods. 45](#_Toc166761653)

[**References** 47](#_Toc166761654)

# **Table S1.** Preferred Reporting Items for Systematic Reviews and Meta-analyses (PRISMA) checklist.^1,2^

| **Section and topic** | **Item #** | **Checklist item** | **Location where item is reported** |
| --- | --- | --- | --- |
| **Title** | | |  |
| Title | 1 | Identify the report as a systematic review. | Title |
| **Abstract** | | |  |
| Abstract | 2 | See the PRISMA 2020 for Abstracts checklist (table 2). | Summary section |
| **Introduction** | | |  |
| Rationale | 3 | Describe the rationale for the review in the context of existing knowledge. | Introduction section |
| Objectives | 4 | Provide an explicit statement of the objective(s) or question(s) the review addresses. | Introduction section |
| **Methods** | | |  |
| Eligibility criteria | 5 | Specify the inclusion and exclusion criteria for the review and how studies were grouped for the syntheses. | Methods: Study selection process and inclusion, exclusion criteria; Box S2 |
| Information sources | 6 | Specify all databases, registers, websites, organisations, reference lists and other sources searched or consulted to identify studies. Specify the date when each source was last searched or consulted. | Methods: Data sources and search strategy; Table S2 |
| Search strategy | 7 | Present the full search strategies for all databases, registers and websites, including any filters and limits used. | Table S2 |
| Selection process | 8 | Specify the methods used to decide whether a study met the inclusion criteria of the review, including how many reviewers screened each record and each report retrieved, whether they worked independently, and if applicable, details of automation tools used in the process. | Methods: Study selection process and inclusion and exclusion criteria; Box S2 |
| Data collection process | 9 | Specify the methods used to collect data from reports, including how many reviewers collected data from each report, whether they worked independently, any processes for obtaining or confirming data from study investigators, and if applicable, details of automation tools used in the process. | Methods: Data extraction and data synthesis |
| Data items | 10a | List and define all outcomes for which data were sought. Specify whether all results that were compatible with each outcome domain in each study were sought (e.g., for all measures, time points, analyses), and if not, the methods used to decide which results to collect. | Methods: Data extraction and data synthesis; Boxes S3 and S4 |
|  | 10b | List and define all other variables for which data were sought (e.g., participant and intervention characteristics, funding sources). Describe any assumptions made about any missing or unclear information. | Boxes S3 and S4 |
| Study risk of bias assessment | 11 | Specify the methods used to assess risk of bias in the included studies, including details of the tool(s) used, how many reviewers assessed each study and whether they worked independently, and if applicable, details of automation tools used in the process. | Methods: Precision, risk of bias, and publication bias assessments; Table S3 |
| Effect measures | 12 | Specify for each outcome the effect measure(s) (e.g. risk ratio, mean difference) used in the synthesis or presentation of results. | Methods: Data extraction and data synthesis; Boxes S3 and S4 |
| Synthesis methods | 13a | Describe the processes used to decide which studies were eligible for each synthesis (e.g. tabulating the study intervention characteristics and comparing against the planned groups for each synthesis (item #5)). | Methods: Meta-analyses; Meta-regressions; Boxes S3 and S4 |
|  | 13b | Describe any methods required to prepare the data for presentation or synthesis, such as handling of missing summary statistics, or data conversions. | Methods: Meta-analyses; Meta-regressions; Boxes S3 and S4 |
|  | 13c | Describe any methods used to tabulate or visually display results of individual studies and syntheses. | Methods: Meta-analyses; Meta-regressions; Boxes S3 and S4 |
|  | 13d | Describe any methods used to synthesise results and provide a rationale for the choice(s). If meta-analysis was performed, describe the model(s), method(s) to identify the presence and extent of statistical heterogeneity, and software package(s) used. | Methods: Meta-analyses; Meta-regressions; Boxes S3 and S4 |
|  | 13e | Describe any methods used to explore possible causes of heterogeneity among study results (e.g. subgroup analysis, meta-regression). | Methods: Meta-analyses; Meta-regressions; Boxes S3 and S4 |
|  | 13f | Describe any sensitivity analyses conducted to assess robustness of the synthesised results. | Methods: Meta-analyses; Meta-regressions; Boxes S3 and S4 |
| Reporting bias assessment | 14 | Describe any methods used to assess risk of bias due to missing results in a synthesis (arising from reporting biases). | N/A |
| Certainty assessment | 15 | Describe any methods used to assess certainty (or confidence) in the body of evidence for an outcome. | N/A |
| **Results** | | |  |
| Study selection | 16a | Describe the results of the search and selection process, from the number of records identified in the search to the number of studies included in the review, ideally using a flow diagram (see fig 1). | Results: Search results and scope of evidence; Figure 1 |
|  | 16b | Cite studies that might appear to meet the inclusion criteria, but which were excluded, and explain why they were excluded. | Figure 1 |
| Study characteristics | 17 | Cite each included study and present its characteristics. | Results: Search results and scope of evidence; *Trichomonas vaginalis* prevalence overview; Table 1; Boxes S5 and S6 |
| Risk of bias in studies | 18 | Present assessments of risk of bias for each included study. | Results: Precision, risk of bias, and publication bias assessments; Tables S4 and S5 |
| Results of individual studies | 19 | For all outcomes, present, for each study: (a) summary statistics for each group (where appropriate) and (b) an effect estimate and its precision (e.g. confidence/credible interval), ideally using structured tables or plots. | Table 1; Figure S1 |
| Results of syntheses | 20a | For each synthesis, briefly summarise the characteristics and risk of bias among contributing studies. | Results: Precision, risk of bias, and publication bias assessments; Tables S4 and S5 |
|  | 20b | Present results of all statistical syntheses conducted. If meta-analysis was done, present for each the summary estimate and its precision (e.g. confidence/credible interval) and measures of statistical heterogeneity. If comparing groups, describe the direction of the effect. | Results: Pooled mean estimates of *Trichomonas vaginalis* prevalence; Table 1; Tables S6 and S7; Figure S1 |
|  | 20c | Present results of all investigations of possible causes of heterogeneity among study results. | Results: Predictors of prevalence and sources of between-study heterogeneity section; Tables 2-4; Table S8 |
|  | 20d | Present results of all sensitivity analyses conducted to assess the robustness of the synthesised results. | Results: Predictors of prevalence and sources of between-study heterogeneity section; Tables 2-4; Table S8 |
| Reporting biases | 21 | Present assessments of risk of bias due to missing results (arising from reporting biases) for each synthesis assessed. | N/A |
| Certainty of evidence | 22 | Present assessments of certainty (or confidence) in the body of evidence for each outcome assessed. | N/A |
| **Discussion** | | |  |
| Discussion | 23a | Provide a general interpretation of the results in the context of other evidence. | Discussion section |
|  | 23b | Discuss any limitations of the evidence included in the review. | Discussion section |
|  | 23c | Discuss any limitations of the review processes used. | Discussion section |
|  | 23d | Discuss implications of the results for practice, policy, and future research. | Discussion section |
| **Other information** | | |  |
| Registration and  protocol | 24a | Provide registration information for the review, including register name and registration number, or state that the review was not registered. | N/A |
|  | 24b | Indicate where the review protocol can be accessed, or state that a protocol was not prepared. | N/A |
|  | 24c | Describe and explain any amendments to information provided at registration or in the protocol. | N/A |
| Support | 25 | Describe sources of financial or non-financial support for the review, and the role of the funders or sponsors in the review. | Funding and Acknowledgements |
| Competing interests | 26 | Declare any competing interests of review authors. | Declaration of interests |
| Availability of data, code, and other materials | 27 | Report which of the following are publicly available and where they can be found: template data collection forms; data extracted from included studies; data used for all analyses; analytic code; any other materials used in the review. | Data sharing statement |

Abbreviations: NA = Not applicable, p = page.

# **Table S2.** Data sources and terms of search strategies employed for the systematic review of *Trichomonas vaginalis* epidemiology in the Middle East and North Africa.

| **PubMed (Last searched: March 1, 2024)** |
| --- |
| (Trichomonas Infections[Mesh] OR Trichomoniasis [Text] OR T.Vaginalis [Text] OR Trichom* [Text] Trichomonas Infections[Text] OR Trichomonas Vaginalis[Text] OR Trichomonas Vaginitis[Text] OR trichomonas OR trich*) AND "Middle East"[Mesh] OR "Islam"[Mesh] OR "Arabs"[Mesh] OR "Arab World"[Mesh] OR "Africa, Northern"[Mesh] OR "Sudan"[Mesh] OR "Somalia"[Mesh] OR "Djibouti"[Mesh] OR "Pakistan"[Mesh] OR “South Sudan”[Mesh] OR "Middle East*"[Text] OR "Middle-East"[Text] OR "North Africa*"[Text] OR "North-Africa"[Text] OR "EMRO"[Text] OR "Eastern Mediterranean"[Text] OR “Arab*”[Text] OR “Arab World”[Text] OR "Islam*"[Text] OR "Afghanistan"[Text] OR "Afghan*"[Text] OR "Algeria*"[Text] OR "Bahrain*"[Text] OR "Djibouti"[Text] OR "Egypt*"[Text] OR "Jordan*"[Text] OR "Kuwait*"[Text] OR "Lebanon"[Text] OR "Leban*"[Text] OR "Libya*"[Text] OR "Iran*"[Text] OR "Iraq*"[Text] OR "Morocco"[Text] OR “Moroccan*”[Text] OR "Oman*"[Text] OR "Pakistan*"[Text] OR "Qatar*"[Text] OR "Saudi*"[Text] OR "Somalia"[Text] OR "Somal*"[Text] OR "Sudan*"[Text] OR "Syria*"[Text] OR "Tunisia*"[Text] OR "United Arab Emirates"[Text] OR "Emirat*"[Text] OR "West Bank"[Text] OR "Ghaza*"[Text] OR "Gaza*"[Text] OR "Palestine"[Text] OR "Palestinian*"[Text] OR "Yemen*"[Text] OR “UAE”[Text] OR “KSA”[Text] OR "Dubai"[Text] OR "Abu Dhabi"[Text] OR "Abu-Dhabi"[Text] |
| **Embase (Last searched: March 1, 2024)** |
| exp Trichomonas/ or exp Trichomonas vaginalis/ or exp trichomoniasis/ or Trichomoniasis.mp. or Trichom*.mp. or Trichomonas Infections.mp. or Trichomonas Vaginalis.mp. or Trichomonas Vaginitis.mp. or trichomonas.mp. or trich*.mp. AND exp Middle East/ or exp North Africa/ or exp Arab/ or exp Afghanistan/ or exp Djibouti/ or exp Pakistan/ or exp Somalia/ or exp Sudan/ or exp South Sudan/ or (Middle East or North Africa or EMRO or Eastern Mediterranean or Arab or Arabs or Arab World or Islam or Afghanistan or Afghan* or Algeria* or Bahrain* or Djibouti or Egypt* or Jordan* or Kuwait* or Leban* or Libya* or Iran* or Iraq* or Morocc* or Oman* or Pakistan* or Qatar* or Saudi* or Somal* or Sudan* or Syria* or Tunisia* or United Arab Emirates or Emirat* or West Bank or Ghaza* or Gaza* or Palestin* or Yemen* or UAE or KSA or Dubai or Abu Dhabi or Sharjah).mp. |
| **Web of Science (Last searched: March 1, 2024)** |
| ALL=(Trichomonas)) OR ALL=(Trichomonas vaginalis)) OR ALL=(Trichomoniasis )) OR ALL=(T. Vaginalis )) OR ALL=(Trichomonas Infections) and AFGHANISTAN or ALGERIA or BAHRAIN or DJIBOUTI or EGYPT or IRAN or IRAQ or JORDAN or KUWAIT or LEBANON or LIBYA or MOROCCO or OMAN or PAKISTAN or QATAR or SAUDI ARABIA or SOMALIA or SUDAN or SYRIA or TUNISIA or U ARAB EMIRATES or WEST BANK AND GAZA or YEMEN (Countries/Regions) |
| **SCOPUS (Last searched: March 1, 2024)** |
| ( ALL ( trichomonas ) OR ALL ( trichomonas AND vaginalis ) OR ALL ( trichomoniasis ) OR ALL ( t. AND vaginalis ) OR ALL ( trichomonas AND infections ) ) AND ( LIMIT-TO ( AFFILCOUNTRY , "Egypt" ) OR LIMIT-TO ( AFFILCOUNTRY , "Saudi Arabia" ) OR LIMIT-TO ( AFFILCOUNTRY , "Iraq" ) OR LIMIT-TO ( AFFILCOUNTRY , "Pakistan" ) OR LIMIT-TO ( AFFILCOUNTRY , "Sudan" ) OR LIMIT-TO ( AFFILCOUNTRY , "Jordan" ) OR LIMIT-TO ( AFFILCOUNTRY , "Qatar" ) OR LIMIT-TO ( AFFILCOUNTRY , "Lebanon" ) OR LIMIT-TO ( AFFILCOUNTRY , "Algeria" ) OR LIMIT-TO ( AFFILCOUNTRY , "Oman" ) OR LIMIT-TO ( AFFILCOUNTRY , "Palestine" ) OR LIMIT-TO ( AFFILCOUNTRY , "Yemen" ) OR LIMIT-TO ( AFFILCOUNTRY , "Bahrain" ) OR LIMIT-TO ( AFFILCOUNTRY , "Afghanistan" ) OR LIMIT-TO ( AFFILCOUNTRY , "Libya" ) OR LIMIT-TO ( AFFILCOUNTRY , "Morocco" ) OR LIMIT-TO ( AFFILCOUNTRY , "Kuwait" ) OR LIMIT-TO ( AFFILCOUNTRY , "Syrian Arab Republic" ) OR LIMIT-TO ( AFFILCOUNTRY , "Djibouti" ) OR LIMIT-TO ( AFFILCOUNTRY , "Iran" ) OR LIMIT-TO ( AFFILCOUNTRY , "Somalia" ) OR LIMIT-TO ( AFFILCOUNTRY , "Tunisia" ) OR LIMIT-TO ( AFFILCOUNTRY , "United Arab Emirates" ) ) |
| **National and regional databases (Last searched: March 1, 2024)** |
| 1. **Index Medicus for the Eastern Mediterranean Region**   Trichomonas or Trichomonas vaginalis or Trichomoniasis   1. **Iraqi Academic Scientific Journals Database**   Trichomonas or Trichomonas vaginalis or Trichomoniasis   1. **Scientific Information Database of Iran**   Trichomonas or Trichomonas vaginalis or Trichomoniasis   1. **PakMediNet of Pakistan**   Trichomonas or Trichomonas vaginalis or Trichomoniasis |

Abbreviations: MENA = Middle East and North Africa.

# **Box S1.** Countries encompassed within the Middle East and North Africa definition and their respective subregional categorizations.

- **Fertile Crescent:** Egypt, Iraq, Jordan, Lebanon, Palestine, Syria.
- **Gulf:** Bahrain, Kuwait, Oman, Qatar, Saudi Arabia, United Arab Emirates (UAE).
- **Horn of Africa:** Djibouti, Somalia, Sudan, Yemen.
- **Maghreb:** Algeria, Libya, Morocco, Tunisia.
- Afghanistan.
- Iran.
- Pakistan.

**Box S2.** Standard inclusion and exclusion criteria for *Trichomonas vaginalis* infection diagnostic methods.

**Studies are included except for:**

**Studies among women and men**

- Studies using staining techniques to test for TV.
- Studies using a latex agglutination assay to test for TV.
- Studies using an enzyme-linked immunosorbent assay to test for TV.
- Studies testing blood specimens for TV immunoglobulins.
- Studies with unclear testing protocols.

**Studies among women**

- Studies testing pap smears for TV.

**Studies among men**

- Studies testing semen specimens for TV.
- Studies testing urethral specimens for TV.
- Studies testing urine specimens for TV using culture.
- Studies testing urine specimens for TV using wet mount.

Abbreviations: TV = *Trichomonas vaginalis.*

**Box S3.** Variables extracted from the publications that met the inclusion criteria.

- Author(s).
- Year of publication.
- Full citation.
- Country.
- City.
- Study design.
- Sampling methodology.
- Year(s) of data collection.
- Study site.
- Study population.
- Population characteristics (e.g., sex and age).
- Response rate.
- Sample size of tested population.
- Number of participants testing positive for *Trichomonas vaginalis* infection.
- Reported *Trichomonas vaginalis* infection prevalence.
- Type of assay used for *Trichomonas vaginalis* infection ascertainment (nucleic acid amplification test/polymerase. chain reaction, culture, wet mount, and rapid test).

**Table S3.** Range of quality components applicable to prevalence studies and their applicability to this systematic review study.^3,4^

| **Risk of bias tool from Hoy, 2012^3^** | **Risk of bias tool from Munn, 2015^4^** | **Risk of bias assessment of included studies** |
| --- | --- | --- |
| Was the study's target population a close representation of the national population in relation to relevant variables? | Were study participants sampled in an appropriate way? | Met in the study design. The systematic review investigated prevalence in all population groups. The meta-regression analyses explored the impact of population type on observed prevalence. The sampling method was one of the investigated risk of bias domains. |
| Was the sampling frame a true or close representation of the target population? | Was the sample frame appropriate to address the target population? | Met in the study design. Included as the probability-based vs. non-probability-based sampling risk of bias domain. The meta-regression analyses also explored the impact of sampling method on observed prevalence. |
| Was some form of random selection used to select the sample, OR was a census undertaken? |  | Met in the study design. Included as the probability-based vs. non-probability-based sampling risk of bias domain. The meta-regression analyses also explored the impact of sampling method on observed prevalence. |
| Was the likelihood of nonresponse bias minimal? | Was the response rate adequate, and if not, was the low response rate managed appropriately? | Met in the study design. Included as the response rate risk of bias domain. The meta-regression analyses also explored the impact of response rate on observed prevalence. |
| Were data collected directly from the subjects (as opposed to a proxy)? |  | Met in the study design. The inclusion criteria specified that only studies based on biomarkers collected directly from individuals are included in this systematic review. |
| Was an acceptable case definition used in the study? | Were valid methods used for the identification of the condition? | Met in the study design. A standardized and consistent case definition was used, that of *Trichomonas vaginalis* infection diagnosed through laboratory methods. |
| Was the study instrument that measured the parameter of interest shown to have validity and reliability? | Was the condition measured in a standard, reliable way for all participants? | Met in the study design. Only studies that used diagnostic assays measuring specific biomarkers were included. Evaluation of the validity and reliability of the instruments used to measure prevalence was fulfilled through both the standardized and rigorous sets of inclusion and exclusion criteria for the laboratory methods described in the main text. The reported assays have acceptable specificity and sensitivity and are commonly used in research and clinical settings. The meta-regression analyses also explored the impact of assay type on observed prevalence. |
| Was the same mode of data collection used for all subjects? |  | Met in the study design. It is standard for STI studies involving biomarkers, by design, to use a consistent mode of data collection from all subjects recruited for a study, including specimen type and assay type. |
| Was the length of the shortest prevalence period for the parameter of interest appropriate? |  | Met in the study design. Included studies reported point prevalence measures, that is, prevalence based on a cross-sectional survey at a specific and appropriately relevant time interval. |
| Were the numerator(s) and denominator(s) for the parameter of interest appropriate? | Was there appropriate statistical analysis? | Met in the study design. The numerator and denominator were defined with no ambiguity: number of positive *Trichomonas vaginalis* cases over total number of tested subjects. |
|  | Was the data analysis conducted with sufficient coverage of the identified sample? | Met in the study design. Data analysis was done on the full reported and tested sample. |
|  | Was the sample size adequate? | Met in the study design. Included as the precision assessment of the sample size. The meta-regression analyses also explored the impact of sample size on observed prevalence. |
|  | Were the study subjects and the setting described in detail? | Met in the study design. For all included studies, the population type of study subjects and the study site were available and extracted. Populations were classified according to this systematic review definitions of study populations. |

# **Box S4.** Factors (variables) chosen *a priori* and integrated into both the univariable and multivariable meta-regression analyses in this study.

| 1. Population type as defined in Box S3 2. Sex 3. Age groups classified to best fit reported data as:  - <20 years old - 20-29 years old - 30-39 years old - 40-49 years old - ≥50 years old - Mixed age bands  1. MENA subregion as defined in Box S1 2. National income as classified by the World Bank^5^ 3. Assay type:  - NAAT/PCR - Culture - Wet mount - Rapid test  1. Sample size:  - <200 - ≥200  1. Sampling method:  - Probability-based sampling^a^ - Non-probability-based sampling^b^  1. Response rate:  - ≥80% - <80% - Unclear  1. Year of publication category:  - <2005 - 2005-2014 - ≥2015  1. Year of publication as a linear term 2. Year of data collection category^c^  - <2000 - 2000-2009 - ≥2010  1. Year of data collection as a linear term |
| --- |

Abbreviations: MENA = Middle east and North Africa, NAAT = Nucleic acid amplification test, PCR = Polymerase chain reaction.

^a^ Probability based sampling includes cluster random sampling, random sampling, respondent driven sampling, and stratified random sampling.

^b^ Non-probability-based sampling includes convenience sampling and snowball sampling.

^c^ The categories were determined based on the median time observed between the year of publication and the year of data collection, which was approximately 3 years. To create distinct brackets, this interval was approximated to 5 years, to have 5-year intervals.

# **Box S5.** List of publications that satisfied the standard inclusion criteria and from which *Trichomonas vaginalis* prevalence measures were extracted.

| 1. Abbas SK. دراﺳﺔ ﻛﯿﻤﯿﺎﺋﯿﺔ ﺣﯿﺎﺗﯿﺔ وﻣﻨﺎﻋﯿﺔ ﻟﻄﻔﯿﻠﻲ اﻟﻤﺸﻌﺮات اﻟﻤﮭﺒﻠﯿﺔ Trichomonas Vaginalis ﻓﻲ ﻣﺪﯾﻨﺔ ﺑﻐﺪاد. Al-Mustansiriyah Journal of Science 2010; 21(5): 230-40.  2. Abd el Ghaffar FM, Azab ME, Salem SA, Habib KS, Maklad KM, Habib FS. Evaluation of two cultural media (CPLM & TYM) for isolation and maintenance of Trichomonas vaginalis stocks in the laboratory. J Egypt Soc Parasitol 1994; 24(3): 611-9.  3. Abdel-Magied AA, El-Kholya EI, Abou El-Khair SM, et al. The genetic diversity of metronidazole susceptibility in Trichomonas vaginalis clinical isolates in an Egyptian population. Parasitol Res 2017; 116(11): 3125-30.  4. Abdelaziz ZA, Ibrahim ME, Bilal NE, Hamid ME. Vaginal infections among pregnant women at Omdurman Maternity Hospital in Khartoum, Sudan. J Infect Dev Ctries 2014; 8(4): 490-7.  5. Abdelrahim NA, Ahmed HI, Fadl-Elmula IM, Bayoumi MA, Homeida MM. Sexually transmitted infections other than HIV/AIDS among women of low socio-economic class attending antenatal clinics in Khartoum, Sudan. Int J STD AIDS 2017; 28(8): 781-7.  6. Abdu Elrab A, Al Wazer I, Al Duminy A, Al Awady H, Al Shamy H. Primary report on sexually transmitted diseases among women in Sana'a Yemen during 2003-2004. YHMRJ-Yemeni Health and Medical Research Journal 2004.  7. Abdul Jabbar ZR, Al-Warid HS. Some Clinical and Inflammatory Aspects of Trichomonas vaginalis Infection among Women with Pelvic Inflammatory Diseases. Iraqi Journal of Science 2021; 62(12): 4649-66.  8. Abdul Wehab Wajeh S, Abdul Hussein B, Abdul Razzaq M. Effect of sex hormones level on the external genital tract infection in pregnant and non- pregnant women in Dhi Qar governorate, Iraq. Journal of thi-qar science 2018; 6(4): 9-17.  9. Abdulkhudher NA, Yousif MG, DSadiq AM. Detection of immunological markers for Chlamydia trachomatis and Trichomonas vaginalis infection in women with obstetric complications in Najaf, Iraq التحري عن الدلائل المناعية لاصابات بكتريا trachomatis Chlamydia والمشعرات المهبلية vaginalis Trichomonas لدى النساء اللواتي لديهن مضعاعفات الحمل في محافظة النجف , العراق. Al-Qadisiyah Journal of Pure Science 2014; 19(2): 14-25.  10. Abdullah N, Abbas G, Mahmood K. Frequency of Common Pathogens in Vaginal Discharge. Age (Years) 2015; 13: 20.  11. Abdulwahab EG, Al Alousi TI, Yassin EM. Study on The prevalence of trichomoniasis in married women intended Tikrit teaching hospital دراسة تفشي داء المشعرات المهبلية trichomoniasis في النساء المتزوجات المراجعات لمستشفى تكريت التعليمي. Tikrit Journal of Pure Science 2011; 16(1): 143-6.  12. Aboulghar M, Aboushady O, Ahmed J, Hanafy N. Diagnosis of trichomonas vaginalis infection in women of childbearing age at a university setting using osom a new diagnostic technique. Egyptian Journal of Medical Microbiology, 2009; 18(9): 51-6.  13. Abu Shaqra QM. Bacterial vaginosis among a group of married Jordanian women: occurrence and laboratory diagnosis. Cytobios 2001; 105(408): 35-43.  14. Afzal M, Yasin S, Sadaf A, Umer S, Roman S, Irshad F. Vaginal Inflammation due to overgrowth of naturally occurring flora in healthy and sexually active women of reproductive age: A cross sectional study. Pakistan Journal of Medical & Health Sciences 2022; 16(1): 200-2.  15. Ahady MT, Safavi N, Jafari A, Mohammadi Z, Abed S, Pourasgar S. Prevalence of trichomoniasis among 18-48 year-old women in northwest of Iran. Iranian Journal of Parasitology 2016; 11(4): 580-4.  16. Ahmadi A, Farhadifar F, Rezaii M, et al. Group B streptococci and trichomonas vaginalis infections in pregnant women and those with spontaneous abortion at Sanandaj, Iran. Iranian Journal of Microbiology 2018; 10(3): 166-70.  17. Akbari Z, Matini M. The study of trichomoniasis in pregnant women attending Hamadan city health centers in 2015. Avicenna journal of clinical microbiology and infection 2017; 4(2): 0-.  18. Akbarian AR, Akhlaghi L, Ourmazdi H, Foroohesh H, Falahati M, Farokhnejad R. An Investigation on Coincidence of Trichomoniasis and Bacterial Vaginosis and their Effects on Pregnant Women Referred to Shahid Akbarabadi Maternity Hospital in Tehran During 2002-2003. RJMS 2005; 12(46): 227-34.  19. Akhlaghi L, Falahati M, Jahani Abianeh M, Ourmazdi H, Amini M. Study on the prevalence of trichomonas vaginalis and candida albicans in women referred to Robat Karim medical center and a comparative evaluation of loffler and diluted carbol fuchsin stains for rapid diagnosis of them. Razi journal of medical sciences (Journal of Iran university of medical sciences) 2005; 12(48 (SPECIAL ISSUE)): 75-12.  20. Al Gazali BS, Al-Hadraawy MKA, Al-fatlway HAA. Study of blood parameter in women infected with Trichomonas vaginalis parasite دراسة معاير الدم في النساء المصابات بطفيلي المشعرات المهبلية. journal of kerbala university 2013; 11(4): 223-7.  21. Al Kaisi AAR, Al Janabi B, Al Tikriti R. A study on the common microorganisms causing vaginitis. Iraqi Postgrad Med J 2006; 5(4): 426-30.  22. Al Mallah O, Al Janabi BM. The incidence of trichomonas vaginalis among selected groups of women in Mosul. IMJ-Iraqi Medical Journal 1983; 31 (): 29-33.  23. AL Muathen DM, Sachit HG. Prevalence Trichomoniasis and Candidiasis For Symptomatic Pregnant and Non Pregnant Women in Iraq دراسة انتشار داء المشعرات المهبلية وداء المبيضات لدى النساء الحوامل وغير الحوامل العرضيات في العراق. Al-Mustansiriyah Journal for Pharmaceutical Sciences 2016; 16(2): 21-6.  24. Al Quaiz JM. Patients with vaginal discharge: A survey in a university primary care clinic in Riyadh city. Annals of Saudi Medicine 2000; 20(3-4): 302-6.  25. Al- Mamoori ZZM, Alhameedawi JJ, Abid Alhisnawi AA. Molecular diagnosis of the toll-like receptor 4 genes and its relationship with the levels of some immunoglobulin’s and cytokines among infected women with trichomoniasis. J Global Pharma Technol 2018; 10(10): 196-202.  26. Al-Abady FAفع, العبادي, Al-Khazrajee ZA. Epidemiology Study Parasite Trichomonas Vaginalis at Thi-Qar Province دراسة وبائية لطفيلي المشعرة المهبلية Trichomonas vaginalis في محافظة ذي قار. Univesity of Thi-Qar Journal 2014; 9(1): 1-9.  27. Al-Abodi H, Al-Shaibani K, Shaker E. Molecular investigation of trichomoniasis in women in Al-Muthana province/Iraq. Journal of Physics: Conference Series; 2019: IOP Publishing; 2019. p. 012078.  28. Al-Ammash MSJA-A. Study on prevalence of Trichomomas vaginalis in Samarra city with observation of alcoholic extract effect of Peganum harmala plant in vitro دراسة وبائية لطفيلي المشعرات المهبلية Trichomomas vaginalis في مدينة سامراء مع ملاحظة تحضير المستخلص الكحولي لنبات الحرمل Peganum harmalaفي الزجاج. Tikret Journal of Pharmaceutical Sciences 2017; 12(1): 31-47.  29. Al-Ani MM, Al-Hadi A-HA-R, Faiza A. The role of cervical screening in early detection of cervical lesionsدور تقصي لطاخات عنق الرحم في التشخيص المبكر للافات. Iraqi journal of medical sciences 2005; 4(2): 141-7.  30. Al-Awadhi R, Al-Shaheen A, Al-Juwaiser A, George SS, Sharma P, Kapila K. Prevalence of Infectious Organisms Observed in Cervical Smears Between 1997-2014 at Mubarak Al-Kabeer Hospital, Kuwait. Sultan Qaboos Univ Med J 2018; 18(3): e324-e8.  31. Al-Falahi AH. Incidence of trichomonus vaginalis in different groups of women. Journal of Techniques 2007; 20(1): 85-8.  32. Al-Habib HM, Al-Dabbagh NY, Al-Daheen GA. The prevalence of trichomonas vaginalis in association with other micro-organisms among women with vaginal discharge in Mosul. Annals of the College of Medicine Mosul 2005; 31(1): 37-44.  33. Al-Hadraawy Sk, Al-Kafagy SMM, Al-Hadraawy M, K. A. Molecular and immunological study for detection of IL-6 in men infected with Trichomonas vaginalis parasite in Al-Najaf province; Iraq. Al-Qadisiyah Medical Journal 2014; 10(18): 144-8.  34. Al-Hadraawy SK, Al-Katib S, R. , Al-Awady H, M. H. Identification study for suspected women with Trichomonas vaginalis by PCR technique in AL-Najaf AL-Ashraf province دراسة تعريفية للاشخاص المشتبه في اصابتهم بالمهبلات المشعرة بتقنية PCR في محافظة النجف الاشرف. Al-Kufa University Journal for Biology 2018a; 10(1): 183-92.  35. Al-Hadraawy SK, Al-Katib S, R. , Al-Awady H, M. H. Immunological study for infected women with T.vaginalis in AL-Najaf AL-Ashraf province دراسة مناعية للنساء المصابات بمرض T.vaginalis في محافظة النجف الأشرف. Al-Kufa University Journal for Biology 2018b; 10(1): 132-7.  36. Al-Haidari DaMH. Detection of Trichomoniasis in Vaginal and Urine Specimens from Women in Karbala city تشخيص طفيلي المشعرات المهبلية في عينات بولية ومسحات مهبلية من النساء في مدينة كربلاء. Al-Qadisiyah Journal of Pure Science 2011; 16(1): 11-6.  37. Al-Harbi NY, R Shh, Jabuk SIA. The frequency of trichmonas vaginalis, candida albicanand bacterial vaginosis in vaginal smear from women oreproductive age. Indian Journal of Public Health Research and Development 2018; 9(8): 1411-5.  38. Al-Hindi AI, Lubbad AMH. Trichonamas vaginalis infection among Palestinian women: Prevalence and trends during 2000-2006. Turkish Journal of Medical Sciences 2006; 36(6): 371-5.  39. Al-Hussuny EM. An epidemiological study of Trichomonas vaginalis in among women living in Baquba City, Diyala Province, Iraq دراسة وبائيةُ لطفيلي Trichomonas vaginalis في نساء مدينة بعقوبة ، محافظة ديالى ، العراق. Diyala Journal For Pure Science 2015; 11(3): 13-25.  40. Al-Jammaly MM, Abdulla BA. Isolation and Identifcation of Micro–Organismٍٍs in Cervicitis and Vaginitis From Women in Mosul City عزل وتشخيص الأحياء المجهرية من التهاب عنق الرحم والمهبل في نساء مدينة الموصل. Rafidain journal of science 2008; 19(3 A): 20-31.  41. Al-Joubori SF. Lower Genito Urinary Infection in Gynecological Practice.in Baghdad Area التهابات المسالك الداخلية الجنسية والبولية لدى المرضى المراجعين لعيادات الأمراض النسائية. Iraqi Journal of Community Medicine 2005; 18(2): 165-72.  42. Al-Khawaty K, Al-Khafji ZM, Al-Shaykhy AS. Identification of trichomonas vaginalis using molecular methods in Iraqi infected womenتشخیص طفیلي في النساء العراقیات المصابات به بالطرق الجزیئیة Trichomonas vaginalis. Iraqi Journal of Biotechnology 2012; 11(1): 51-64.  43. Al-Marsomy HD. Association between trichomonas vaginalis and vaginal bacterial community composition in human vagina. Research Journal of Pharmacy and Technology 2020; 13(6): 2925-31.  44. Al-Masoudi HK. Polymerase chain reaction compared with wet mount for detection of Trichomonas vaginalis in women. Int J Pharm Res 2016; 9(3): 240-4.  45. Al-Mizury KS. The Effect of Using Contraceptives in Producing Trichomonas Vaginalis Infection in Women. Al-Mustansiriyah Journal of Science 2010; 21(1): 39-42.  46. Al-Mousawi JKN, Hashim RT, Manal MKA-Sدجادهطومكا. Microbiological study of bacterial vaginosis among pregnant women in Al-Diwaniya city. The Medical Journal of Basrah University 2006; 24(1&amp;2): 45-9.  47. Al-Muk JM, Hasony HJ. Isolation of Gardnerella vaginalis from pregnant women with bacterial vaginosis in Basrah, Iraq. Bahrain Med Bull 2001; 23(3): 124-6.  48. Al-Muqdadi SF, Mhaisen FT, Al-Tae AA. Distribution of the Infection with Trichomonas vaginalis and Associated Microorganisms in Women Attending Two Hospitals in Al-Sader City, Baghdad. Ibn Al-Haitham Journal For Pure and Applied Sciences مجلة ابن الهيثم للعلوم الصرفة والتطبيقية 2010; 23(1): 19-25.  49. Al-Omar SL, Al-Kaissi NE, Abdulla FS. Occurrence of HSV with other microorganisms in female genital infection. Al-Mustansiriyah Journal for Pharmaceutical Sciences 2005; 2(1): 1-6.  50. Al-Qurashi AM. Examination of urine for bacteria and parasites among elder persons in the rural area, in Dammam District. J Egypt Soc Parasitol 2006; 36(1): 297-304.  51. Al-Saeed WM. Detection of Trichomonas vaginalis by different methods in women from Dohok province, Iraq. East Mediterr Health J 2011; 17(9): 706-9.  52. Al-Tikrity IAA, Al-Badry MSM. The Trichomonasis Spread Between Married Women Revisions to The Health Center in Samarra انتشار داء المشعرات المهبليةTrichomoniasis بين النساء المتزوجات المراجعات للمركز الصحي في قضاء سامراء. kerbala journal of pharmaceutical sciences 2014; (7): 271-6.  53. Al-Zuharri OAR, Offi SY, Al-Jubory HTA-k. Genital tract infections among pregnant women التهابات القناة التناسلية بين النساء الحوامل. Al-Kufa University Journal for Biology 2010; 2(1): 207-12.  54. Ali MK, Hathal HD, Almoayed HA. Prevalence and diagnosis of sexually transmitted pathogens in a sample of Iraqi women: a molecular study. Iraqi journal of medical sciences 2017; 15(4): 364-76.  55. Alikhani M, Akhoundi M, Sereno D, et al. Molecular characterization of Trichomonas infections in women of Ilam City, southwestern Iran. Parasitol Res 2022; 121(6): 1631-8.  56. Aloui D, Trabelsi S, Bouchekoua M, Khaled S. [Vulvovaginal trichomoniasis: epidemiology, clinical and parasitological characteristics]. Tunis Med 2015; 93(6): 376-80.  57. Amina H, Bahija B, Sanae J. Prevalence of bacterial vaginosis in women presenting recurrent vaginal discharge in Morocco. Sexually Transmitted Infections 2017; 93(Supplement 2): A127-A8.  58. Anwer A, Sultana N. Prevalance of Sexually Transmitted Diseases among a selected group of Pakistani Women. Med Channel 2001; 7(4): 19-21.  59. Arbabi M, Fakhrieh Z, Delavari M, Abdoli A. Prevalence of Trichomonas vaginalis infection in Kashan city, Iran (2012-2013). Iranian Journal of Reproductive Medicine 2014; 12(7): 507-12.  60. Asifa Matroud yassin عاصفة مطرود ي. Study the infection of Trichomonas vaginalis for women In Al-diwanya دراسة حول الإصابة بطفيلي Trichomonas vaginalis لدى النساء في الديوانية. Al-Qadisiyah Journal of Pure Science 2011; 16(1): 72-8.  61. Aslam M, Hafeez R, Aman S, Azhar A, Lone DS. Bacterial vaginosis in Pregnant Women: A diagnostic approach. Ann King Edward Med Uni 2004; 10(1): 30-2.  62. Azadi Y, Didarloo H, Ardibazar M, et al. The prevalence of trichomonas vaginalis among pregnant women referred to the tabriz valiasr hospital, Tabriz, Iran. Iranian Journal of Parasitology 2017; 13(1): 58.  63. Azambakhtiar A, Nikmanesh B, Rezaeian M, Dashti N, Safari F, Zarebavani M. The prevalence of trichomoniasis in women referred to clinical centers in south of Tehran, Iran during 2015-2016. Iranian Journal of Parasitology 2018; 13(1): 108-13.  64. Azargoon A, Darvishzadeh S. Association of bacterial vaginosis, trichomonas vaginalis, and vaginal acidity with outcome of pregnancy. Arch Iran Med 2006; 9(3): 213-7.  65. Badparva E, Ali Papi O, Kheirandish F, Pornia Y, Azizi M. Sensitivity assessment of direct method for diagnosis of trichomonas vaginalis in comparison with Dorset culture media. Yafteh 2010; 12(1 (43)): 25-30.  66. Bafghi AF, Aflatoonian A, Barzegar K, Ghafourzadeh M, Nabipour S. Frequency distribution of trichomoniasis in pregnant women referred to health centers of Ardakan, Meibod and Yazd, Iran. Jundishapur Journal of Microbiology 2009; 2(4): 132-9.  67. Baghaei M, Memarzadeh Z. Prevalence of trichomoniasis in women: Isfahan 1995. Journal Of Research In Medical Sciences (JRMS) 2001; 6(Supplement 2): 108-12.  68. Bahram A, Hamid B, Zohre T. Prevalence of bacterial vaginosis and impact of genital hygiene practices in non-pregnant women in Zanjan, Iran. Oman Medical Journal 2009; 24(4): 288-93.  69. Bahreini MS, Sedghi S, Badalzadeh Y, et al. Molecular diagnosis of Trichomonas vaginalis in liquid-based Papanicolaou samples in Shiraz, southern Iran. BMC Womens Health 2023; 23(1): 6.  70. Bakhshandehnosrat S, Ghaemi E, BEHNAMPOUR N, Rezayayi M. Determining the etiological agents in vaginal infections in women referring to Dezyani Women Hospital in Gorgan. J Sabzevar Univ Med Sci 2003; 10(3): 58-65.  71. Bakhshi A, Safayi Delouyi Z, Taheri S, Alivandi A, Mohammadzadeh N, Dabiri H. Comparative study of lactobacilli and bifidobacteria in vaginal tract of individual with bacterial vaginosis and healthy control by quantitative PCR. Reviews in Medical Microbiology 2019; 30(3): 148-54.  72. Bakhtiari A, Hajian-Tilaki K, Pasha H. Genital infection by Trichomonas Vaginalis in women referring to Babol health centers: Prevalence and risk factors. Iranian Red Crescent Medical Journal 2008; 10(1): 16-21.  73. Banno IS, Nakkash AF, Mizi’l SN. Isolation and Idintification of candida albicans from vagina and Study of some Virulance Factors عزل وتشخيص المبيضات البيضاء Candida albicans من المهبل ودراسة بعض عوامل ضراوتها. Baghdad Science Journal 2010; 7(عدد خاص بمؤتمر العلمي النسوي 1): 233-40.  74. Beal C, Goldsmith R, Kotby M, et al. The plastic envelope method, a simplified technique for culture diagnosis of trichomoniasis. J Clin Microbiol 1992; 30(9): 2265-8.  75. Bellaji B, Hancali A, Jennane S, et al. Prevalence of chlamydia trachomatis, neisseria gonorrhoeae and trichomonas vaginalis in female sex workers in morocco. Sexually Transmitted Infections 2017; 93(Supplement 2): A100.  76. Bhatti NR, Hayat A, Ahmad S, et al. Prevalance of Vaginal Candiasis in Gynaecology. Pak J Obstet Gynaecol 1995; 8(3): 11-5.  77. Bokharaei-Salim F, Esteghamati A, Khanaliha K, Esghaei M, Donyavi T, Salemi B. The first detection of co-infection of double-stranded rna virus 1, 2 and 3 in iranian isolates of trichomonas vaginalis. Iranian Journal of Parasitology 2020; 15(3): 357-63.  78. Bokharaei-Salim F, Hedayati N, Khanaliha K, et al. Molecular typing of the actin gene of Trichomonas vaginalis isolates in Tehran, Iran. Journal of Parasitic Diseases 2022.  79. Bolbol Haghighi N, Ebrahimi H, Nourouzi P, Delvarianzadeh M. evaluation and comparison of clinical and para-clinical diagnosis of trichomonas vaginitis in women referred to Shahroud city health care centers. Knowledge and Health 2008; 3(1): 33-8.  80. Boulos LM, El Temsahy MM, Aly SM, El Agamy ESI, Amer EI. Biological and biochemical studies for characterization of some Egyptian Trichomonas vaginalis isolates. PUJ-Parasitologists United Journal 2012; 5(2): 175-88.  81. Chalechale A, Karimi I. The prevalence of Trichomonas vaginalis infection among patients that presented to hospitals in the Kermanshah district of Iran in 2006 and 2007. Turkish Journal of Medical Sciences 2010; 40(6): 971-5.  82. Chaloob FA, Abdul-Mohsen AS. Association of Toll-Like Receptor 4 Gene Oolymorphism with Trichomonas vaginalis Infection in Iraqi Women العلاقة بين تعدد الاشكال الجينية لجين Toll-like receptor 4 مع الاصابة بطفيلي المشعرات المهبلية في النساء العراقيات. Medical Journal of Babylon 2014; 11(1): 84-91.  83. Chaudry AE, Chaudhri R, Kayani A, et al. Acceptability and feasibility of screening pregnant women for sexually transmitted infections in Rawalpindi, Pakistan. Int J STD AIDS 2021; 32(10): 940-5.  84. Dalimi A, Payameni S. Trichomonas vaginalis infection in men with high-risk sexual behaviors. Iranian Journal of Parasitology 2021; 16(3): 411-7.  85. Dawood IS, Kadir MA, Sulyman MA. Epidemiological study of infection with Trichomonas vaginalis in Kirkuk city وبائية الإصابة بالمشعَرات المهبلية في مدينة كركوك. Tikrit Journal of Pure Science 2013; 18(1): 48-55.  86. Dhumad Hameedi H, Al-Shammari ZAD. Aerobic Bacterial Vaginosis and Lactobacillus Species Associated with Cytomegalovirus in Abortion. Arch Razi Inst 2022; 77(4): 1447-52.  87. Dyab AK, Farouk HA, Mohammed MF, Hassan TM. Parasitological Studies on Trichomonus Vaginalis on Female Patients Presented with Vaginal Discharge at Aswan University Hospital. The Medical Journal of Cairo University 2021; 89(June): 1147-54.  88. El Beayni N, Hamad L, Nakad C, Keleshian S, Yazbek SN, Mahfouz R. Molecular prevalence of eight different sexually transmitted infections in a lebanese major tertiary care center: Impact on public health. International Journal of Molecular Epidemiology and Genetics 2021; 12(2): 16-23.  89. El-Gayar E, Mokhtar A, Awad S, Soliman R, Hassan W. The endosymbiotic relationship between Trichomonas vaginalis and Mycoplasma hominis in Egyptian Women and its correlation with pathogenicity. Parasitol United J 2016; 9(2): 80.  90. El-Gayar EK, Rashwan MF. Cervical intraepithelial neoplasia (CIN) and Trichomonas vaginalis infection as revealed by polymerase chain reaction. J Egypt Soc Parasitol 2007; 37(2): 623-30.  91. el-Naga IF, Khalifa AM, el-Azzouni MZ. In-pouch TV culture system in diagnosis of Trichomonas vaginalis infection. J Egypt Soc Parasitol 2001; 31(3): 647-56 + 1p plate.  92. ElFeky DS, Assiri R, Bakhsh H, et al. Microbiological pattern of laboratory confirmed vaginal infections among Saudi women. Clinical and Experimental Obstetrics and Gynecology 2021; 48(4): 929-34.  93. Elsherif HR, Youssef FMA. Real-time PCR improve detection of Trichomonas vaginalis compared to conventional techniques. Comp Clin Pathol 2012; 22(2): 295-300.  94. Etminan S, Bokaei M. Prevalence of trichomoniasis in women referring to health centers in Yazd. Knowledge and Health 2007; 2(3): 14-20.  95. Ezzat HE. Bacterial vaginosis: diagnosis and associations in vaginal discharge. Medical Journal of Cairo University [The] 1995; 63(4): 997-1005.  96. Fallah M, Mosayebi M, Matini M, Darabi F. Prevalence of Trichomoniasis and Determination of in Vitro Susceptibility of Isolated Parasites to Metronidazole in Women Referred to Health Centers in Arak, Iran, in 2020. Avicenna Journal of Clinical Medicine 2021; 28(3 (SN 101)#r001975): 186-93.  97. Farhan RK. Common causes of vaginal infections and antibiotic sensitivity of aerobic bacterial isolates in reproductive age women attending Tikrit teaching hospital, Salah al-Din Governorate, Iraq. NeuroQuantology 2022; 20(6): 7568-83.  98. Farrukh R, Kamal F, Naheed F, Zafar A, Aslam M. Incidence of Bacterial Vaginosis Among Patients with Vaginal Discharge. Ann King Edward Med Uni 2000; 6-4: 391-3.  99. Fouad SIA-R, Firas MBA. Genotypes Diversity and Virulence Factor screening of Trichomonas vaginalis Isolated from Pregnant Women in Mosul (North of Iraq) التباين الوراثي والتحري عن عامل الضراوة في المشعرات المهبلية المعزولة من النساء الحوامل في الموصل (شمال العراق). Baghdad Science Journal 2022; 19(5): 944-50.  100. Gabr N, Kamal A, Mohamed R, Abdelwahab S. Sensitivity and specificity of wet mount, culture and PCR in diagnosing trichomonas vaginalis infection in females attending the gynecology clinic of Minia University Hospital. El Minia Medical bulletin 2006; 17(1).  101. Ghallab MMI, Alaa D, Morsy SM. Multiattribute Analysis of Trichomonas vaginalis Diagnostics and Its Correlation with Clinical Complaints and Contraceptive Methods in a Symptomatic Egyptian Cohort. Infect Dis Obstet Gynecol 2021; 2021: 5525095.  102. Ghobahi M, Hamedi Y, Shamseddin J, Heydari Hengami M, Sharifi Sarasiabi K. Frequency of Trichomoniasis and Related Risk Factors in theWomen Referred to Bandar Abbas Health Centers, Iran, 2017-2018. Hormozgan medical journal 2019; 23(1).  103. Ghotbi S, Beheshti M, Amirizade S. Causes of Leukorrhea in Fasa, Southern Iran. 2007; 8(2): 58-63.  104. Gouya MM, Nabaei S. Prevalence of some sexually transmitted infections in a family planning service. Razi Journal of Medical Sciences 2007; 14(54): 143-50.  105. Gul F, Faiz NR, Raziq F, et al. Frequency of vaginal discharge and its association with various sexually transmitted diseases in women attending antenatal clinic. Journal of Postgraduate Medical Institute 2005; 19(1): 86-91.  106. Habibi A, Nateghi Rostami M, Douraghi M, Dolati M, Hossein Rashidi B, Ahangari R. Frequency of genital infection with Trichomonas vaginalis in women referred to gynecology hospital of the city of Qom. Dermatology and Cosmetic 2015; 6(4): 190-9.  107. Habibipour R, Amirkhani A, Matinnia N. Contamination Rate Of Trichomonas Vaginalis In Females Referring To Taamin Ejtemayi Hospitals In Hamedan In 2005. Zahedan Journal Of Research In Medical Sciences (Tabib-E-Shargh) 2007; 8(4): 245-51.  108. Haghighi JD, Jafarimodrek M, Sohrabi S, Azizi H, Hatam-Nahavandi K. Trichomoniasis Prevalence at a Care Center Among Women with High-Risk Behaviors in Zahedan, Iran. International Journal of High Risk Behaviors and Addiction 2019; 8(2).  109. Hamdy DA, Hamdy HG. Prevalence, sociodemographic factors and clinical criteria of trichomonas vaginalis infection among symptomatic women in Beni-Suef Governorate, Egypt. Journal of the Egyptian Society of Parasitology 2018; 48(1): 109-17.  110. Hammouda NA, Hegazy IH, Tawfik TA. A rapid diagnostic test for Trichomonas vaginalis infection. J Egypt Soc Parasitol 1997; 27(2): 341-7.  111. Hamouda M, Mohamed S, Elgendy S, Esam Eldeen N, El-Zayady W. Is trichomoniasis associated with adverse preganancy outcome? Parasitol United J 2022; 15(2): 202-9.  112. Hancali Sr A. Prevalence of stis among female sex workers in agadir in the south of Morocco. Sexually Transmitted Diseases 2014; 41(SUPPL. 1): S145-S6.  113. Hanna J, Yassine R, El-Bikai R, et al. Molecular epidemiology and socio-demographic risk factors of sexually transmitted infections among women in Lebanon. BMC Infect Dis 2020; 20(1): 375.  114. Hassan AAM, Ayoub NM. Comparative study between different methods for diagnosing vaginal trichomoniasis. The new Egyptian Journal of Medicine 1993; 8(3): 636-9.  115. Hassan MF, Rund NMA, El-Tohamy O, et al. Does Aerobic Vaginitis Have Adverse Pregnancy Outcomes? Prospective Observational Study. Infect Dis Obstet Gynecol 2020; 2020: 5842150.  116. Hassan MK, Al-Shaheen H, Al-Mukh JM. Bacterial vaginosis and preterm labour. The Medical Journal of Basrah University 2005; 23(1): 42-6.  117. Hassun AF, Jarulla BA. Abortion Related of Infectious Agents in Women in Thi-Qar Province. Journal of thi-qar science 2021; 8(1): 118-24.  118. Hawash YA, Ismail KA, Jaafer NF, Ahmed G, Alpakistany TA, Khalifa OM. Prevalence and Risk Factors for Trichomonas Vaginalis Infection Among Women: a Population-Based Controlled Study in Saudi Arabia. Clin Lab 2022; 68(6).  119. Hawkes S, Collumbien M, Platt L, et al. HIV and other sexually transmitted infections among men, transgenders and women selling sex in two cities in Pakistan: A cross-sectional prevalence survey. Sexually Transmitted Infections 2009; 85(SUPPL. 2).  120. Hazrati Tapeh KH, Mohamad Zadeh H, Mostaghim M, Fereidoni J, Mehri E. A comparative study on the sensitivity of two different diagnostic ways of Diamond culture and wet mount in Trichomonas vaginalis diagnosis and correlation between infection and clinical findings. Studies in Medical Science 2004; 15(1): 7-13.  121. Hegazy AR, El Kersh WM, Moharm IM, Ammar AI, Hemida AS, Atia AF. Immunological and Cytopathological Assessment of Trichomonas vaginalis Infection in Asymptomatic and Symptomatic Females at Menoufia Governorate, Egypt. Int J Curr Microbiol App Sci 2020; 9(4): 686-705.  122. Hegazy MM, El-Tantawy NL, Soliman MM, El-Sadeek ES, El-Nagar HS. Performance of rapid immunochromatographic assay in the diagnosis of Trichomoniasis vaginalis. Diagn Microbiol Infect Dis 2012; 74(1): 49-53.  123. Heikal EA, Elamir AM, Hegazi MA, et al. Signature of real-time PCR in detection of Trichomonas vaginalis infection and its association with human papillomavirus genotype 16. Eur Rev Med Pharmacol Sci 2023; 27(2): 501-10.  124. Heikel J, Sekkat S, Bouqdir F, et al. The prevalence of sexually transmitted pathogens in patients presenting to a casablanca STD clinic. European Journal of Epidemiology 1999; 15(8): 711-5.  125. Hosny A, El-Khayat W, Kashef MT, Fakhry MN. Association between preterm labor and genitourinary tract infections caused by Trichomonas vaginalis, Mycoplasma hominis, Gram-negative bacilli, and coryneforms. J Chin Med Assoc 2017; 80(9): 575-81.  126. Hussein AH, Saleh MH, Nagaty IM, Ghieth KA, El-Azab NA. Prevalence, clinical criteria and sociodemographic predictors of Trichomonas vaginalis infection in suspected Egyptian women, using direct diagnostic techniques. Iranian Journal of Parasitology 2015; 10(3): 432-40.  127. Hussein EM, Salm AM, Rashwan M. Biological variability of trichomonas vaginalis clinical isolates fom symptomatic and asymptomatic patients. Journal of Egyptian Society of Parasitology 2004; 34(3): 979-88.  128. Ibrahim SS, Ismail MA, Elaskary HM, Khalil EM, Khalil D, Raafat A. Potential Role Of Trichomonas Vaginalis In Women With Primary And Secondary Infertility In Beni-Suef, Egypt. Journal of the Egyptian Society of Parasitology 2021; 51(1): 119-26.  129. Jaafar NK, Kadhum TJ, Ismael I. Study Of Causative Agents Of Cervicitis In Women Attending Gynecologic Outpatient Department In Najaf City دراسة مسببات التهاب عنق الرحم لدى النساء الوافدات إلى قسم النسائية في مستشفيات محافظة النجف. Kufa Medical Journal 2008; 11(1): 166-74.  130. Jamali R, Zarei Kar B, Ghazanchaei A, Yousefi S. Comparison of direct microscopic examination and culture methods sensitivity for diagnosis of Trichomonas vaginalis in Tabriz health care centers visitors. Yafteh 2007; 8(4 (30)): 63-8.  131. Juma A, Al Sheikh S, Al Jeboori T. The levels of cooper and zinc in the serum of women with trichomonas vaginalis. Iraqi Journal of Medical Sciences 2003; 2(3): 263-6.  132. Kadhum NJ, Al-Mayah SH, Raisan SJ. Epidemiological study on trichomonas vaginalis among the women who attended the hospitals of Basra province. Journal of Basrah Researches (Sciences) 2020; 46(2): 64-73.  133. Kadir M, Aziz LJ. A study on trichomonas vaginals infection in Kirkur-Iraq. Bulletin of Endemic Disease-Baghdad 1989; 30: 1-8.  134. Kadir MA, Ghalib AK, Tahir SSH, Al-Dalableh f. A study on trichomonas vaginalis and comparison between the efficacy of metronidazole and secnizole on women in Kirkuk province. Journal of the Faculty of Medicine 2006; 48(1): 94-7.  135. Kadir MA-A, Hamad NR. Biochemical parameters in patients with Trichomonasvaginalis and Toxoplasma gondii in Erbil-Iraq. Tikret Journal of Pharmaceutical Sciences 2013; 9(2): 327-37.  136. Kadir MAA, Kadir S. Prevalence of trichomonas vaginalis among females with vaginal discharge in tikrit cry. Iraqi Journal of Microbiology 1998; 10(2): 36-47.  137. Kafi SK, Mohamed AO, Musa HA. Prevalence of sexually transmitted diseases (STD) among women in a suburban Sudanese community. Ups J Med Sci 2000; 105(3): 249-53.  138. Kahkhayi KR, Barouni F. Evaluation of the Effect of Myrtus Comminus Extract on Trichomonas Vaginalis. Journal of Zabul Medical School 2020: 29-33.  139. Kalantari N, Ghaffari S, Esmaeilzadeh S. The frequency study of trichomoniasis in women referred to gynecology clinic of Ayatollah Rohani Hospital, Babol, Iran, in 2010. Annals of Tropical Medicine and Public Health 2012; 5(5): 498-501.  140. Kamal AM, Ahmed AK, Mowafy NMES, Shawki HE, Sanad AS, Hassan EE. Incidence of antenatal trichomoniasis and evaluation of its role as a cause of preterm birth in pregnant women referring to Minia University hospital, Egypt. Iranian Journal of Parasitology 2018; 13(1): 58-66.  141. Kareem HK, Hamad MM, Hasan MA, Abd alsammed MA. Study on Trichomonas vaginalis infection in women with type-2 diabetes mellitus and vaginal discharge in Thi-Qar Government. European Journal of Molecular and Clinical Medicine 2020; 7(8): 4471-8.  142. Kazerooni T, Mosalaee A. Does contraceptive method change the Pap smear finding? Contraception 2002; 66(4): 243-6.  143. Kermasha ZW, Al-Masoudi HK, Al-Shaikh SF. Association of TLR7 and MyD88 Gene Polymorphism with Trichomoniasis vaginalis Infection. Journal of Contemporary Medical Sciences مجلة العلوم الطبية المعاصرة 2023; 09(04): 304-10.  144. Khalaf A, K. H., Al-Asadi S, A. M., Al-Yaaqub AJ, Al-Mayah SH. Use Pcr Technique To Detect Trichomonas Vaginalis Among Men In Basrah Province استخدام تقنية تفاعل البلمرة التسلسلي PCR في الكشف عن طفيلي المشعرة المهبلية Trichomonas vaginalis بين الرجال في محافظة البصرة. Thi-Qar Medical Journal 2010b; 4(2): 29-36.  145. Khalaf AK, Al Kayat ES. Study the association between the infection with Trichomonas vaginalis and use of contraceptive among women with abnormal vaginal discharge by PCR technique in Nassiriyah city دراسة العلاقة بين التهابات التراكومونس مع استخدام موانع الحمل بين النساء وذوات الافراز المهبلي غير الطبيعي. Thi-Qar Medical Journal 2015; 9(1): 95-101.  146. Khalaf AK, Al-Nasir AHA, Al-Khayat ES. Use PCR technique to detect the infection with Trichomonas vaginalis among women with preterm labor استخدام تقنية تفاعل البلمرة التسلسلي في الكشف عن طفيلي المشهرة المهبلية (Trichomonas vaginalis) بين النساء ذوات الولادة المبكرة. Thi-Qar Medical Journal 2016; 11(1): 156-61.  147. Khalaf AK, Kadhim KJ. Use TVK 3/7 gene as a target to detect Trichomonas vaginalis from urine of women in Southern Iraq استخدام الجين TVK3/7 كمستهدف في الكشف عن طفيلي المشعرة المهبلية Trichomonas vaginalis من إدرار النساء في جنوب العراق. Thi-Qar Medical Journal 2010a; 4(1): 36-46.  148. Khalaf AKH. Detection of Trichomonas vaginalis among women with abnormal vaginal discharge by PCR technique targeting TVK3 and TVK7 genes in Basrah province تشخيص الاصابة بطفيلي المشعرة المهبلية بين النساء اللاتي يعانين من الافراز المهبلي غير الطبيعي في محافظة البصرة باستخدام تقنيةPCR. Thi-Qar Medical Journal 2013; 7(1): 41-8.  149. Khalil HI, Al-Kuraishi AH, Al-Naimi UAM, Al-Naimi SA. Trichomoniasis Vaginalis in Women Attending Family Planning Unit in AL-Liqa'a Hospital داء المشعرات المهبلية لدى النساء المراجعات لوحدة تنظيم الاسرة في مستشفى اللقاء. Iraqi Journal of Science 2012; 53(4): 746-653.  150. Khalili B, Ghasemi-Dehkordi P, Pourshahbazi G, Yousofi-Darani H, Hashemzadeh-Chaleshtori M, Doosti A. Genotyping of Trichomonas vaginalis isolates from women in Shahrekord city (Southwestern Iran). Genetika-Belgrade 2017; 49(3): 1059-70.  151. Khan FZ. Microbial infections in females of childbearing age and therapeutic interventions. Rawal Med J 2011a; 36(3): 178-81.  152. Khan MS, Unemo M, Zaman S, Lundborg CS. HIV, STI prevalence and risk behaviours among women selling sex in Lahore, Pakistan. BMC Infect Dis 2011b; 11: 119.  153. Khaskheli M, Baloch S, Baloch AS. Vaginal discharge during pregnancy and associated adverse maternal and perinatal outcomes. Pak J Med Sci 2021; 37(5): 1302-8.  154. Khezri M, Shokoohi M, Mirzazadeh A, et al. Early sex work initiation and its association with condomless sex and sexually transmitted infections among female sex workers in Iran. International Journal of STD and AIDS 2020; 31(7): 671-9.  155. Latif AS, Magtooph MG, Raheem IA. Relationship between lead contaminations with the cervical inflammatory in iraqi women of baghdad. Biochemical and Cellular Archives 2020; 20(Supplement2): 4287-93.  156. Maghsoudi R, Danesh A, Kabiri N, Setorki M, Doudi M. Prevalence of the genital tract bacterial infections after vaginal reconstructive surgery. Pak J Biol Sci 2014; 17(9): 1058-63.  157. Mahafzah AM, Al-Ramahi MQ, Asa'd AM, El-Khateeb MS. Prevalence of sexually transmitted infections among sexually active Jordanian females. Sex Transm Dis 2008; 35(6): 607-10.  158. Maharlouei N, Barooti E, Sharif F, Hosseini H, Lankarani KB. Prevalence and risk factors of reproductive tract infections among a defined population of Iranian women. Sexual Health 2013; 10(4): 311-5.  159. Mahdey AS, Abd FG. Molecular Detection the Agent that Causing Vaginitis in Vaginal Secretion from Women with Vaginitis and it Relation with Abortion. Journal of University of Babylon 2018; 26(6): 253-62.  160. Mahdi N, Al Hamdani M. Sexually transmitted diseases among women with habitual abortion. Eastern Mediterranean Health Journal 1998; 4(2): 343-9.  161. Mahdi NK. Urogenital trichomoniasis in an Iraqi population. Eastern Mediterranean Health Journal 1996; 2(3).  162. Mahdi NK, Gany ZH, Sharief M. Risk factors for vaginal trichomoniasis among women in Basra, Iraq. East Mediterr Health J 2001; 7(6): 918-24.  163. Mahmoud A, Sherif NA, Abdella R, El-Genedy AR, El Kateb AY, Askalani AN. Prevalence of Trichomonas vaginalis infection among Egyptian women using culture and Latex agglutination: cross-sectional study. BMC Womens Health 2015; 15: 7.  164. Mahmoud DM. A study of the prevalence of trichomonas vaginalis in women with the evaluation of different laboratory techniques used for its detection. Egypion journal of Medical Microbiology 1996; 5(3): 399-404.  165. Mahmoud MS, Abdel-Aziz SS, El-Sherif EA, Swidan KH. Diagnosis of symptomatic and asymptomatic Trichomonas vaginalis infection by applying one tube nested PCR to vaginal discharge. J Egypt Soc Parasitol 1999; 29(3): 1031-46.  166. Manshoori A, Mirzaei S, Valadkhani Z, et al. A diagnostic and symptomatological study on trichomoniasis in symptomatic pregnant women in Rafsanjan, south central Iran in 2012-13. Iranian Journal of Parasitology 2015; 10(3): 490-7.  167. Maraghi S, Khosravi A, Kardooni T, Razi T, Feiz-Haddad MH. Evaluation of an Immunochromatographic Strip (Xenostrip - Tv) Test for Diagnosis of Vaginal Trichomoniasis Compared with Wet Mount and PCR Assay. Iranian Journal of Parasitology 2008; 3(3): 11-7.  168. Matini M, Golmoradi K, Maghsood AH, Fallah M. The prevalence of trichomoniasis and metronidazole susceptibility of the isolates in Ghorveh, year 2015. Avicenna Journal of Clinical Medicine 2016; 23(3 (SN 81)): 185-92.  169. Matini M, Rezaei H, Fallah M, Maghsood AH, Saidijam M, Shamsi-Ehsan T. Genotyping, drug susceptibility and prevalence survey of Trichomonas vaginalis among women attending gynecology clinics in Hamadan, Western Iran, in 2014-2015. Iranian Journal of Parasitology 2017; 12(1): 29-37.  170. Matini M, Rezaeian M, Mohebali M, et al. Genotyping of Trichomonas vaginalis isolates in Iran by using single stranded conformational polymorphism-PCR technique and internal transcribed spacer regions. Trop Biomed 2012; 29(4): 605-12.  171. Mazloumi Gavgani AAS, Namazi A, Ghazanchaei A, et al. Prevalence and risk factors of trichomoniasis among women in Tabriz. Iranian journal of clinical infectious diseases 2008; 3(2): 67-71.  172. Merdaw MA-Z, Al-Mayah QS, Malik SN, Al-Bashier NM. Serum Levels of Prolactin and Complement Components (C3 and C4) in Women Infected with Trichomonas vaginalis. Iraqi Journal of Biotechnology 2015; 14(2): 30-6.  173. Ministry of Health - Morocco. HIV integrated behavioral and biological surveillance surveys - Morocco 2011, 2011.  174. Moaiedmohseni S, Bashardoost L, Abbasi M. Cervicovaginal infections during third trimester of pregnancy. Journal Of Family and Reproductive Health 2012: 11-5.  175. Mobasheri M, Saeedi Varnamkhast N, Karimi A, Banaeiyan S. Prevalence study of genital tract infections in pregnant women referred to health centers in Iran. Turk J Med Sci 2014; 44(2): 232-6.  176. Mohamad AN, Eskandar AY, Laftah AA. Prevalence of trichomoniasis in baquba city. Al-Mustansiriyah Journal of Science 2007; 18(3): 19-23.  177. Mohammadi Ghalehbin B, Rahimi K, Pezeshki A, et al. Frequency of Trichomonas vaginalis infection among pregnant women referred to health and medical centers in Ardabil city, 2013-2014. Journal of Ardabil university of Medical Sciences (JAUMS) 2015; 15(1 #G00162): 75-82.  178. Mohammed BO, Khalifa KE, Elleboudy NA, Hussein HM, Azab ME. Growth kinetics of Egyptian isolates of Trichomonas vaginalis: Possible correlation to clinical presentation. The Egyptian Journal of Hospital Medicine 2018; 72(5): 4428-33.  179. Morshedloo L, Fallah M, Maghsood AH, Matini M. Study of Trichomonas vaginalis infection in women visiting health centers in Bahar city and determination of metronidazole susceptibility of the isolated parasites. Avicenna Journal of Clinical Medicine 2018; 24(4 (SN 86) #M00156): 315-21.  180. Moshfe A, Ghaffari P, Khoramrooz SS, Bab t, Akbarzadeh A, Ghareghani M. Frequency of Mutations in Friedoxin Gene in Trichomonas Vaginalis Isolated From Women With Vaginitis Referred to Yasuj Gynecologic Clinic in 2017. Armaghan Danesh 2019; 23(5 (130) #f00836): 608-18.  181. Moshfe AA, Hosseini S. Comparison of clinical and microscopic diagnosis of Trichomoniasis referred to the Yasouj women clinic. Armaghan Danesh 2004; 9(33): 81-5.  182. Motazedian MH, Agholi M, Kalantari M, Hatam GR. High rates of Trichomoas vaginalis among HIV-infected patients in fars province, southern Iran. Tropical Medicine and International Health 2011; 16(SUPPL. 1): 154.  183. Mousaviani ZAS, Esmaeili I, Behbahani SMR. Diagnosing contamination and determining effective factors on contraction of Trichomonas vaginalis and gonorrhea in female prisoners at Evin jail Tehran, 2004. Research Bulletin of Medical Sciences 2005; 9(5 (41)): 301-3.  184. Mozher HM. عزل وتشخيص بعض انواع الاحياء المجهرية المسببة لالتهابات المهبل المرافقة لاصابات القناة التناسلية الانثوية ودراسة تأثير بعض العوامل على انتشارها. Journal of Education for Pure Science 2011; 1(4): 147-57.  185. Mushref E, Ardalan NM, Ahmed ZaA-R. Trichomonas vaginalis used as a marker for other sexually transmitted infections in women إمكانية استخدام طفيلي المشعرات المهبليه كدليل لأصابات جنسية اخرى عند النساء. Iraqi Journal of Community Medicine 2010; 23(4): 292-4.  186. Mushref E, Jassim AN, Adhiah AH. Evaluation the efficiency of Trichomonas vaginalis depending on clinical sings , direct examination ,culturing and serological test تقييم كفاءة تشخيص المشعرات المهبلية vaginalis Trichomonasبالأعتماد على الاعراض السريرية والفحص المباشر و الزرع والفحص السريرولوجي. Baghdad Science Journal 2011; 8(1عدد خاص بمؤتمر علوم الحياة): 392-9.  187. Naama JK, Hasson KF, Abdullah EE. Detection of Trichomonas Vaginals among women with contraceptive usage in AL-Najaf AL-ashraf city أنتشار داء المشعرات في النساء المستخدمة موانع الحمل في النجف الأشرف. Thi-Qar Medical Journal 2008; 2(1): 46-50.  188. Nagaty HF, Salem SA. Trichomoniasis in Egypt. I. Incidence of vaginal infestation among attendants of Ein-Shams Hospital. J Egypt Med Assoc 1962; 45: 282-91.  189. Nasir JA, J N, F T, Asghar N, Iqbal J. Trichomonas vaginalis in vaginal smears of women using intrauterine contraceptive device. Pak J Med Res 2005; 44(3): 114-6.  190. Nasir MAH, S.R A-I, Al-Masoudi WA. Comparison of Different Techniques for the Diagnosis of Trichomonas Vaginalis Infection in Females at Reproductive Age. Pakistan Journal of Medical and Health Sciences 2022; 16(7): 451-4.  191. Nassef NE, Afif AF, Basuni AA, El-Nasr MFA, Atia AF. Evaluation of microscopy and polymerase chain reaction for diagnosis of symptomatic and asymptomatic female trichomoniasis. Parasitol United J 2014; 7(1): 37.  192. Nateghi Rostami M, Hossein Rashidi B, Nazari R, Aghsaghloo F, Habibi A. A multiplex assay of Trichomonas vaginalis, Chlamydia trachomatis and Neisseria gonorrhoeae infections in genital specimens. J Infect Dev Ctries 2017; 11(11): 833-9.  193. Nazari N, Rahimi MA, Bayat E. Prevalence of Trichomonas Vaginalis in Diabetic Females. mljgoums 2014; 8(3): 110-3.  194. Nazari N, Zangeneh M, Moradi F, Bozorgomid A. Prevalence of trichomoniasis among women in Kermanshah, Iran. Iranian Red Crescent Medical Journal 2015; 17(3): 1-4.  195. Nikpay S, Otaghi M, Azami M, Karimi M, Abdi J. Trichomonas Vaginalis Infection Among Women Attending Laboratory Centers in Ilam, Iran. Infect Disord Drug Targets 2020; 20(1): 98-101.  196. Nouraddin AS, Alsakee HM. Prevalence of Trichomonas vaginalis infection among women in Erbil governorate, Northern Iraq: An epidemiological approach. European Scientific Journal 2015; 11(24): 243-55.  197. Nourian A, Shabani N, Fazaeli A, Mousavinasab SN. Prevalence of Trichomonas vaginalis in pregnant women in Zanjan, Northwest of Iran. Jundishapur Journal of Microbiology 2013; 6(8): e7258.  198. Omer EE, Ali MH, Erwa HH. Study of sexually transmitted disease in Sudanese women. Trop Doct 1980; 10(3): 99-102.  199. Omer EE, Catterall RD, Ali MH, Erwa HH. Incidence of urogenital trichomoniasis among high risk Sudanese groups. East Afr Med J 1984; 61(2): 140-4.  200. Omer EE, Naeem HA, Ali MH, Catterall RD, Erwa HH. Vaginal and cervical abnormalities associated with trichomonal infection. East Afr Med J 1991; 68(6): 455-60.  201. Omer EF, Catterall RD, Ali MH, el-Naeem HA, Erwa HH. Vaginal trichomoniasis at a sexually transmitted disease clinic at Khartoum. Trop Doct 1985a; 15(4): 170-2.  202. Omer EF, el-Naeem HA, Ali MH, Catterall RD, Erwa HH. Evaluation of the laboratory diagnosis of vaginal trichomoniasis in Khartoum. J Trop Med Hyg 1988; 91(6): 292-5.  203. Omer EFEO, El-Naeem HAR, Ali MH. Micro-organisms associated with vaginal trichomoniasis among Sudanese women. Saudi Medical Journal 1985b; 6(2): 129-34.  204. Ortashi OM, El Khidir I, Herieka E. Prevalence of HIV, syphilis, Chlamydia trachomatis, Neisseria gonorrhoea, Trichomonas vaginalis and candidiasis among pregnant women attending an antenatal clinic in Khartoum, Sudan. J Obstet Gynaecol 2004; 24(5): 513-5.  205. Payamani S, Dalimi A, Gaffarifar F. A study on trichomoniasis in HIV positive women in Tehran, Iran. Iranian Journal of Parasitology 2017; 13(1): 92.  206. Philip A, Shouman AE, Dewedar SA, Boulos DN. Prevalence of the most common Reproductive Tract Infections among women attending family Planning clinics in Montazah-Alexandria. infection 2015; 1(2): 3.  207. Rabiee S, Fallah M, Zahabi F. Frequency of trichomoniasis in patients admitted to outpatient clinics in Hamadan (2007) and relationship between clinical diagnosis and laboratory findings. Journal of Research in Health Sciences 2010; 10(1): 31-5.  208. Rafiei A, Safaie K, Tavalla M, Najafian M. PCR Detection and Sequencing of Trichomonas vaginalis in Women with Suspected Vaginitis in Southwestern Iran. Infect Disord Drug Targets 2021; 21(2): 262-7.  209. Rajabpour M, Emamie AD, Pourmand MR, Goodarzi NN, Asbagh FA, Whiley DM. Chlamydia trachomatis, Neisseria gonorrhoeae, and Trichomonas vaginalis among women with genitourinary infection and pregnancy-related complications in Tehran: A cross-sectional study. Int J STD AIDS 2020; 31(8): 773-80.  210. Rakha AE, El-Shazly SA, Abdel Gawad A, Mokhtar AA. A study of some causative agents of leukorrhea among females at the child bearing period in Alexandria. The Bulletin of the High Institute of Public Health 1987; 17(2): 149-61.  211. Ramezanian R, Assmar M, Valadkhani Z. Seroparasitological investigation of trichomoniasis in women referred to healthcare centers of Rasht city. Iranian Journal of Medical Microbiology 2017; 11(3): 78-84.  212. Ramia S, Kobeissi L, El Kak F, Shamra S, Kreidieh K, Zurayk H. Reproductive tract infections (RTIs) among married non-pregnant women living in a low-income suburb of Beirut, Lebanon. J Infect Dev Ctries 2012; 6(9): 680-3.  213. Rasti S, Arbabi M, Khakbazan S, Khamechian T, Hooshyar H, Yadegarifard G. Epidemiology of Trichomoniasis in women referring to health and therapeutic centers of Kashan in 1372 and 1373. FEYZ 2000; 3(4): 104-10.  214. Rasti S, Taghriri A, Behrashi M. Trichomoniasis in parturients referring to Shabihkhani hospital in Kashan, 2001-02. FEYZ 2003; 7(2): 21-5.  215. Rehan N, Bokhari A, Nizamani NM, et al. National study of reproductive tract infections among high risk groups of Lahore and Karachi. J Coll Physicians Surg Pak 2009; 19(4): 228-31.  216. Rezaeian M, Vatanshenassan M, Rezaie S, et al. Prevalence of Trichomonas vaginalis using parasitological methods in Tehran. Iranian Journal of Parasitology 2009; 4(4): 43-7.  217. Riyam B. Ali KKG. Molecular Detection of Some Sexually Transmitted Bacteria and Trichomonas vaginalis in Iraqi Married Couples. Iraqi Journal of Biotechnology 2022; 21(2): 136-44.  218. Rizvi S, Khan MS, Khaskheli QA, Agha ZA, Sabir M. Frequency of bacterial Vaginosis and its rapid diagnosis in married females of child bearing age. Med Channel 2006; 12(2): 62-3.  219. Rizvi TH, Hassan FH, Surryia S, Sha SSA. Vaginal infection and birth weight. Pak J Med Res 2003; 42(1): 7-9.  220. Rostami MN, Rashidi BH, Habibi A, Nazari R, Dolati M. Genital infections and reproductive complications associated with trichomonas vaginalis, Neisseria gonorrhoeae, and Streptococcus agalactiae in women of Qom, central Iran. International Journal of Reproductive BioMedicine 2017; 15(6): 357-66.  221. Ryan CA, Zidouh A, Manhart LE, et al. Reproductive tract infections in primary healthcare, family planning, and dermatovenereology clinics: Evaluation of syndromic management in Morocco. Sexually Transmitted Infections 1998; 74(SUPPL. 1): S95-S105.  222. Saba Fadhil A. Incidence of trichomonus vaginalis infection after menopause age of womenحدوث الإصابة بالمشعرات المهبلية بعد سن اليأس في النساء. Journal of Techniques 2012; 25(2): 16-20.  223. Saber N, Saraei M, Hajialilo E, et al. Screening and molecular characterization of Trichomonas vaginalis genotypes isolated from married women in northern Iran. Ann Parasitol 2022; 68(3): 587-94.  224. Sadiq AM, Youssif MG. Vaginal leucocyte counts as indicator for cervical infections in women with bacterial vaginosis in Najaf, Iraq تعداد الخلايا البيضاء المهبلية دليل لإصابات عنق الرحم لدى النساء المصابات بالتهاب المهبل البكتيري في محافظة النجف, العراق. Kufa Medical Journal 2008; 11(1): 110-20.  225. Salar Z, Saeeda M, Johar K. Microflora in pregnancy. JPMA-Journal of Pakistan Medical Association 1986; 36(4): 79-81.  226. Saleh AM, Abdalla HS, Satti AB, Babiker SM, Gasim GI, Adam I. Diagnosis of Trichomonous vaginalis by microscopy, latex agglutination, diamond's media, and PCR in symptomatic women, Khartoum, Sudan. Diagn Pathol 2014; 9: 49.  227. Salem SA, Azab M, Safer E, Aboul-Magd L. A preliminary study of antibodies against Trichomonas vaginalis infection using the IFAT. Journal of the Egyptian Society of Parasitology 1981; 11(1): 201-6.  228. Salih AA, Obaid HM, Jasim W, M. . An in vitro study of zinc effect on Trichomonas vaginalis isolated from infected women. NTU Journal of Pure Sciences 2022; 1(4): 44-52.  229. Sallam MA, El Sharkawy EM. Trichomonas vaginalis is not a rare sexually-transmitted disease among Egyptian men. Scientific Journal of Al-Azhar Medical Faculty [Girls] [The] 2003; 24(1): 373-80.  230. Sallam S, Ali OT, Hassan MN, Fares E. Epidemiology of gonorrhea among married females presenting with leucorrhoea. Bulletin of High Institute of Public Health 1982; 12(3): 65-80.  231. Sallam SA, Mahfouz AA, Dabbous NI, el-Barrawy M, el-Said MM. Reproductive tract infections among married women in Upper Egypt. East Mediterr Health J 2001; 7(1-2): 139-46.  232. Salman ST, Hussein AA. Contraception as a Risk Factor of Trichomonas vaginalis Infection Among Women Attending Outpatient of Al-Batool Teaching Hospital for Maternity and Children-Baqubah-Iraq وسائل منع الحمل كعامل خطورة للاصابة بداء المشعرات المهبلية للنساء اللواتي يراجعن العيادة الخارجية لمستشفى البتول التعليمي للأمومة والطفولة في ديالى/ العراق. Al-Kindy College Medical Journal 2017; 13(1): 20-6.  233. Salmani R, Baghchesaraie H, Amini B. Prevalence of Trichomonas vaginalis infection among women refered to laboratories in Zanjan, 2010. Journal of Research development in Nursing and Midwifery 2012; 9(1): 69-75.  234. Shahbazi A, Falah E, Safaian R. Infection rate of Trichomonas vaginalis in females referring to Tabriz and Basmeng health care centers, 1998-99. Pajouhesh Dar Pezeskhi 2002; 25(4): 231-4.  235. Shahnazi E, Mohammadzadeh H, Daneshyar C, Chavshin A, Khademvatan S. Frequency and molecular diagnosis of trichomoniasis in symptomatic women referred to laboratories in urmia north west Iran. Journal of Acute Disease 2017; 6(4): 175-80.  236. Shahraki F, Fouladi B, Salimi-Khorashad A, Sepehri-Rad N, Dabirzadeh M. Epidemiology and identification of actin gene of Trichomonas vaginalis genotypes in women of southeast of Iran using PCR-RFLP. Crescent Journal of Medical and Biological Sciences 2020; 7(1): 82-90.  237. Shaker EM, Almaeahi AMY, Al-Shaibani KTM. Epidemiological study of trichomoniasis with the effect of estrogen hormones lh and fsh among married women in samarra city, iraq. International Journal of Research in Pharmaceutical Sciences 2019; 10(1): 372-7.  238. Shallal MM, Mohammed Ali YN, Salih Al-Asadi FAH. Evaluation Of Most Common Microorganisms Associated with Ectopic Pregnancy by Real Time PCR Among Iraqi Women. Journal of Pharmaceutical Negative Results 2022; 13(3): 680-4.  239. Sharbatdaran M, Shefaee SH, Sami H, et al. Comparison of clinical presentations, wet smear, papanicolaou smear with Dorset's culture for diagnosis of Trichomonas vaginalis in doubtful women to trichomoniasis. Journal of Babol University of Medical Sciences 2005; 7(3 (27)): 46-9.  240. Sharifi I, Khatami M, Tahmors-Kermani E. Prevalence of trichomonas vaginalis in women referred to vali- asr polyclinic and the health center number 3 in Sirjan city. Journal of Kerman University of Medical Sciences 1994; 1(3): 125-34.  241. Shawaky SM, Al Shammari MMA, Sewelliam MS, Ghazal A, Amer AN. A study on vaginitis among pregnant and non-pregnant females in Alexandria, Egypt: An unexpected high rate of mixed vaginal infection. AIMS Microbiol 2022; 8(2): 167-77.  242. Sheetawy AZ, Abdulla ZA. Detection of chlamydia and other bacteria in cervicitis. Annals of the College of Medicine Mosul 2007; 33(1&amp;2): 26-34.  243. Shipitsyna E, Kularatne R, Golparian D, et al. Mycoplasma genitalium prevalence, antimicrobial resistance-associated mutations, and coinfections with non-viral sexually transmitted infections in high-risk populations in Guatemala, Malta, Morocco, Peru and South Africa, 2019-2021. Frontiers in Microbiology 2023; 14: 1130762.  244. Shobeiri F, Nazari M. A prospective study of genital infections in Hamedan, Iran. Southeast Asian J Trop Med Public Health 2006; 37 Suppl 3: 174-7.  245. Tabatabaie F, Kermanjani A, Maleki F, et al. Prevalence of trichomonas vaginalis and candida albicans infections Among Women in Karaj City in Alborz Province, Iran (2012-2013). Journal of Pure and Applied Microbiology 2014; 8: 141-5.  246. Taher JH. Epidemiological and Biological Variability in Clinical Isolates of Trichomonasvaginalis among Women in Najaf/ Iraq, الوبائية والتغايرات الحيوية لعزلات سريرية لطفيلي المشعرة المهبلية لدى النساء في محافظة النجف / العراق. Kerbala journal of pharmaceutical sciences 2012; (3): 23-33.  247. Taher JHT, Shaker M, A. Epidemiological Study of Trichomonas vaginalis and Other Microorganisms Isolated from Genital Tract of Women in Najaf Province – Iraq دراسة وبائية المشعرة المهبلية والأحياء المجهريه الاخرى المعزولة من القناة التناسليه للنساء في محافظة النجف / العراق. Al-Kufa University Journal for Biology 2018; 10(2): 1-9.  248. Talari S, Kazemi B, Hooshyar H, et al. Detection of drug resistance gene in trichomonas vaginalis by PCR. Feyz Journals of Kashan University of Medical Sciences 2011; 15(1).  249. Tavakoli OR, Babaei Z, Hatam GR, et al. Considerable genetic diversity of Trichomonas vaginalis clinical isolates in a targeted population in South of Iran. Iranian Journal of Parasitology 2017; 12(2): 251-9.  250. Valadkhani Z, Assmar M, Hassan N, et al. The prevalence of trichomoniasis in high-risk behavior women attending the clinics of tehran province penitentiaries. Iranian Journal of Medical Sciences 2010; 35(3): 190-4.  251. Valadkhani Z, Safaee Z, Sohrabi M. Prevalence of Trichomonas vaginalis infection among Iranian women using P270 gene. Tropical Medicine and International Health 2015; 20(SUPPL. 1): 284-5.  252. Valadkhani ZT, Asmar M, Esfandiari B, et al. Trichomoniasis in Asymptomatic Patients. Iranian journal of public health 2008; 37(3): 113-7.  253. Wijdan Dhaidan Shnain Al- Abbas وجدان ض, Ohood Aqeed Radhi عهود ع. Incidence of Chlamydia trachomatis and Trichomonas Vaginalis Genital Infections among Non-Pregnant Women in Al - Najaf Province الإصابة بالكلاميديا الحثرية والتريكوموناس المهبلية التناسلية لدى النساء غير الحوامل في محافظة النجف. kufa Journal for Nursing sciences 2019; 9(1): 1-8.  254. Yarizadeh M, Taherkhani H, Amir-Zargar MA, Matini M. Molecular epidemiologic study of male trichomoniasis in hamadan, western iran. Iranian Journal of Parasitology 2021; 16(2): 245-52.  255. Yaseen SAS. Study About the Causative Agents of Cervical Infections and Cytopathological Changes in Iraqi Women. Iraqi Journal of Science 2020; 61(2): 246-53.  256. Younis H, Al Tae AR, Khaki II. Comparative study for the diagnosis of trichomonas vaginalis. IPMJ-Iraqi Postgraduate Medical Journal 2003; 2(4): 394-8.  257. Yousofi Darani H, Ahmadi F, Zebardast N, Yousefi HA, Shirzad H. Development of a Latex Agglutination Test as a Simple and Rapid Method for Diagnosis of Trichomonas vaginalis Infection. Avicenna Journal of Medical Biotechnology (AJMB) 2010; 2(1): 63-6.  258. Zaki ESM, Raafat D, El Emshaty W, Azab MS, Goda H. Correlation of Trichomonas vaginalis to bacterial vaginosis: a laboratory-based study. J Infect Dev Ctries 2010; 4(3): 156-63.  259. Zaki MM, Moussa HM, Hassanin OM. Evaluation of the OSOM Trichomonas rapid test for detection of Trichomoniasis vaginalis. PUJ-Parasitologists United Journal 2011; 4(2): 177-84.  260. Zangiabadi M, Qureshi M, Khoushideh M, Roudbari M, Bahrami SH. Survey of sensitivity of wet smear and Dorset medium in comparison with Diamond medium for diagnosis of Trichomonas vaginalis. Zahedan Journal of Research in Medical Sciences 2002; 4(3): 141-7.  261. Zarandi MB, Fard SRN, Parastouei K, Ahmadi A. Causative Agents of Vaginitis in Women of Kerman Province, Iran. Journal of medical microbiology and infectious diseases 2019; 7(1-2): 29-31.  262. Ziaei Hezarjaribi H, Taghavi M, Hasanjani Saravi K, et al. Actin Gene-Based Molecular Typing of Trichomonas vaginalis Clinical Isolates from the North of Iran. Acta Parasitol 2020; 65(4): 859-64.  263. Zribi M, Ben Mansour K, Abid F, Masmoudi A, Fendri C. Syndromic approach to sexually transmitted infections in Tunisian women: Bacteriological validation. International Journal of STD and AIDS 2008; 19(2): 112-4. |
| --- |

# **Box S6.** List of publications that satisfied the stringent inclusion criteria and from which *Trichomonas vaginalis* prevalence measures were extracted.

| 1. Abd el Ghaffar FM, Azab ME, Salem SA, Habib KS, Maklad KM, Habib FS. Evaluation of two cultural media (CPLM & TYM) for isolation and maintenance of Trichomonas vaginalis stocks in the laboratory. J Egypt Soc Parasitol 1994; 24(3): 611-9.  2. Abdel-Magied AA, El-Kholya EI, Abou El-Khair SM, et al. The genetic diversity of metronidazole susceptibility in Trichomonas vaginalis clinical isolates in an Egyptian population. Parasitol Res 2017; 116(11): 3125-30.  3. Abdu Elrab A, Al Wazer I, Al Duminy A, Al Awady H, Al Shamy H. Primary report on sexually transmitted diseases among women in Sana'a Yemen during 2003-2004. YHMRJ-Yemeni Health and Medical Research Journal 2004.  4. Abdul Jabbar ZR, Al-Warid HS. Some Clinical and Inflammatory Aspects of Trichomonas vaginalis Infection among Women with Pelvic Inflammatory Diseases. Iraqi Journal of Science 2021; 62(12): 4649-66.  5. Abdulkhudher NA, Yousif MG, DSadiq AM. Detection of immunological markers for Chlamydia trachomatis and Trichomonas vaginalis infection in women with obstetric complications in Najaf, Iraq التحري عن الدلائل المناعية لاصابات بكتريا trachomatis Chlamydia والمشعرات المهبلية vaginalis Trichomonas لدى النساء اللواتي لديهن مضعاعفات الحمل في محافظة النجف , العراق. Al-Qadisiyah Journal of Pure Science 2014; 19(2): 14-25.  6. Abdullah N, Abbas G, Mahmood K. Frequency of Common Pathogens in Vaginal Discharge. Age (Years) 2015; 13: 20.  7. Aboulghar M, Aboushady O, Ahmed J, Hanafy N. Diagnosis of trichomonas vaginalis infection in women of childbearing age at a university setting using osom a new diagnostic technique. Egyptian Journal of Medical Microbiology, 2009; 18(9): 51-6.  8. Ahmadi A, Farhadifar F, Rezaii M, et al. Group B streptococci and trichomonas vaginalis infections in pregnant women and those with spontaneous abortion at Sanandaj, Iran. Iranian Journal of Microbiology 2018; 10(3): 166-70.  9. Akbari Z, Matini M. The study of trichomoniasis in pregnant women attending Hamadan city health centers in 2015. Avicenna journal of clinical microbiology and infection 2017; 4(2): 0-.  10. AL Muathen DM, Sachit HG. Prevalence Trichomoniasis and Candidiasis For Symptomatic Pregnant and Non Pregnant Women in Iraq دراسة انتشار داء المشعرات المهبلية وداء المبيضات لدى النساء الحوامل وغير الحوامل العرضيات في العراق. Al-Mustansiriyah Journal for Pharmaceutical Sciences 2016; 16(2): 21-6.  11. Al-Abodi H, Al-Shaibani K, Shaker E. Molecular investigation of trichomoniasis in women in Al-Muthana province/Iraq. Journal of Physics: Conference Series; 2019: IOP Publishing; 2019. p. 012078.  12. Al-Ammash MSJA-A. Study on prevalence of Trichomomas vaginalis in Samarra city with observation of alcoholic extract effect of Peganum harmala plant in vitro دراسة وبائية لطفيلي المشعرات المهبلية Trichomomas vaginalis في مدينة سامراء مع ملاحظة تحضير المستخلص الكحولي لنبات الحرمل Peganum harmalaفي الزجاج. Tikret Journal of Pharmaceutical Sciences 2017; 12(1): 31-47.  13. Al-Habib HM, Al-Dabbagh NY, Al-Daheen GA. The prevalence of trichomonas vaginalis in association with other micro-organisms among women with vaginal discharge in Mosul. Annals of the College of Medicine Mosul 2005; 31(1): 37-44.  14. Al-Hadraawy Sk, Al-Kafagy SMM, Al-Hadraawy M, K. A. Molecular and immunological study for detection of IL-6 in men infected with Trichomonas vaginalis parasite in Al-Najaf province; Iraq. Al-Qadisiyah Medical Journal 2014; 10(18): 144-8.  15. Al-Hadraawy SK, Al-Katib S, R. , Al-Awady H, M. H. Identification study for suspected women with Trichomonas vaginalis by PCR technique in AL-Najaf AL-Ashraf province دراسة تعريفية للاشخاص المشتبه في اصابتهم بالمهبلات المشعرة بتقنية PCR في محافظة النجف الاشرف. Al-Kufa University Journal for Biology 2018a; 10(1): 183-92.  16. Al-Hadraawy SK, Al-Katib S, R. , Al-Awady H, M. H. Immunological study for infected women with T.vaginalis in AL-Najaf AL-Ashraf province دراسة مناعية للنساء المصابات بمرض T.vaginalis في محافظة النجف الأشرف. Al-Kufa University Journal for Biology 2018b; 10(1): 132-7.  17. Al-Haidari DaMH. Detection of Trichomoniasis in Vaginal and Urine Specimens from Women in Karbala city تشخيص طفيلي المشعرات المهبلية في عينات بولية ومسحات مهبلية من النساء في مدينة كربلاء. Al-Qadisiyah Journal of Pure Science 2011; 16(1): 11-6.  18. Al-Marsomy HD. Association between trichomonas vaginalis and vaginal bacterial community composition in human vagina. Research Journal of Pharmacy and Technology 2020; 13(6): 2925-31.  19. Al-Masoudi HK. Polymerase chain reaction compared with wet mount for detection of Trichomonas vaginalis in women. Int J Pharm Res 2016; 9(3): 240-4.  20. Al-Mousawi JKN, Hashim RT, Manal MKA-Sدجادهطومكا. Microbiological study of bacterial vaginosis among pregnant women in Al-Diwaniya city. The Medical Journal of Basrah University 2006; 24(1&amp;2): 45-9.  21. Al-Muk JM, Hasony HJ. Isolation of Gardnerella vaginalis from pregnant women with bacterial vaginosis in Basrah, Iraq. Bahrain Med Bull 2001; 23(3): 124-6.  22. Al-Omar SL, Al-Kaissi NE, Abdulla FS. Occurrence of HSV with other microorganisms in female genital infection. Al-Mustansiriyah Journal for Pharmaceutical Sciences 2005; 2(1): 1-6.  23. Al-Saeed WM. Detection of Trichomonas vaginalis by different methods in women from Dohok province, Iraq. East Mediterr Health J 2011; 17(9): 706-9.  24. Al-Zuharri OAR, Offi SY, Al-Jubory HTA-k. Genital tract infections among pregnant women التهابات القناة التناسلية بين النساء الحوامل. Al-Kufa University Journal for Biology 2010; 2(1): 207-12.  25. Ali MK, Hathal HD, Almoayed HA. Prevalence and diagnosis of sexually transmitted pathogens in a sample of Iraqi women: a molecular study. Iraqi journal of medical sciences 2017; 15(4): 364-76.  26. Alikhani M, Akhoundi M, Sereno D, et al. Molecular characterization of Trichomonas infections in women of Ilam City, southwestern Iran. Parasitol Res 2022; 121(6): 1631-8.  27. Amina H, Bahija B, Sanae J. Prevalence of bacterial vaginosis in women presenting recurrent vaginal discharge in Morocco. Sexually Transmitted Infections 2017; 93(Supplement 2): A127-A8.  28. Aslam M, Hafeez R, Aman S, Azhar A, Lone DS. Bacterial vaginosis in Pregnant Women: A diagnostic approach. Ann King Edward Med Uni 2004; 10(1): 30-2.  29. Azadi Y, Didarloo H, Ardibazar M, et al. The prevalence of trichomonas vaginalis among pregnant women referred to the tabriz valiasr hospital, Tabriz, Iran. Iranian Journal of Parasitology 2017; 13(1): 58.  30. Azargoon A, Darvishzadeh S. Association of bacterial vaginosis, trichomonas vaginalis, and vaginal acidity with outcome of pregnancy. Arch Iran Med 2006; 9(3): 213-7.  31. Bafghi AF, Aflatoonian A, Barzegar K, Ghafourzadeh M, Nabipour S. Frequency distribution of trichomoniasis in pregnant women referred to health centers of Ardakan, Meibod and Yazd, Iran. Jundishapur Journal of Microbiology 2009; 2(4): 132-9.  32. Bahram A, Hamid B, Zohre T. Prevalence of bacterial vaginosis and impact of genital hygiene practices in non-pregnant women in Zanjan, Iran. Oman Medical Journal 2009; 24(4): 288-93.  33. Bahreini MS, Sedghi S, Badalzadeh Y, et al. Molecular diagnosis of Trichomonas vaginalis in liquid-based Papanicolaou samples in Shiraz, southern Iran. BMC Womens Health 2023; 23(1): 6.  34. Bakhshi A, Safayi Delouyi Z, Taheri S, Alivandi A, Mohammadzadeh N, Dabiri H. Comparative study of lactobacilli and bifidobacteria in vaginal tract of individual with bacterial vaginosis and healthy control by quantitative PCR. Reviews in Medical Microbiology 2019; 30(3): 148-54.  35. Bakhtiari A, Hajian-Tilaki K, Pasha H. Genital infection by Trichomonas Vaginalis in women referring to Babol health centers: Prevalence and risk factors. Iranian Red Crescent Medical Journal 2008; 10(1): 16-21.  36. Beal C, Goldsmith R, Kotby M, et al. The plastic envelope method, a simplified technique for culture diagnosis of trichomoniasis. J Clin Microbiol 1992; 30(9): 2265-8.  37. Bhatti NR, Hayat A, Ahmad S, et al. Prevalance of Vaginal Candiasis in Gynaecology. Pak J Obstet Gynaecol 1995; 8(3): 11-5.  38. Bokharaei-Salim F, Esteghamati A, Khanaliha K, Esghaei M, Donyavi T, Salemi B. The first detection of co-infection of double-stranded rna virus 1, 2 and 3 in iranian isolates of trichomonas vaginalis. Iranian Journal of Parasitology 2020; 15(3): 357-63.  39. Bokharaei-Salim F, Hedayati N, Khanaliha K, et al. Molecular typing of the actin gene of Trichomonas vaginalis isolates in Tehran, Iran. Journal of Parasitic Diseases 2022.  40. Boulos LM, El Temsahy MM, Aly SM, El Agamy ESI, Amer EI. Biological and biochemical studies for characterization of some Egyptian Trichomonas vaginalis isolates. PUJ-Parasitologists United Journal 2012; 5(2): 175-88.  41. Chaloob FA, Abdul-Mohsen AS. Association of Toll-Like Receptor 4 Gene Oolymorphism with Trichomonas vaginalis Infection in Iraqi Women العلاقة بين تعدد الاشكال الجينية لجين Toll-like receptor 4 مع الاصابة بطفيلي المشعرات المهبلية في النساء العراقيات. Medical Journal of Babylon 2014; 11(1): 84-91.  42. Chaudry AE, Chaudhri R, Kayani A, et al. Acceptability and feasibility of screening pregnant women for sexually transmitted infections in Rawalpindi, Pakistan. Int J STD AIDS 2021; 32(10): 940-5.  43. Dalimi A, Payameni S. Trichomonas vaginalis infection in men with high-risk sexual behaviors. Iranian Journal of Parasitology 2021; 16(3): 411-7.  44. Dhumad Hameedi H, Al-Shammari ZAD. Aerobic Bacterial Vaginosis and Lactobacillus Species Associated with Cytomegalovirus in Abortion. Arch Razi Inst 2022; 77(4): 1447-52.  45. Dyab AK, Farouk HA, Mohammed MF, Hassan TM. Parasitological Studies on Trichomonus Vaginalis on Female Patients Presented with Vaginal Discharge at Aswan University Hospital. The Medical Journal of Cairo University 2021; 89(June): 1147-54.  46. El-Gayar E, Mokhtar A, Awad S, Soliman R, Hassan W. The endosymbiotic relationship between Trichomonas vaginalis and Mycoplasma hominis in Egyptian Women and its correlation with pathogenicity. Parasitol United J 2016; 9(2): 80.  47. el-Naga IF, Khalifa AM, el-Azzouni MZ. In-pouch TV culture system in diagnosis of Trichomonas vaginalis infection. J Egypt Soc Parasitol 2001; 31(3): 647-56 + 1p plate.  48. Elsherif HR, Youssef FMA. Real-time PCR improve detection of Trichomonas vaginalis compared to conventional techniques. Comp Clin Pathol 2012; 22(2): 295-300.  49. Ezzat HE. Bacterial vaginosis: diagnosis and associations in vaginal discharge. Medical Journal of Cairo University [The] 1995; 63(4): 997-1005.  50. Farhan RK. Common causes of vaginal infections and antibiotic sensitivity of aerobic bacterial isolates in reproductive age women attending Tikrit teaching hospital, Salah al-Din Governorate, Iraq. NeuroQuantology 2022; 20(6): 7568-83.  51. Farrukh R, Kamal F, Naheed F, Zafar A, Aslam M. Incidence of Bacterial Vaginosis Among Patients with Vaginal Discharge. Ann King Edward Med Uni 2000; 6-4: 391-3.  52. Gabr N, Kamal A, Mohamed R, Abdelwahab S. Sensitivity and specificity of wet mount, culture and PCR in diagnosing trichomonas vaginalis infection in females attending the gynecology clinic of Minia University Hospital. El Minia Medical bulletin 2006; 17(1).  53. Ghallab MMI, Alaa D, Morsy SM. Multiattribute Analysis of Trichomonas vaginalis Diagnostics and Its Correlation with Clinical Complaints and Contraceptive Methods in a Symptomatic Egyptian Cohort. Infect Dis Obstet Gynecol 2021; 2021: 5525095.  54. Ghobahi M, Hamedi Y, Shamseddin J, Heydari Hengami M, Sharifi Sarasiabi K. Frequency of Trichomoniasis and Related Risk Factors in theWomen Referred to Bandar Abbas Health Centers, Iran, 2017-2018. Hormozgan medical journal 2019; 23(1).  55. Haghighi JD, Jafarimodrek M, Sohrabi S, Azizi H, Hatam-Nahavandi K. Trichomoniasis Prevalence at a Care Center Among Women with High-Risk Behaviors in Zahedan, Iran. International Journal of High Risk Behaviors and Addiction 2019; 8(2).  56. Hamdy DA, Hamdy HG. Prevalence, sociodemographic factors and clinical criteria of trichomonas vaginalis infection among symptomatic women in Beni-Suef Governorate, Egypt. Journal of the Egyptian Society of Parasitology 2018; 48(1): 109-17.  57. Hammouda NA, Hegazy IH, Tawfik TA. A rapid diagnostic test for Trichomonas vaginalis infection. J Egypt Soc Parasitol 1997; 27(2): 341-7.  58. Hamouda M, Mohamed S, Elgendy S, Esam Eldeen N, El-Zayady W. Is trichomoniasis associated with adverse preganancy outcome? Parasitol United J 2022; 15(2): 202-9.  59. Hanna J, Yassine R, El-Bikai R, et al. Molecular epidemiology and socio-demographic risk factors of sexually transmitted infections among women in Lebanon. BMC Infect Dis 2020; 20(1): 375.  60. Hassan AAM, Ayoub NM. Comparative study between different methods for diagnosing vaginal trichomoniasis. The new Egyptian Journal of Medicine 1993; 8(3): 636-9.  61. Hassan MF, Rund NMA, El-Tohamy O, et al. Does Aerobic Vaginitis Have Adverse Pregnancy Outcomes? Prospective Observational Study. Infect Dis Obstet Gynecol 2020; 2020: 5842150.  62. Hassan MK, Al-Shaheen H, Al-Mukh JM. Bacterial vaginosis and preterm labour. The Medical Journal of Basrah University 2005; 23(1): 42-6.  63. Hawkes S, Collumbien M, Platt L, et al. HIV and other sexually transmitted infections among men, transgenders and women selling sex in two cities in Pakistan: A cross-sectional prevalence survey. Sexually Transmitted Infections 2009; 85(SUPPL. 2).  64. Hegazy AR, El Kersh WM, Moharm IM, Ammar AI, Hemida AS, Atia AF. Immunological and Cytopathological Assessment of Trichomonas vaginalis Infection in Asymptomatic and Symptomatic Females at Menoufia Governorate, Egypt. Int J Curr Microbiol App Sci 2020; 9(4): 686-705.  65. Hegazy MM, El-Tantawy NL, Soliman MM, El-Sadeek ES, El-Nagar HS. Performance of rapid immunochromatographic assay in the diagnosis of Trichomoniasis vaginalis. Diagn Microbiol Infect Dis 2012; 74(1): 49-53.  66. Heikal EA, Elamir AM, Hegazi MA, et al. Signature of real-time PCR in detection of Trichomonas vaginalis infection and its association with human papillomavirus genotype 16. Eur Rev Med Pharmacol Sci 2023; 27(2): 501-10.  67. Hosny A, El-Khayat W, Kashef MT, Fakhry MN. Association between preterm labor and genitourinary tract infections caused by Trichomonas vaginalis, Mycoplasma hominis, Gram-negative bacilli, and coryneforms. J Chin Med Assoc 2017; 80(9): 575-81.  68. Hussein AH, Saleh MH, Nagaty IM, Ghieth KA, El-Azab NA. Prevalence, clinical criteria and sociodemographic predictors of Trichomonas vaginalis infection in suspected Egyptian women, using direct diagnostic techniques. Iranian Journal of Parasitology 2015; 10(3): 432-40.  69. Ibrahim SS, Ismail MA, Elaskary HM, Khalil EM, Khalil D, Raafat A. Potential Role Of Trichomonas Vaginalis In Women With Primary And Secondary Infertility In Beni-Suef, Egypt. Journal of the Egyptian Society of Parasitology 2021; 51(1): 119-26.  70. Jaafar NK, Kadhum TJ, Ismael I. Study Of Causative Agents Of Cervicitis In Women Attending Gynecologic Outpatient Department In Najaf City دراسة مسببات التهاب عنق الرحم لدى النساء الوافدات إلى قسم النسائية في مستشفيات محافظة النجف. Kufa Medical Journal 2008; 11(1): 166-74.  71. Kadhum NJ, Al-Mayah SH, Raisan SJ. Epidemiological study on trichomonas vaginalis among the women who attended the hospitals of Basra province. Journal of Basrah Researches (Sciences) 2020; 46(2): 64-73.  72. Kadir M, Aziz LJ. A study on trichomonas vaginals infection in Kirkur-Iraq. Bulletin of Endemic Disease-Baghdad 1989; 30: 1-8.  73. Kadir MA, Ghalib AK, Tahir SSH, Al-Dalableh f. A study on trichomonas vaginalis and comparison between the efficacy of metronidazole and secnizole on women in Kirkuk province. Journal of the Faculty of Medicine 2006; 48(1): 94-7.  74. Kalantari N, Ghaffari S, Esmaeilzadeh S. The frequency study of trichomoniasis in women referred to gynecology clinic of Ayatollah Rohani Hospital, Babol, Iran, in 2010. Annals of Tropical Medicine and Public Health 2012; 5(5): 498-501.  75. Kamal AM, Ahmed AK, Mowafy NMES, Shawki HE, Sanad AS, Hassan EE. Incidence of antenatal trichomoniasis and evaluation of its role as a cause of preterm birth in pregnant women referring to Minia University hospital, Egypt. Iranian Journal of Parasitology 2018; 13(1): 58-66.  76. Kareem HK, Hamad MM, Hasan MA, Abd alsammed MA. Study on Trichomonas vaginalis infection in women with type-2 diabetes mellitus and vaginal discharge in Thi-Qar Government. European Journal of Molecular and Clinical Medicine 2020; 7(8): 4471-8.  77. Kermasha ZW, Al-Masoudi HK, Al-Shaikh SF. Association of TLR7 and MyD88 Gene Polymorphism with Trichomoniasis vaginalis Infection. Journal of Contemporary Medical Sciences مجلة العلوم الطبية المعاصرة 2023; 09(04): 304-10.  78. Khalaf A, K. H., Al-Asadi S, A. M., Al-Yaaqub AJ, Al-Mayah SH. Use Pcr Technique To Detect Trichomonas Vaginalis Among Men In Basrah Province استخدام تقنية تفاعل البلمرة التسلسلي PCR في الكشف عن طفيلي المشعرة المهبلية Trichomonas vaginalis بين الرجال في محافظة البصرة. Thi-Qar Medical Journal 2010b; 4(2): 29-36.  79. Khalaf AK, Al Kayat ES. Study the association between the infection with Trichomonas vaginalis and use of contraceptive among women with abnormal vaginal discharge by PCR technique in Nassiriyah city دراسة العلاقة بين التهابات التراكومونس مع استخدام موانع الحمل بين النساء وذوات الافراز المهبلي غير الطبيعي. Thi-Qar Medical Journal 2015; 9(1): 95-101.  80. Khalaf AK, Al-Nasir AHA, Al-Khayat ES. Use PCR technique to detect the infection with Trichomonas vaginalis among women with preterm labor استخدام تقنية تفاعل البلمرة التسلسلي في الكشف عن طفيلي المشهرة المهبلية (Trichomonas vaginalis) بين النساء ذوات الولادة المبكرة. Thi-Qar Medical Journal 2016; 11(1): 156-61.  81. Khalaf AK, Kadhim KJ. Use TVK 3/7 gene as a target to detect Trichomonas vaginalis from urine of women in Southern Iraq استخدام الجين TVK3/7 كمستهدف في الكشف عن طفيلي المشعرة المهبلية Trichomonas vaginalis من إدرار النساء في جنوب العراق. Thi-Qar Medical Journal 2010a; 4(1): 36-46.  82. Khalaf AKH. Detection of Trichomonas vaginalis among women with abnormal vaginal discharge by PCR technique targeting TVK3 and TVK7 genes in Basrah province تشخيص الاصابة بطفيلي المشعرة المهبلية بين النساء اللاتي يعانين من الافراز المهبلي غير الطبيعي في محافظة البصرة باستخدام تقنيةPCR. Thi-Qar Medical Journal 2013; 7(1): 41-8.  83. Khalil HI, Al-Kuraishi AH, Al-Naimi UAM, Al-Naimi SA. Trichomoniasis Vaginalis in Women Attending Family Planning Unit in AL-Liqa'a Hospital داء المشعرات المهبلية لدى النساء المراجعات لوحدة تنظيم الاسرة في مستشفى اللقاء. Iraqi Journal of Science 2012; 53(4): 746-653.  84. Khalili B, Ghasemi-Dehkordi P, Pourshahbazi G, Yousofi-Darani H, Hashemzadeh-Chaleshtori M, Doosti A. Genotyping of Trichomonas vaginalis isolates from women in Shahrekord city (Southwestern Iran). Genetika-Belgrade 2017; 49(3): 1059-70.  85. Khan FZ. Microbial infections in females of childbearing age and therapeutic interventions. Rawal Med J 2011a; 36(3): 178-81.  86. Khan MS, Unemo M, Zaman S, Lundborg CS. HIV, STI prevalence and risk behaviours among women selling sex in Lahore, Pakistan. BMC Infect Dis 2011b; 11: 119.  87. Khaskheli M, Baloch S, Baloch AS. Vaginal discharge during pregnancy and associated adverse maternal and perinatal outcomes. Pak J Med Sci 2021; 37(5): 1302-8.  88. Maghsoudi R, Danesh A, Kabiri N, Setorki M, Doudi M. Prevalence of the genital tract bacterial infections after vaginal reconstructive surgery. Pak J Biol Sci 2014; 17(9): 1058-63.  89. Mahafzah AM, Al-Ramahi MQ, Asa'd AM, El-Khateeb MS. Prevalence of sexually transmitted infections among sexually active Jordanian females. Sex Transm Dis 2008; 35(6): 607-10.  90. Maharlouei N, Barooti E, Sharif F, Hosseini H, Lankarani KB. Prevalence and risk factors of reproductive tract infections among a defined population of Iranian women. Sexual Health 2013; 10(4): 311-5.  91. Mahdey AS, Abd FG. Molecular Detection the Agent that Causing Vaginitis in Vaginal Secretion from Women with Vaginitis and it Relation with Abortion. Journal of University of Babylon 2018; 26(6): 253-62.  92. Mahdi NK. Urogenital trichomoniasis in an Iraqi population. Eastern Mediterranean Health Journal 1996; 2(3).  93. Mahdi NK, Gany ZH, Sharief M. Risk factors for vaginal trichomoniasis among women in Basra, Iraq. East Mediterr Health J 2001; 7(6): 918-24.  94. Mahmoud A, Sherif NA, Abdella R, El-Genedy AR, El Kateb AY, Askalani AN. Prevalence of Trichomonas vaginalis infection among Egyptian women using culture and Latex agglutination: cross-sectional study. BMC Womens Health 2015; 15: 7.  95. Manshoori A, Mirzaei S, Valadkhani Z, et al. A diagnostic and symptomatological study on trichomoniasis in symptomatic pregnant women in Rafsanjan, south central Iran in 2012-13. Iranian Journal of Parasitology 2015; 10(3): 490-7.  96. Maraghi S, Khosravi A, Kardooni T, Razi T, Feiz-Haddad MH. Evaluation of an Immunochromatographic Strip (Xenostrip - Tv) Test for Diagnosis of Vaginal Trichomoniasis Compared with Wet Mount and PCR Assay. Iranian Journal of Parasitology 2008; 3(3): 11-7.  97. Matini M, Rezaei H, Fallah M, Maghsood AH, Saidijam M, Shamsi-Ehsan T. Genotyping, drug susceptibility and prevalence survey of Trichomonas vaginalis among women attending gynecology clinics in Hamadan, Western Iran, in 2014-2015. Iranian Journal of Parasitology 2017; 12(1): 29-37.  98. Matini M, Rezaeian M, Mohebali M, et al. Genotyping of Trichomonas vaginalis isolates in Iran by using single stranded conformational polymorphism-PCR technique and internal transcribed spacer regions. Trop Biomed 2012; 29(4): 605-12.  99. Mazloumi Gavgani AAS, Namazi A, Ghazanchaei A, et al. Prevalence and risk factors of trichomoniasis among women in Tabriz. Iranian journal of clinical infectious diseases 2008; 3(2): 67-71.  100. Ministry of Health - Morocco. HIV integrated behavioral and biological surveillance surveys - Morocco 2011, 2011.  101. Moaiedmohseni S, Bashardoost L, Abbasi M. Cervicovaginal infections during third trimester of pregnancy. Journal Of Family and Reproductive Health 2012: 11-5.  102. Mohamad AN, Eskandar AY, Laftah AA. Prevalence of trichomoniasis in baquba city. Al-Mustansiriyah Journal of Science 2007; 18(3): 19-23.  103. Mushref E, Ardalan NM, Ahmed ZaA-R. Trichomonas vaginalis used as a marker for other sexually transmitted infections in women إمكانية استخدام طفيلي المشعرات المهبليه كدليل لأصابات جنسية اخرى عند النساء. Iraqi Journal of Community Medicine 2010; 23(4): 292-4.  104. Naama JK, Hasson KF, Abdullah EE. Detection of Trichomonas Vaginals among women with contraceptive usage in AL-Najaf AL-ashraf city أنتشار داء المشعرات في النساء المستخدمة موانع الحمل في النجف الأشرف. Thi-Qar Medical Journal 2008; 2(1): 46-50.  105. Nasir JA, J N, F T, Asghar N, Iqbal J. Trichomonas vaginalis in vaginal smears of women using intrauterine contraceptive device. Pak J Med Res 2005; 44(3): 114-6.  106. Nasir MAH, S.R A-I, Al-Masoudi WA. Comparison of Different Techniques for the Diagnosis of Trichomonas Vaginalis Infection in Females at Reproductive Age. Pakistan Journal of Medical and Health Sciences 2022; 16(7): 451-4.  107. Nassef NE, Afif AF, Basuni AA, El-Nasr MFA, Atia AF. Evaluation of microscopy and polymerase chain reaction for diagnosis of symptomatic and asymptomatic female trichomoniasis. Parasitol United J 2014; 7(1): 37.  108. Nateghi Rostami M, Hossein Rashidi B, Nazari R, Aghsaghloo F, Habibi A. A multiplex assay of Trichomonas vaginalis, Chlamydia trachomatis and Neisseria gonorrhoeae infections in genital specimens. J Infect Dev Ctries 2017; 11(11): 833-9.  109. Nazari N, Zangeneh M, Moradi F, Bozorgomid A. Prevalence of trichomoniasis among women in Kermanshah, Iran. Iranian Red Crescent Medical Journal 2015; 17(3): 1-4.  110. Nouraddin AS, Alsakee HM. Prevalence of Trichomonas vaginalis infection among women in Erbil governorate, Northern Iraq: An epidemiological approach. European Scientific Journal 2015; 11(24): 243-55.  111. Nourian A, Shabani N, Fazaeli A, Mousavinasab SN. Prevalence of Trichomonas vaginalis in pregnant women in Zanjan, Northwest of Iran. Jundishapur Journal of Microbiology 2013; 6(8): e7258.  112. Omer EF, el-Naeem HA, Ali MH, Catterall RD, Erwa HH. Evaluation of the laboratory diagnosis of vaginal trichomoniasis in Khartoum. J Trop Med Hyg 1988; 91(6): 292-5.  113. Payamani S, Dalimi A, Gaffarifar F. A study on trichomoniasis in HIV positive women in Tehran, Iran. Iranian Journal of Parasitology 2017; 13(1): 92.  114. Rabiee S, Fallah M, Zahabi F. Frequency of trichomoniasis in patients admitted to outpatient clinics in Hamadan (2007) and relationship between clinical diagnosis and laboratory findings. Journal of Research in Health Sciences 2010; 10(1): 31-5.  115. Rajabpour M, Emamie AD, Pourmand MR, Goodarzi NN, Asbagh FA, Whiley DM. Chlamydia trachomatis, Neisseria gonorrhoeae, and Trichomonas vaginalis among women with genitourinary infection and pregnancy-related complications in Tehran: A cross-sectional study. Int J STD AIDS 2020; 31(8): 773-80.  116. Ramia S, Kobeissi L, El Kak F, Shamra S, Kreidieh K, Zurayk H. Reproductive tract infections (RTIs) among married non-pregnant women living in a low-income suburb of Beirut, Lebanon. J Infect Dev Ctries 2012; 6(9): 680-3.  117. Rezaeian M, Vatanshenassan M, Rezaie S, et al. Prevalence of Trichomonas vaginalis using parasitological methods in Tehran. Iranian Journal of Parasitology 2009; 4(4): 43-7.  118. Rizvi TH, Hassan FH, Surryia S, Sha SSA. Vaginal infection and birth weight. Pak J Med Res 2003; 42(1): 7-9.  119. Rostami MN, Rashidi BH, Habibi A, Nazari R, Dolati M. Genital infections and reproductive complications associated with trichomonas vaginalis, Neisseria gonorrhoeae, and Streptococcus agalactiae in women of Qom, central Iran. International Journal of Reproductive BioMedicine 2017; 15(6): 357-66.  120. Ryan CA, Zidouh A, Manhart LE, et al. Reproductive tract infections in primary healthcare, family planning, and dermatovenereology clinics: Evaluation of syndromic management in Morocco. Sexually Transmitted Infections 1998; 74(SUPPL. 1): S95-S105.  121. Saba Fadhil A. Incidence of trichomonus vaginalis infection after menopause age of womenحدوث الإصابة بالمشعرات المهبلية بعد سن اليأس في النساء. Journal of Techniques 2012; 25(2): 16-20.  122. Saber N, Saraei M, Hajialilo E, et al. Screening and molecular characterization of Trichomonas vaginalis genotypes isolated from married women in northern Iran. Ann Parasitol 2022; 68(3): 587-94.  123. Saleh AM, Abdalla HS, Satti AB, Babiker SM, Gasim GI, Adam I. Diagnosis of Trichomonous vaginalis by microscopy, latex agglutination, diamond's media, and PCR in symptomatic women, Khartoum, Sudan. Diagn Pathol 2014; 9: 49.  124. Salih AA, Obaid HM, Jasim W, M. . An in vitro study of zinc effect on Trichomonas vaginalis isolated from infected women. NTU Journal of Pure Sciences 2022; 1(4): 44-52.  125. Sallam MA, El Sharkawy EM. Trichomonas vaginalis is not a rare sexually-transmitted disease among Egyptian men. Scientific Journal of Al-Azhar Medical Faculty [Girls] [The] 2003; 24(1): 373-80.  126. Sallam S, Ali OT, Hassan MN, Fares E. Epidemiology of gonorrhea among married females presenting with leucorrhoea. Bulletin of High Institute of Public Health 1982; 12(3): 65-80.  127. Salman ST, Hussein AA. Contraception as a Risk Factor of Trichomonas vaginalis Infection Among Women Attending Outpatient of Al-Batool Teaching Hospital for Maternity and Children-Baqubah-Iraq وسائل منع الحمل كعامل خطورة للاصابة بداء المشعرات المهبلية للنساء اللواتي يراجعن العيادة الخارجية لمستشفى البتول التعليمي للأمومة والطفولة في ديالى/ العراق. Al-Kindy College Medical Journal 2017; 13(1): 20-6.  128. Shahnazi E, Mohammadzadeh H, Daneshyar C, Chavshin A, Khademvatan S. Frequency and molecular diagnosis of trichomoniasis in symptomatic women referred to laboratories in urmia north west Iran. Journal of Acute Disease 2017; 6(4): 175-80.  129. Shahraki F, Fouladi B, Salimi-Khorashad A, Sepehri-Rad N, Dabirzadeh M. Epidemiology and identification of actin gene of Trichomonas vaginalis genotypes in women of southeast of Iran using PCR-RFLP. Crescent Journal of Medical and Biological Sciences 2020; 7(1): 82-90.  130. Shaker EM, Almaeahi AMY, Al-Shaibani KTM. Epidemiological study of trichomoniasis with the effect of estrogen hormones lh and fsh among married women in samarra city, iraq. International Journal of Research in Pharmaceutical Sciences 2019; 10(1): 372-7.  131. Shawaky SM, Al Shammari MMA, Sewelliam MS, Ghazal A, Amer AN. A study on vaginitis among pregnant and non-pregnant females in Alexandria, Egypt: An unexpected high rate of mixed vaginal infection. AIMS Microbiol 2022; 8(2): 167-77.  132. Sheetawy AZ, Abdulla ZA. Detection of chlamydia and other bacteria in cervicitis. Annals of the College of Medicine Mosul 2007; 33(1&amp;2): 26-34.  133. Shipitsyna E, Kularatne R, Golparian D, et al. Mycoplasma genitalium prevalence, antimicrobial resistance-associated mutations, and coinfections with non-viral sexually transmitted infections in high-risk populations in Guatemala, Malta, Morocco, Peru and South Africa, 2019-2021. Frontiers in Microbiology 2023; 14: 1130762.  134. Shobeiri F, Nazari M. A prospective study of genital infections in Hamedan, Iran. Southeast Asian J Trop Med Public Health 2006; 37 Suppl 3: 174-7.  135. Taher JH. Epidemiological and Biological Variability in Clinical Isolates of Trichomonasvaginalis among Women in Najaf/ Iraq, الوبائية والتغايرات الحيوية لعزلات سريرية لطفيلي المشعرة المهبلية لدى النساء في محافظة النجف / العراق. Kerbala journal of pharmaceutical sciences 2012; (3): 23-33.  136. Taher JHT, Shaker M, A. Epidemiological Study of Trichomonas vaginalis and Other Microorganisms Isolated from Genital Tract of Women in Najaf Province – Iraq دراسة وبائية المشعرة المهبلية والأحياء المجهريه الاخرى المعزولة من القناة التناسليه للنساء في محافظة النجف / العراق. Al-Kufa University Journal for Biology 2018; 10(2): 1-9.  137. Valadkhani Z, Assmar M, Hassan N, et al. The prevalence of trichomoniasis in high-risk behavior women attending the clinics of tehran province penitentiaries. Iranian Journal of Medical Sciences 2010; 35(3): 190-4.  138. Valadkhani Z, Safaee Z, Sohrabi M. Prevalence of Trichomonas vaginalis infection among Iranian women using P270 gene. Tropical Medicine and International Health 2015; 20(SUPPL. 1): 284-5.  139. Valadkhani ZT, Asmar M, Esfandiari B, et al. Trichomoniasis in Asymptomatic Patients. Iranian journal of public health 2008; 37(3): 113-7.  140. Wijdan Dhaidan Shnain Al- Abbas وجدان ض, Ohood Aqeed Radhi عهود ع. Incidence of Chlamydia trachomatis and Trichomonas Vaginalis Genital Infections among Non-Pregnant Women in Al - Najaf Province الإصابة بالكلاميديا الحثرية والتريكوموناس المهبلية التناسلية لدى النساء غير الحوامل في محافظة النجف. kufa Journal for Nursing sciences 2019; 9(1): 1-8.  141. Yarizadeh M, Taherkhani H, Amir-Zargar MA, Matini M. Molecular epidemiologic study of male trichomoniasis in hamadan, western iran. Iranian Journal of Parasitology 2021; 16(2): 245-52.  142. Yaseen SAS. Study About the Causative Agents of Cervical Infections and Cytopathological Changes in Iraqi Women. Iraqi Journal of Science 2020; 61(2): 246-53.  143. Younis H, Al Tae AR, Khaki II. Comparative study for the diagnosis of trichomonas vaginalis. IPMJ-Iraqi Postgraduate Medical Journal 2003; 2(4): 394-8.  144. Yousofi Darani H, Ahmadi F, Zebardast N, Yousefi HA, Shirzad H. Development of a Latex Agglutination Test as a Simple and Rapid Method for Diagnosis of Trichomonas vaginalis Infection. Avicenna Journal of Medical Biotechnology (AJMB) 2010; 2(1): 63-6.  145. Zaki ESM, Raafat D, El Emshaty W, Azab MS, Goda H. Correlation of Trichomonas vaginalis to bacterial vaginosis: a laboratory-based study. J Infect Dev Ctries 2010; 4(3): 156-63.  146. Zaki MM, Moussa HM, Hassanin OM. Evaluation of the OSOM Trichomonas rapid test for detection of Trichomoniasis vaginalis. PUJ-Parasitologists United Journal 2011; 4(2): 177-84.  147. Zarandi MB, Fard SRN, Parastouei K, Ahmadi A. Causative Agents of Vaginitis in Women of Kerman Province, Iran. Journal of medical microbiology and infectious diseases 2019; 7(1-2): 29-31.  148. Zribi M, Ben Mansour K, Abid F, Masmoudi A, Fendri C. Syndromic approach to sexually transmitted infections in Tunisian women: Bacteriological validation. International Journal of STD and AIDS 2008; 19(2): 112-4. |
| --- |

# **Table S4.** Summary of precision and risk of bias assessments for studies reporting *Trichomonas vaginalis* prevalence in the Middle East and North Africa.

| **Precision and risk of bias assessments** | ***Trichomonas vaginalis* prevalence measures** | |
| --- | --- | --- |
|  | **Number of studies** | **%** |
| **Precision of prevalence measures^a^** | | |
| Low precision | 203 | 43.9 |
| High precision | 259 | 56.1 |
| **Risk of bias quality domain^b^** | | |
| **Sampling method** | | |
| Low risk of bias | 43 | 9.3 |
| High risk of bias | 419 | 90.7 |
| **Response rate** | | |
| Low risk of bias | 16 | 3.5 |
| High risk of bias | 3 | 0.6 |
| Unclear risk of bias | 443 | 95.9 |
| **Summary of the risk of bias assessment** | | |
| **Low risk of bias** |  |  |
| In at least one quality domain | 50 | 10.8 |
| In both quality domains | 9 | 1.9 |
| **High risk of bias** |  |  |
| In at least one quality domain | 419 | 90.7 |
| In both quality domains | 3 | 0.6 |
| **Prevalence studies where risk of bias assessment was possible** | **462** | **100** |

^a^ Precision was assessed based on the overall sample size (not each stratum subsample size) of the study as reported in the report/publication.

^b^ Risk of bias was assessed based on the overall sample size (not each stratum subsample size) of the study as reported in the report/publication.

# **Table S5.** Assessment of publication bias using Doi plots and the LFK index.

| **Population type** | **Outcome measures** | **LFK index** | **Doi plot inspection** | **Interpretation** |
| --- | --- | --- | --- | --- |
|  | **Total n** |  |  |  |
| General populations | 266 | 0.81 | Symmetrical Doi plot | No publication bias |
| Intermediate risk populations | 10 | 3.62 | Asymmetrical Doi plot | Indicative of publication bias |
| Female sex workers | 14 | -0.59 | Symmetrical Doi plot | No publication bias |
| Symptomatic women | 323 | 7.23 | Asymmetrical Doi plot | Indicative of publication bias |
| Infertility clinic attendees | 7 | 0.23 | Symmetrical Doi plot | No publication bias |
| Women with miscarriages or ectopic pregnancies | 7 | 0.42 | Symmetrical Doi plot | No publication bias |
| STI clinic attendees | 8 | 2.98 | Asymmetrical Doi plot | Indicative of publication bias |
| Other populations | 47 | 6.13 | Asymmetrical Doi plot | Indicative of publication bias |

Abbreviations: LFK = Luis Furuya-Kanamori, STI = Sexually transmitted infection.

# **Table S6.** Results of meta-analyses for *Trichomonas vaginalis* prevalence in the Middle East and North Africa, utilizing exclusively prevalence measures that met the stringent inclusion criteria for diagnostic methods. The results encompass diverse populations and are stratified by assay type.

| **Population type** | **Outcome measures** | **Sample size** | **TV prevalence (%)** | | **Pooled mean TV prevalence** | **Heterogeneity measures** | | | |
| --- | --- | --- | --- | --- | --- | --- | --- | --- | --- |
|  |  |  |  |  |  |  |  |  |  |
|  | **Total** | **Total** | **IQR** | **Median** | **Mean (%)** | **Q^a^** | | **I²**^b^ **(%)** | **Prediction interval**^c^ **(%)** |
|  | **n** | **N** |  |  | **(95% CI)** | **(p-value)** | | **(95% CI)** |  |
| **General populations** |  |  |  |  |  |  | |  |  |
| NAAT/PCR | 24 | 6,659 | 0.6-8.5 | 2.0 | 4.1 (1.7-7.3) | 316.4 (p<0.001) | | 92.7 (90.4-94.5) | 0.0-27.8 |
| Culture | 41 | 8,838 | 1.6-11.2 | 5.0 | 6.1 (3.9-8.8) | 478.1 (p<0.001) | | 91.6 (89.6-93.3) | 0.0-28.9 |
| Wet mount | 69 | 49,715 | 1.9-10.0 | 4.6 | 6.2 (4.1-8.6) | 2,632.9 (p<0.001) | | 97.4 (97.1-97.7) | 0.0-34.8 |
| **Overall** | **134** | **65,212** | **1.4-10.0** | **4.5** | **5.7 (4.3-7.2)** | **3,893.8 (p<0.001)** | | **96.6 (96.3-96.9)** | **0.0-30.3** |
| **Intermediate risk populations** |  |  |  |  |  |  | |  |  |
| NAAT/PCR | 2 | 47 | 64.9-66.1 | 65.5 | 66.0 (50.7-73.1)^d^ | - | | - | - |
| Culture | 1 | 450 | - | - | 10.2 (7.6-13.4) | - | | - | - |
| Wet mount | 1 | 450 | - | - | 9.1 (6.6-12.2) | - | | - | - |
| **Overall** | **4** | **947** | **9.9-64.9** | **37.3** | **32.6 (5.7-67.8)** | **71.3 (p<0.001)** | | **95.8 (92.0-97.8)** | **0.0-100.0** |
| **Female sex workers** |  |  |  |  |  |  | |  |  |
| NAAT/PCR | 5 | 1,433 | 5.7-14.9 | 11.8 | 12.8 (3.7-26.1) | 215.1 (p<0.001) | | 98.1 (97.1-98.8) | 0.0-74.0 |
| Culture | 3 | 730 | 2.1-7.1 | 2.9 | 4.3 (0.5-11.3) | 26.7 (p<0.001) | | 92.5 (81.3-97.0) | 0.0-100.0 |
| **Overall** | **8** | **2,163** | **4.0-12.6** | **8.5** | **9.1 (3.2-17.5)** | **298.0 (p<0.001)** | | **97.7 (96.7-98.4)** | **0.0-48.1** |
| **Symptomatic women** |  |  |  |  |  |  | |  |  |
| NAAT/PCR | 37 | 5,811 | 13.1-32.0 | 21.5 | 23.7 (17.7-30.2) | 1,451.7 (p<0.001) | | 97.5 (97.1-97.9) | 0.0-66.8 |
| Culture | 75 | 18,016 | 5.5-19.6 | 10.0 | 13.1 (9.9-16.6) | 1,729.2 (p<0.001) | | 95.7 (95.1-96.3) | 0.0-49.9 |
| Wet mount | 92 | 18,382 | 5.2-19.6 | 11.5 | 12.5 (9.7-15.5) | 2,589.6 (p<0.001) | | 96.5 (96.1-96.9) | 0.0-47.2 |
| Rapid test | 4 | 528 | 11.9-14.0 | 13.3 | 12.4 (9.7-15.4) | 1.1 (p=0.78) | | 0.0 (0.0-84.7) | 6.9-19.3 |
| **Overall** | **208** | **42,737** | **6.4-22.1** | **12.3** | **14.5 (12.4-16.7)** | **6,621.5 (p<0.001)** | | **96.7 (96.4-96.9)** | **0.0-51.8** |
| **Symptomatic men** |  |  |  |  |  |  | |  |  |
| NAAT/PCR | 2 | 175 | 16.9-17.4 | 17.2 | 17.1 (11.9-23.6)^d^ | - | | - | - |
| Wet mount | 1 | 90 | - | - | 10.0 (4.7-18.1) | - | | - | - |
| **Overall** | **3** | **265** | **13.4-17.2** | **16.7** | **14.6 (10.0-19.8)** | **2.6 (p=0.28)** | | **21.8 (0.0-91.9)** | **0.0-67.8** |
| **Infertility clinic attendees** |  |  |  |  |  |  | |  |  |
| NAAT/PCR | 2 | 238 | 3.3-6.6 | 5.0 | 3.4 (1.5-6.5)^d^ | - | | - | - |
| Culture | 1 | 90 | - | - | 10.0 (4.7-18.1) | - | | - | - |
| Wet mount | 2 | 150 | 2.9-5.4 | 4.2 | 4.7 (1.9-9.4)^d^ | - | | - | - |
| **Overall** | **5** | **478** | **1.7-8.3** | **6.7** | **5.0 (1.9-9.1)** | **12.5 (p=0.01)** | | **68.1 (17.6-87.6)** | **0.0-22.7** |
| **Women with miscarriages or ectopic pregnancies** | | |  |  |  |  | |  |  |
| NAAT/PCR | 3 | 289 | 1.4-4.2 | 2.8 | 1.6 (0.0-6.3) | 6.7 (p=0.036) | | 70.0 (0.0-91.0) | 0.0-99.4 |
| Wet mount | 1 | 60 | - | - | 6.7 (1.8-16.2) | - | | - | - |
| **Overall** | **4** | **349** | **2.1-5.8** | **4.2** | **2.6 (0.0-7.3)** | **10.4 (p=0.016)** | | **71.1 (17.4-89.9)** | **0.0-34.0** |
| **STI clinic attendees** |  |  |  |  |  |  | |  |  |
| NAAT/PCR | 1 | 294 | - | - | 3.1 (1.4-5.7) | - | | - | - |
| Culture | 1 | 500 | - | - | 3.2 (1.8-5.1) | - | | - | - |
| Wet mount | 1 | 500 | - | - | 2.6 (1.4-4.4) | - | | - | - |
| **Overall** | **3** | **1,294** | **2.8-3.1** | **3.1** | **2.9 (2.1-3.9)** | **0.4 (p=0.84)** | | **0.0 (0.0-89.6)** | **0.0-11.8** |
| **HIV-positive individuals and individuals in HIV-discordant couples** | | | |  |  |  | |  |  |
| Culture | 1 | 50 | - | - | 60.0 (45.2-73.6) | - | | - | - |
| **Overall** | **1** | **50** | **-** | **-** | **60.0 (45.2-73.6)** | **-** | | **-** | **-** |
| **Other populations^e^** |  |  |  |  |  |  | |  |  |
| NAAT/PCR | 4 | 869 | 4.7-4.8 | 4.8 | 4.2 (2.9-5.7) | 0.4 (p=0.94) | | 0.0 (0.0-84.7) | 1.7-7.8 |
| Culture | 14 | 4,972 | 3.2-8.5 | 5.5 | 6.8 (2.8-12.3) | 482.3 (p<0.001) | | 97.3 (96.5-97.9) | 0.0-36.1 |
| Wet mount | 12 | 5,511 | 3.8-5.8 | 5.3 | 5.9 (2.5-10.6) | 341.0 (p<0.001) | | 93.8 (95.6-97.6) | 0.0-30.0 |
| **Overall** | **30** | **11,352** | **3.4-7.8** | **4.9** | **6.1 (3.7-9.0)** | **834.4 (p<0.001)** | **96.5 (95.8-97.1)** | | **0.0-27.3** |

Abbreviation: CI = Confidence interval, IQR = Interquartile range, HIV = Human immunodeficiency virus, NAAT = Nucleic acid amplification test, PCR = Polymerase chain reaction, STI = Sexually transmitted infection, TV = *Trichomonas vaginalis*.

A minimum of three studies was required to perform a meta-analysis.

^a^ Q: The Cochran's Q statistic is a measure assessing the existence of heterogeneity in pooled outcome measures, here TV prevalence.

^b^ I^2^: A measure that assesses the magnitude of between-study variation that is due to true differences in TV prevalence across studies rather than chance.

^c^ Prediction interval: A measure that estimates the distribution (95% interval) of true TV prevalence around the estimated mean.

^d^ Two prevalence measures are not sufficient to conduct a random-effects meta-analysis. The pooled measure was calculated as the arithmetic mean of the two measures and their 95% confidence intervals.

^e^ Other populations include populations with an undetermined risk of acquiring TV infection such as women with premature labor, cancer patients, patients suffering from diabetes, and mixed at-risk populations, among others.

# **Table S7.** Sensitivity analysis. Results of meta-analyses for *Trichomonas vaginalis* prevalence in the Middle East and North Africa using the Hartung-Knapp-Sidik-Jonkman method.^6-8^

| **Population type** | **Outcome measures** | **Sample size** | **TV prevalence (%)** | | **Pooled mean TV prevalence^a^** | **Heterogeneity measures** | | |
| --- | --- | --- | --- | --- | --- | --- | --- | --- |
|  |  |  |  |  |  |  |  |  |
|  | **Total** | **Total** | **IQR** | **Median** | **Mean (%)** | **Q^b^** | **I²^c^ (%)** | **Prediction interval^d^ (%)** |
|  | **n** | **N** |  |  | **(95% CI)** | **(p-value)** | **(95% CI)** |  |
|  | **Prevalence measures that met the standard inclusion criteria for diagnostic methods** | | | | | | | |
| **Intermediate risk populations** | |  |  |  |  |  |  |  |
| NAAT/PCR | 4 | 847 | 0.0-66.7 | 32.5 | 8.0 (0.0-97.1) | 89.5 (p<0.001) | 96.6 (93.9-98.2) | 0.0-100.0 |
| Culture | 3 | 553 | 10.2-28.0 | 18.9 | 17.2 (4.1-50.1) | 13.8 (p=0.001) | 85.5 (57.5-95.1) | 0.0-99.1 |
| Wet mount | 3 | 960 | 7.8-26.5 | 9.1 | 12.7 (2.0-50.9) | 36.9 (p<0.001) | 94.6 (87.5-97.6) | 0.0-100.0 |
| **Female sex workers** | |  |  |  |  |  |  |  |
| Culture | 5 | 1,621 | 2.9-11.6 | 11.2 | 6.4 (1.8-20.7) | 32.4 (p<0.001) | 87.7 (73.7-94.2) | 0.2-71.9 |
| **Symptomatic women** | |  |  |  |  |  |  |  |
| Rapid test | 5 | 650 | 10.2-14.0 | 12.5 | 11.4 (7.7-16.7) | 5.4 (p=0.25) | 26.3 (0.0-70.7) | 5.3-22.9 |
| **Symptomatic men** | |  |  |  |  |  |  |  |
| Overall | 3 | 265 | 10.0-17.6 | 16.7 | 14.7 (7.6-26.7) | 2.6 (p=0.28) | 16.4 (0.0-91.3) | 1.9-61.0 |
| **Symptomatic patients (mixed sexes)** | | |  |  |  |  |  |  |
| NAAT/PCR | 3 | 108 | 0.0-9.8 | 7.1 | 7.5 (1.4-32.3) | 2.0 (p=0.37) | 0.3 (0.0-89.6) | 0.0-94.1 |
| Overall | 3 | 108 | 0.0-9.8 | 7.1 | 7.5 (1.4-32.3) | 2.0 (p=0.37) | 0.3 (0.0-89.6) | 0.0-94.2 |
| **Infertility clinic attendees** | | |  |  |  |  |  |  |
| Culture | 3 | 287 | 1.8-33.1 | 10.0 | 10.9 (0.2-86.2) | 22.9 (p<0.001) | 91.3 (77.4-96.6) | 0.0-100.0 |
| **Women with miscarriages or ectopic pregnancies** | | |  |  |  |  |  |  |
| NAAT/PCR | 4 | 319 | 0.0-5.6 | 1.4 | 2.9 (0.9-8.7) | 2.8 (p=0.43) | 0.0 (0.0-84.7) | 0.6-13.3 |
| **STI clinic attendees** | |  |  |  |  |  |  |  |
| NAAT/PCR | 5 | 2,891 | 0.4-3.1 | 1.2 | 1.1 (0.4-2.9) | 16.0 (p=0.003) | 74.9 (38.2-89.8) | 0.1-10.3 |
| **Other populations^e^** | |  |  |  |  |  |  |  |
| NAAT/PCR | 5 | 1,319 | 4.8-4.9 | 4.8 | 5.6 (3.7-8.2) | 6.6 9 (p=0.16) | 39.0 (0.0-77.4) | 2.9-10.5 |
|  | **Prevalence measures that met the stringent inclusion criteria for diagnostic methods** | | | | | | | |
| **Intermediate risk populations** | |  |  |  |  |  |  |  |
| Overall | 4 | 947 | 9.9-64.9 | 37.3 | 30.0 (3.0-85.8) | 78.2 (p<0.001) | 29.2 (92.9-97.9) | 0.0-99.9 |
| **Female sex workers** | |  |  |  |  |  |  |  |
| NAAT/PCR | 5 | 1,433 | 5.7-14.9 | 11.8 | 11.2 (3.1-32.8) | 183.8 (p<0.001) | 97.8 (96.6-98.6) | 0.3-83.8 |
| Culture | 3 | 730 | 2.1-7.1 | 2.9 | 3.7 (0.2-42.1) | 21.9 (p<0.001) | 90.9 (76.2-96.5) | 0.0-100.0 |
| **Symptomatic women** | |  |  |  |  |  |  |  |
| Rapid test | 4 | 528 | 11.9-14.0 | 13.3 | 12.6 (10.1-15.6) | 1.1 (p=0.79) | 0.0 (0.0-84.7) | 7.6-20.2 |
| **Symptomatic men** | |  |  |  |  |  |  |  |
| Overall | 3 | 265 | 13.4-17.2 | 16.7 | 15.0 (7.1-28.9) | 2.4 (p=0.30) | 16.4 (0.0-91.3) | 0.8-79.4 |
| **Infertility clinic attendees** | |  |  |  |  |  |  |  |
| Overall | 5 | 478 | 1.7-8.3 | 6.7 | 5.3 (1.9-14.0) | 9.9 (p=0.043) | 59.4 (0.0-84.8) | 0.5-39.7 |
| **Women with miscarriages or ectopic pregnancies** | | |  |  |  |  |  |  |
| NAAT/PCR | 3 | 289 | 1.4-4.2 | 2.8 | 2.8 (0.3-24.6) | 2.6 (p=0.27) | 23.8 (0.0-92.1) | 0.0-99.9 |
| Overall | 4 | 349 | 2.1-5.8 | 4.2 | 3.8 (1.0-13.8) | 4.3 (p=0.23) | 30.2 (0.0-74.6) | 0.2-45.3 |
| **STI clinic attendees** | |  |  |  |  |  |  |  |
| Overall | 3 | 1,294 | 2.8-3.1 | 3.1 | 3.0 (2.2-3.9) | 0.3 (p=0.85) | 0.0 (0.0-89.6) | 0.4-19.8 |
| **Other populations^e^** | |  |  |  |  |  |  |  |
| NAAT/PCR | 4 | 869 | 4.7-4.8 | 4.8 | 4.4 (3.9-5.0) | 0.2 (p=0.98) | 0.0 (90.0-84.7) | 2.2-8.6 |

Abbreviation: CI = Confidence interval, IQR =Interquartile range, NAAT = Nucleic acid amplification test, PCR = Polymerase chain reaction, STI = Sexually transmitted infection, TV = *Trichomonas vaginalis*.

^a^ Pooled mean prevalence was calculated using the Hartung-Knapp-Sidik-Jonkman method^6-8^ for cases where the number of studies involved in the meta-analysis was ≤5.

^b^ Q: The Cochran's Q statistic is a measure assessing the existence of heterogeneity in pooled outcome measures, here TV prevalence.

^c^ I^2^: A measure that assesses the magnitude of between-study variation that is due to true differences in TV prevalence across studies rather than chance.

^d^ Prediction interval: A measure that estimates the distribution (95% interval) of true TV prevalence around the estimated mean.

^e^ Other populations include populations with an undetermined risk of acquiring TV infection such as women with premature labor, cancer patients, patients suffering from diabetes, and mixed at-risk populations, among others.

# **Figure S1.** Forest plots displaying the pooled mean *Trichomonas vaginalis* prevalence among diverse populations in the Middle East and North Africa.

1.
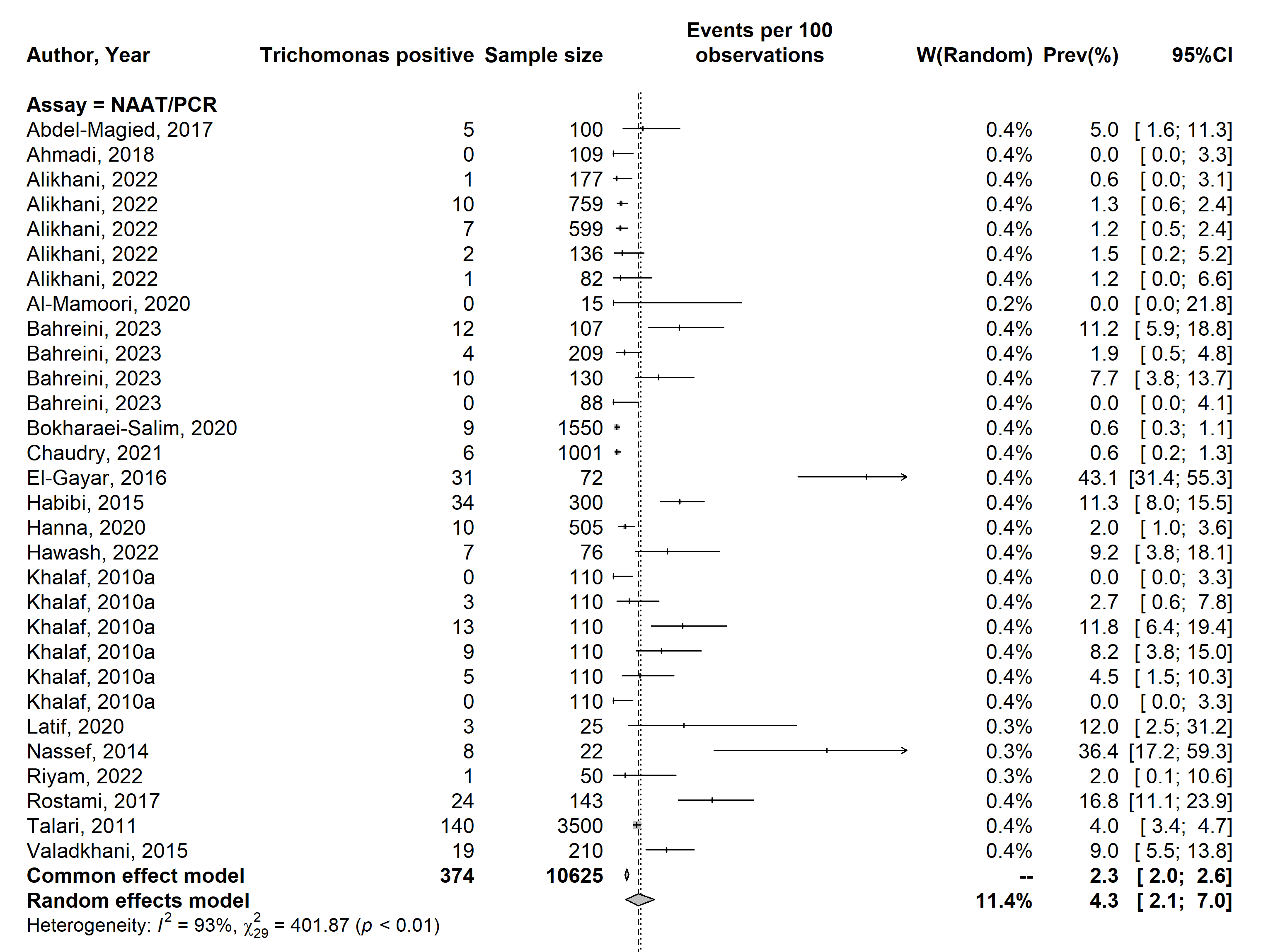
General populations tested using NAAT/PCR

Abbreviations: CI = Confidence interval, NAAT = Nucleic acid amplification test, PCR = Polymerase chain reaction.

1.
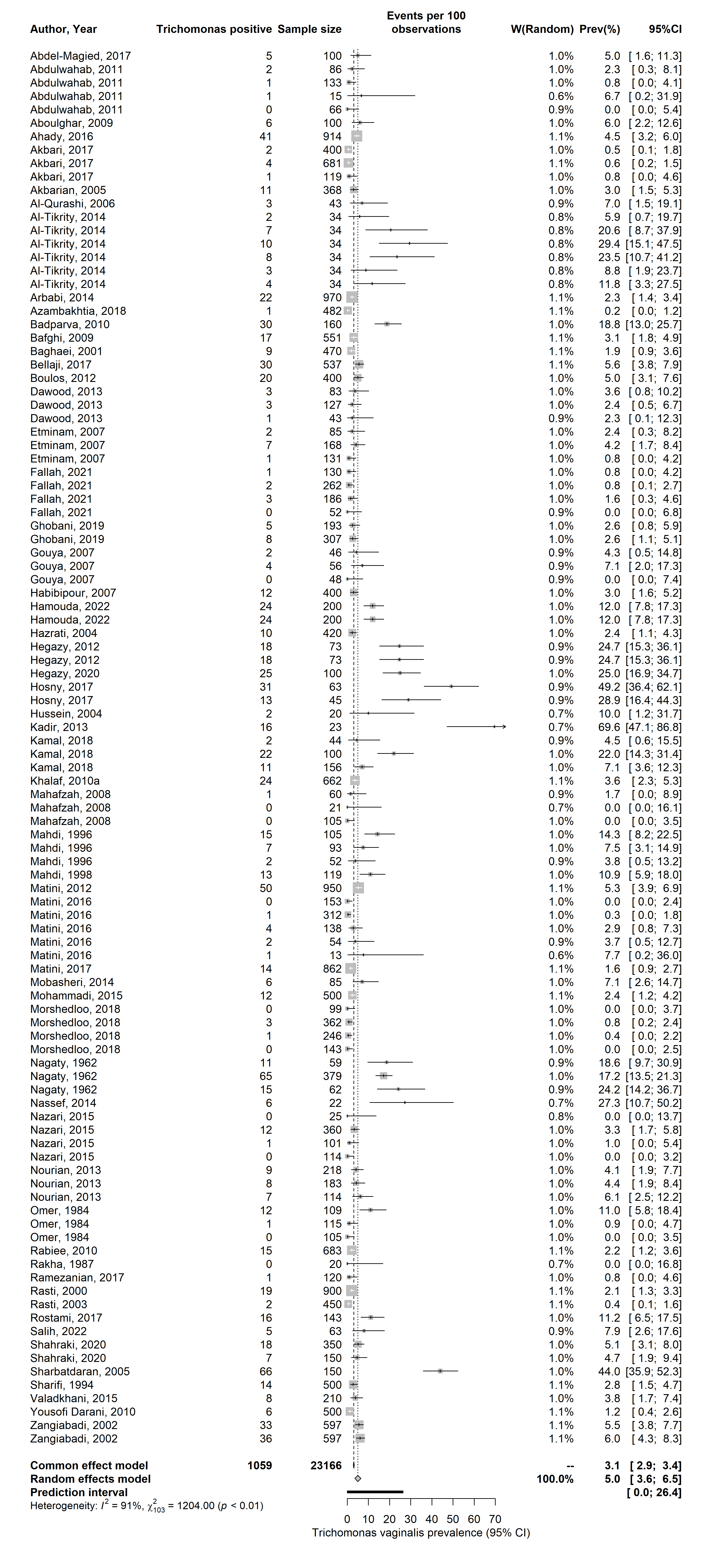
General populations tested using culture

Abbreviations: CI = Confidence interval.

1.
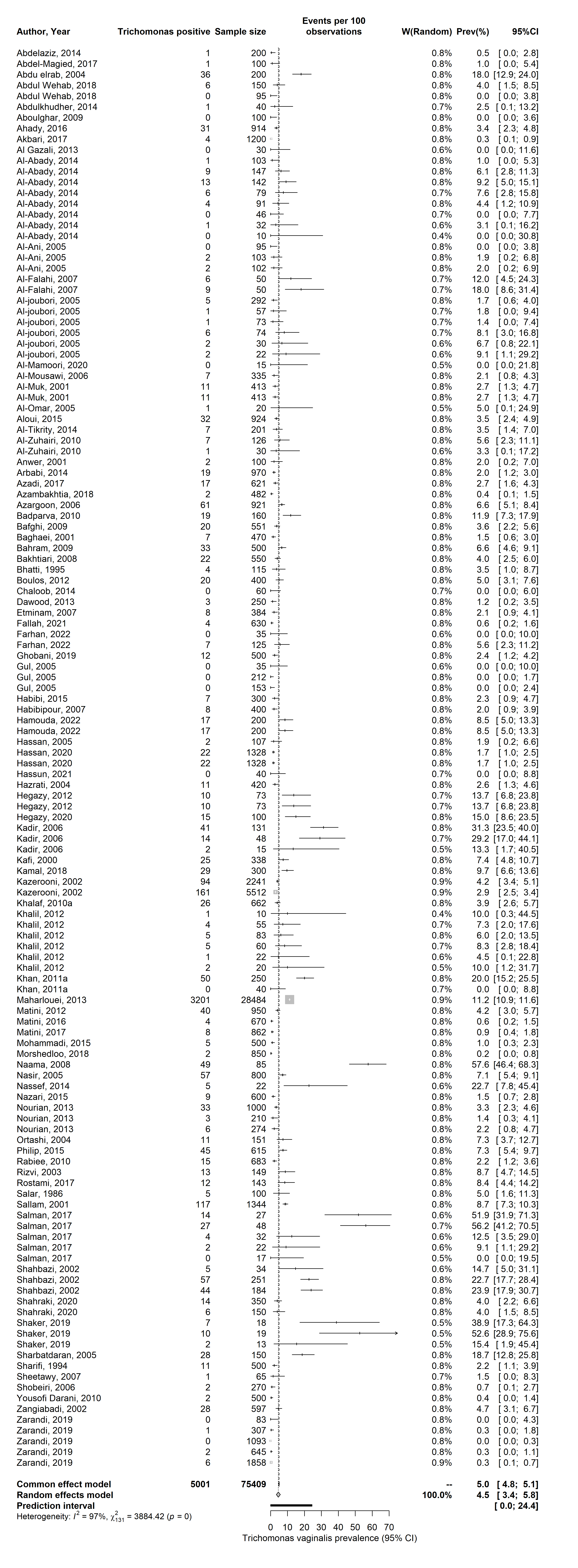
General populations tested using wet mount

Abbreviations: CI = Confidence interval.

The p-value of the Q-statistic is <0.01

1.
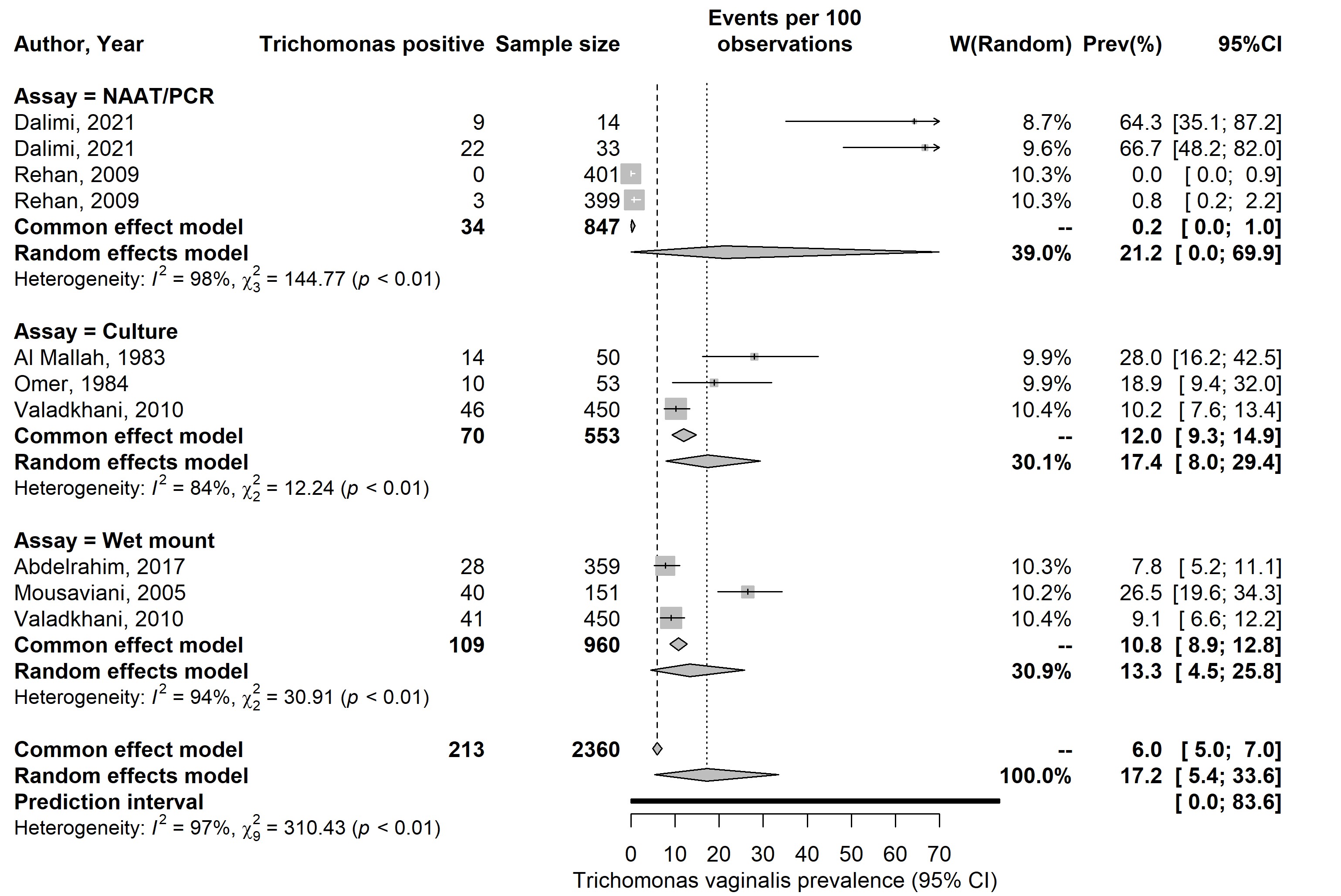
Intermediate risk populations

Abbreviations: CI = Confidence interval, NAAT = Nucleic acid amplification test, PCR = Polymerase chain reaction.

1.
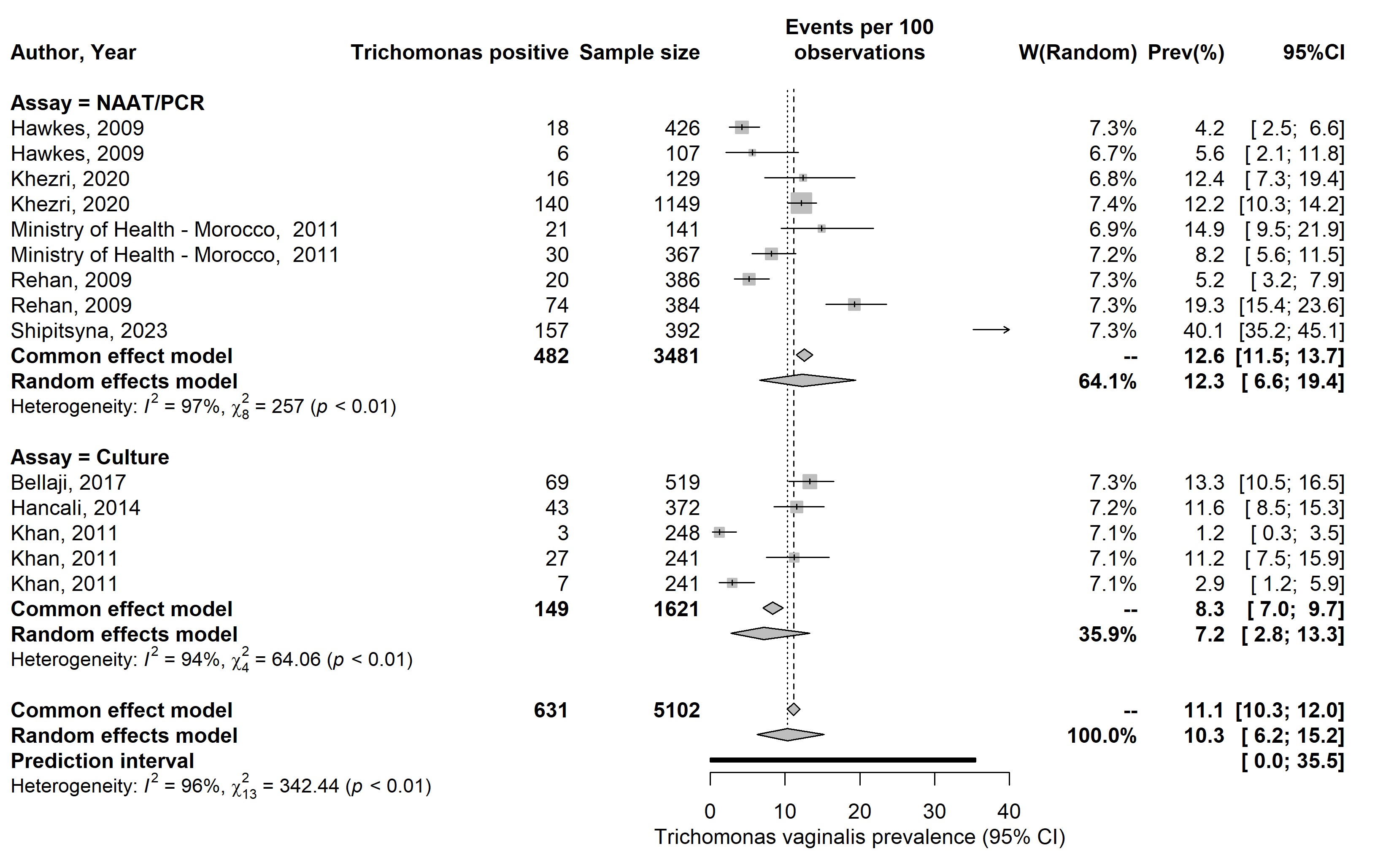
Female sex workers

Abbreviations: CI = Confidence interval, NAAT = Nucleic acid amplification test, PCR = Polymerase chain reaction.

1. Symptomatic women tested using NAAT/PCR


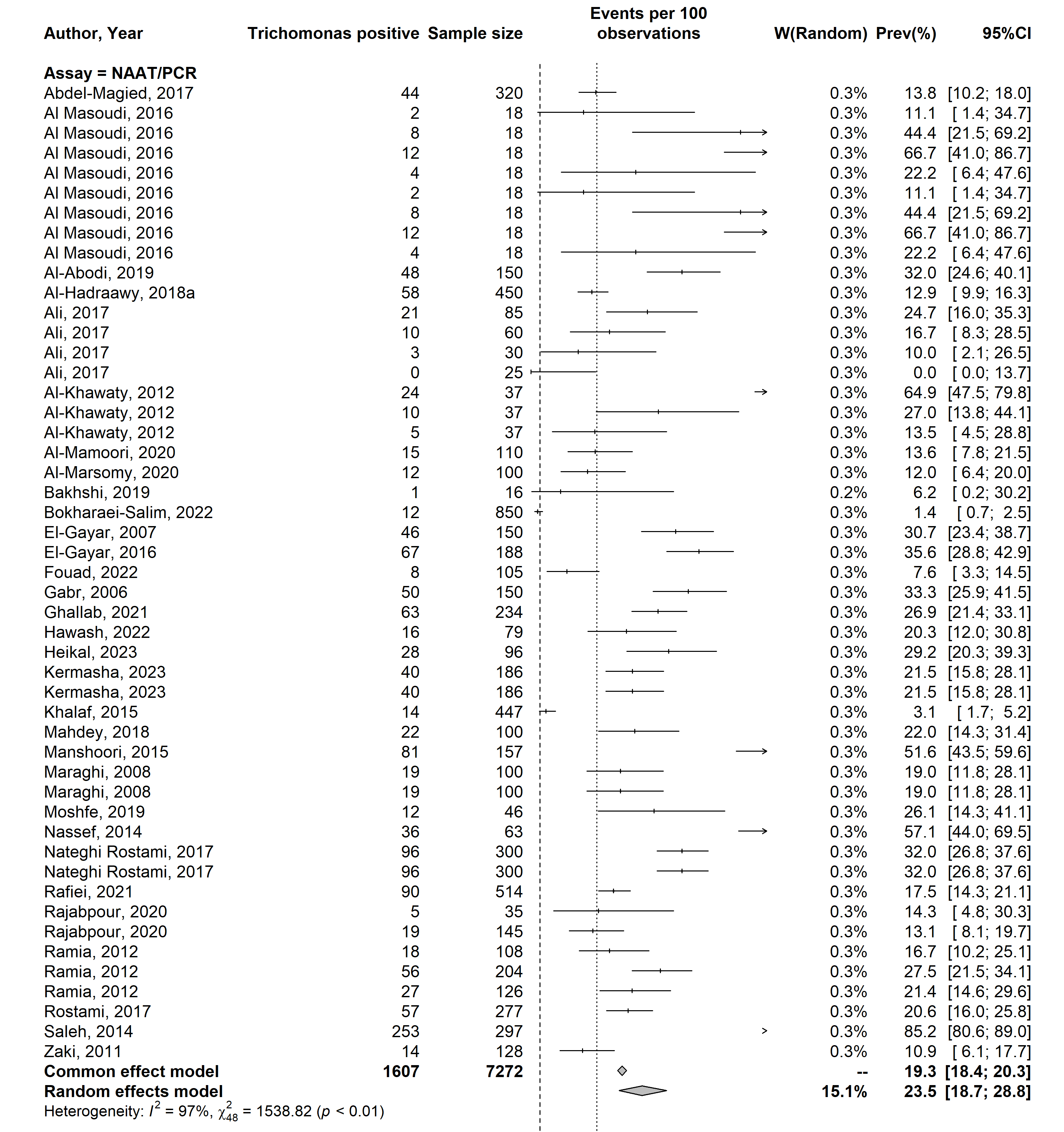
 Abbreviations: CI = Confidence interval, NAAT = Nucleic acid amplification test, PCR = Polymerase chain reaction.

1.
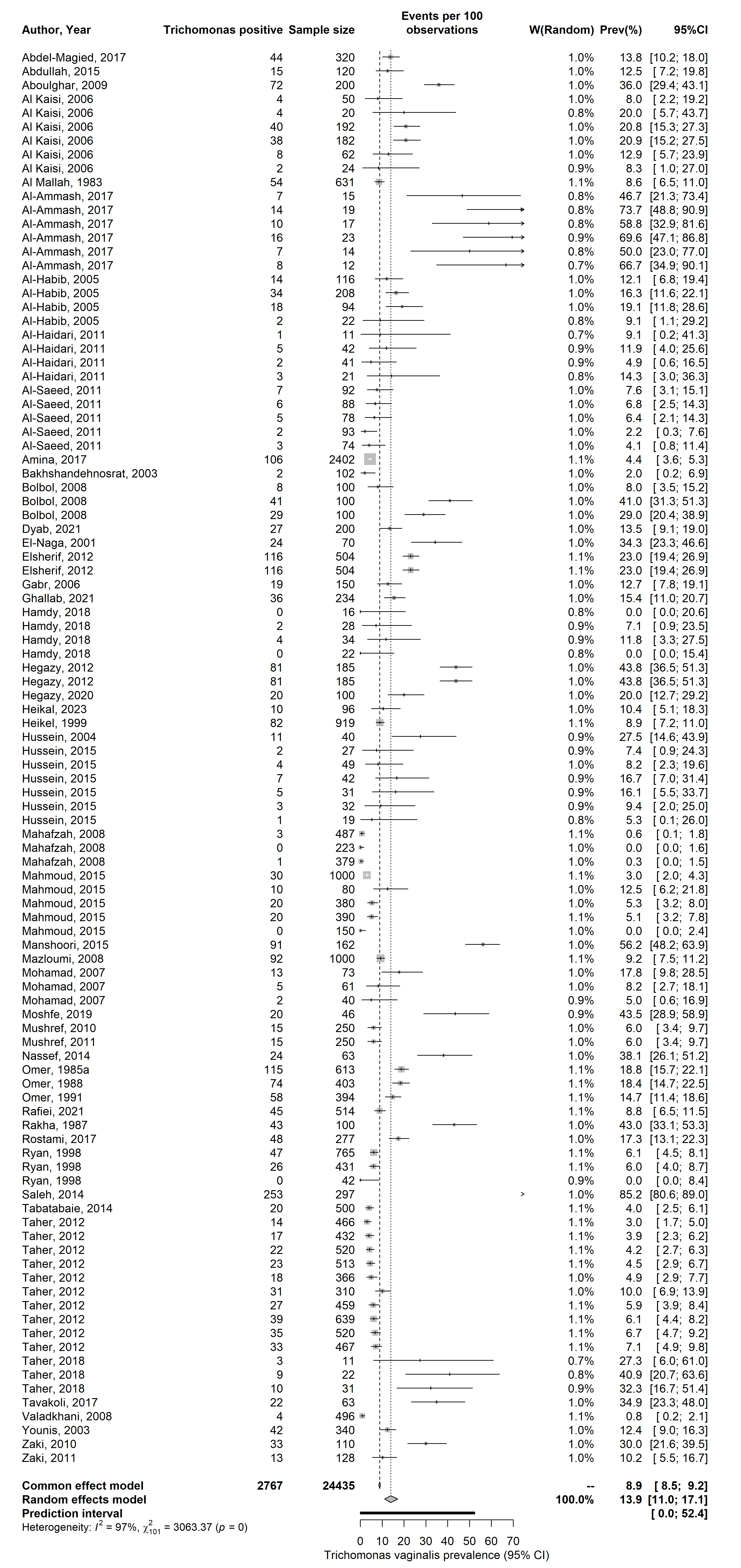
Symptomatic women tested using culture

Abbreviations: CI = Confidence interval.

The p-value of the Q-statistic is <0.01.

1.
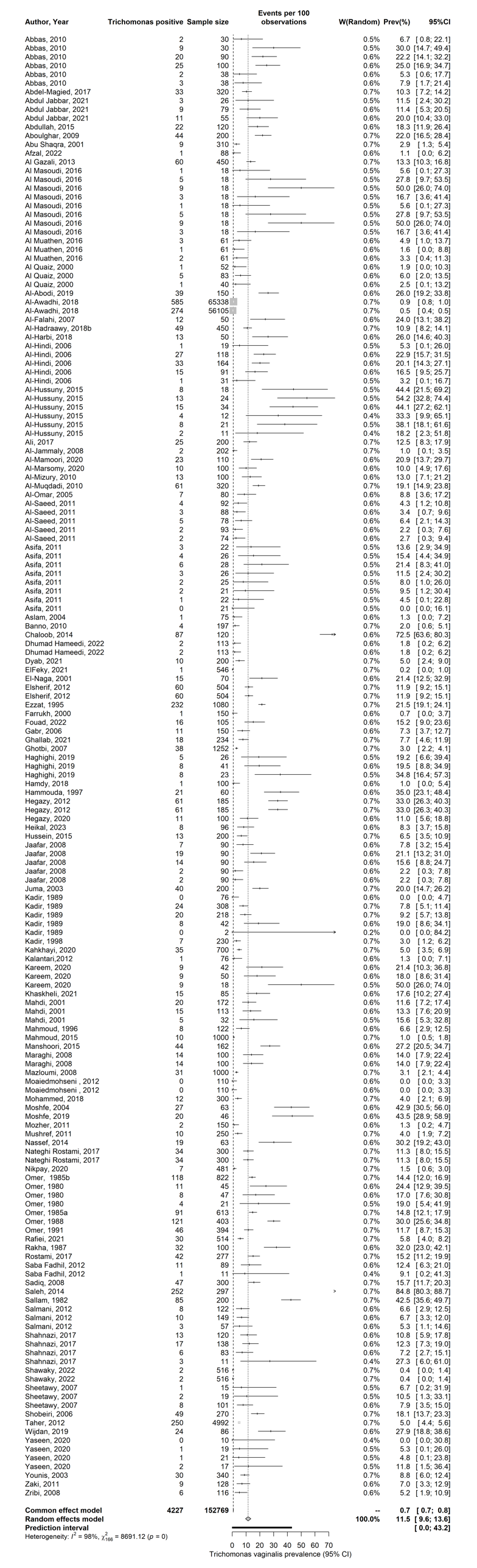
Symptomatic women tested using wet mount

Abbreviations: CI = Confidence interval.

The p-value of the Q-statistic is <0.01.

1. Symptomatic women tested using rapid test


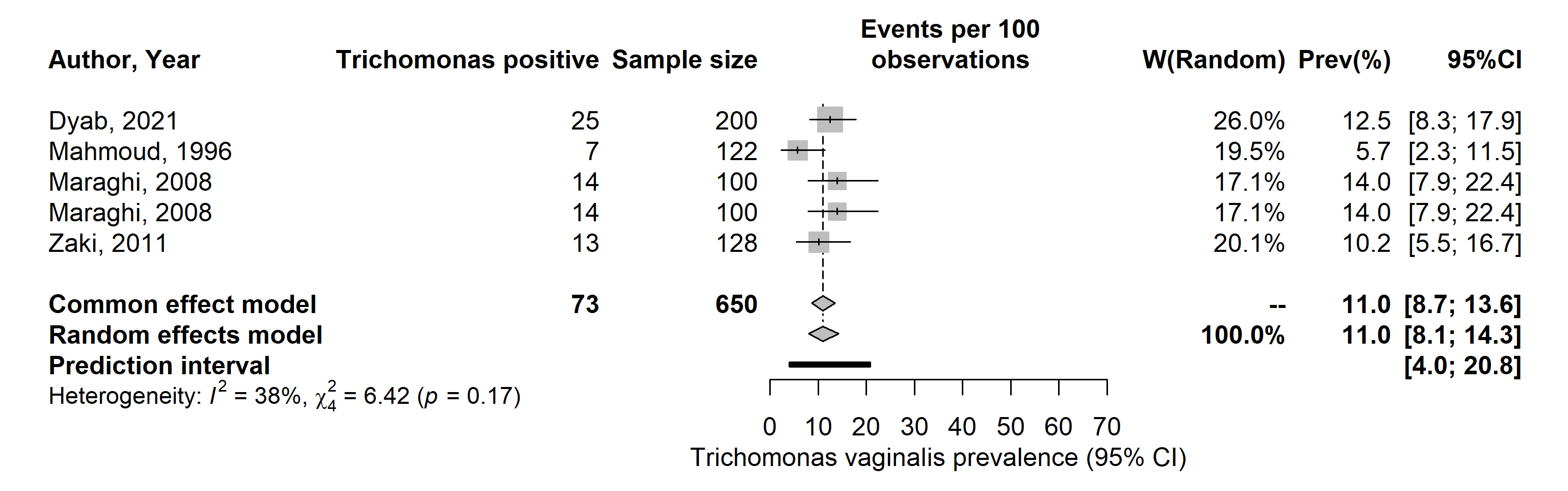
Abbreviations: CI = Confidence interval.

1. Symptomatic men


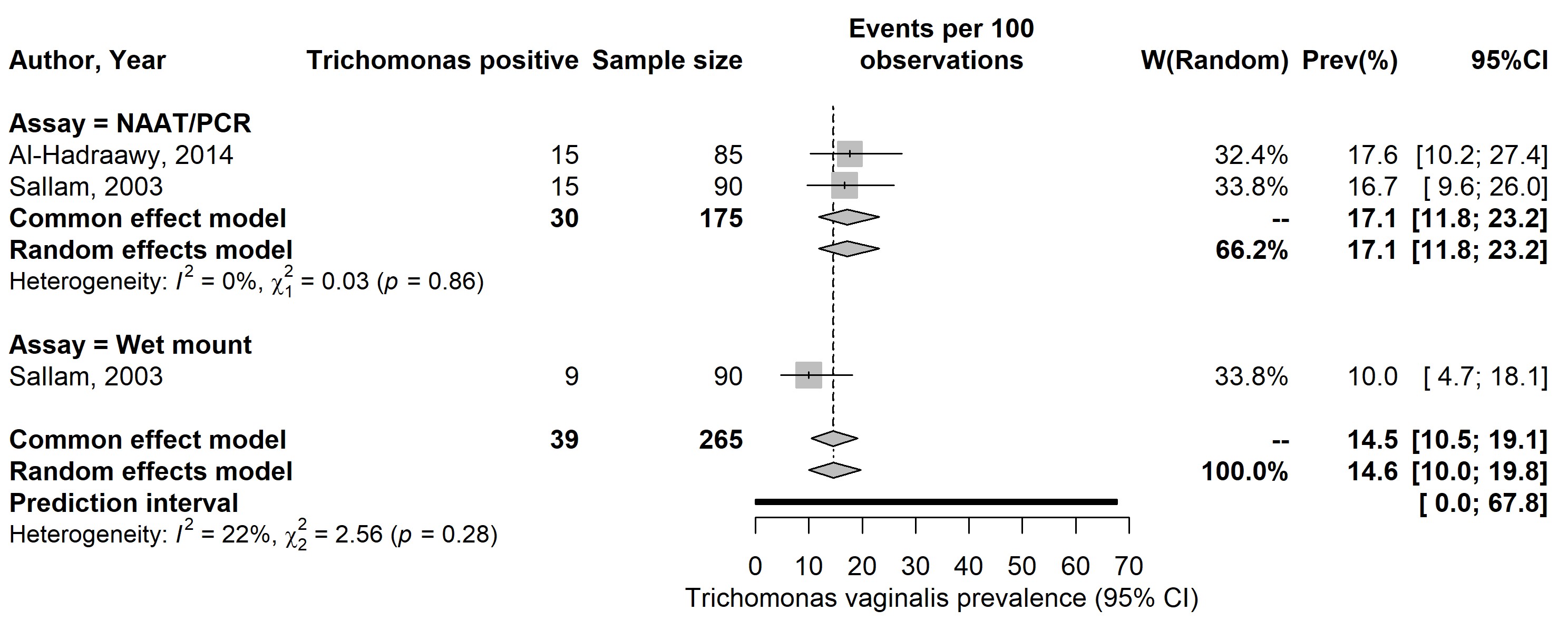


Abbreviations: CI = Confidence interval, NAAT = Nucleic acid amplification test, PCR = Polymerase chain reaction.

1.
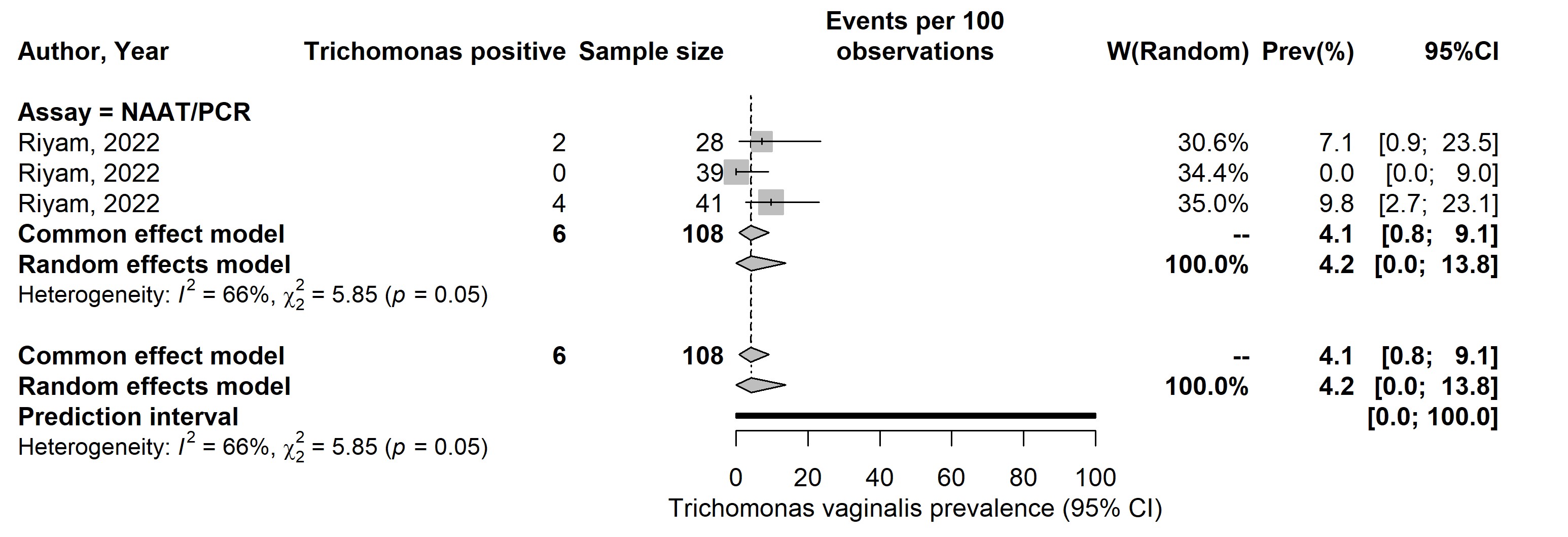
Symptomatic patients of mixed sexes

Abbreviations: CI = Confidence interval, NAAT = Nucleic acid amplification test, PCR = Polymerase chain reaction.

1. Infertility clinic attendees


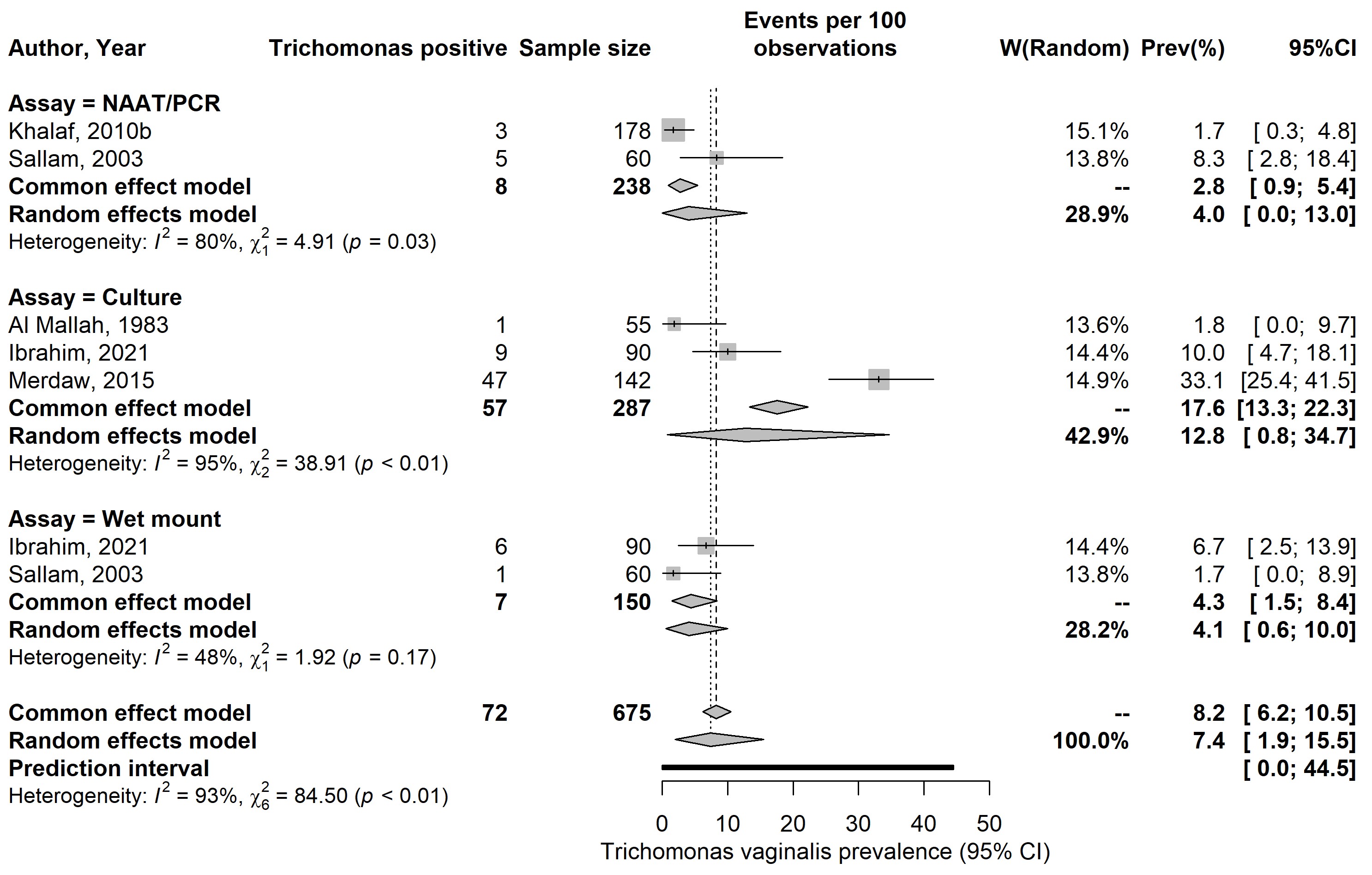


Abbreviations: CI = Confidence interval, NAAT = Nucleic acid amplification test, PCR = Polymerase chain reaction.

1.
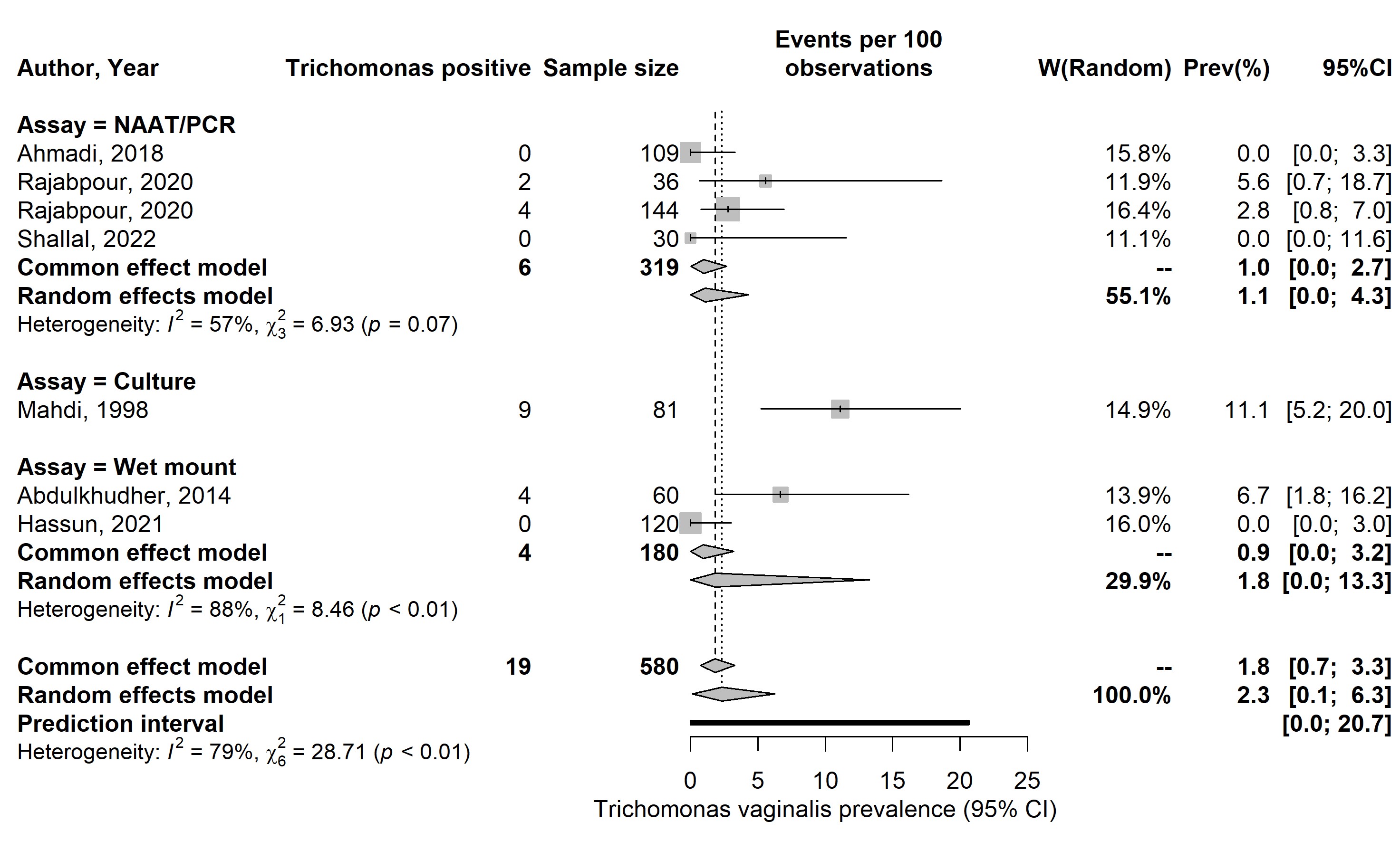
Women with miscarriages and ectopic pregnancies

Abbreviations: CI = Confidence interval, NAAT = Nucleic acid amplification test, PCR = Polymerase chain reaction.

1.
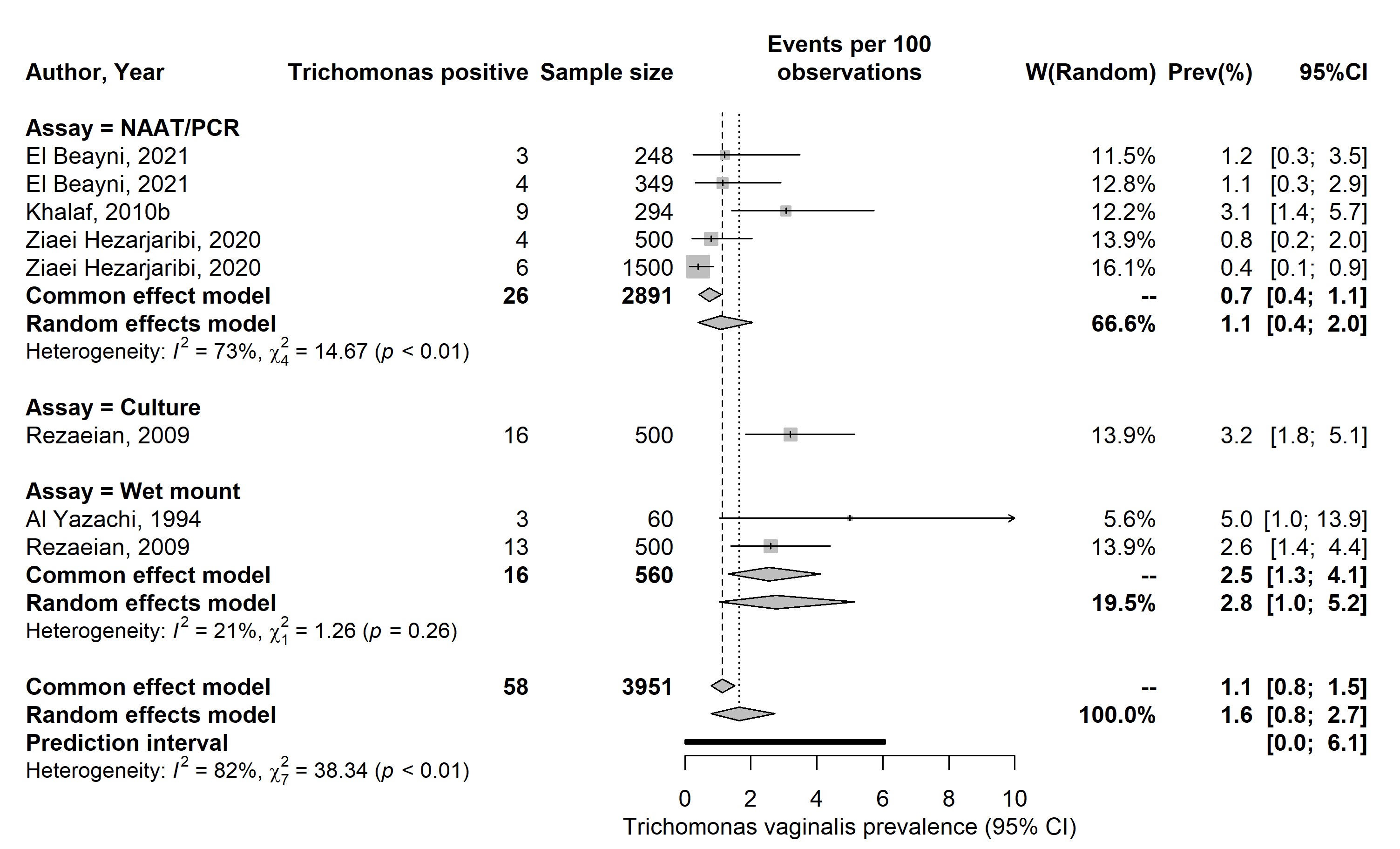
STI clinic attendees

Abbreviations: CI = Confidence interval, NAAT = Nucleic acid amplification test, PCR = Polymerase chain reaction, STI = Sexually transmitted infection.

1.
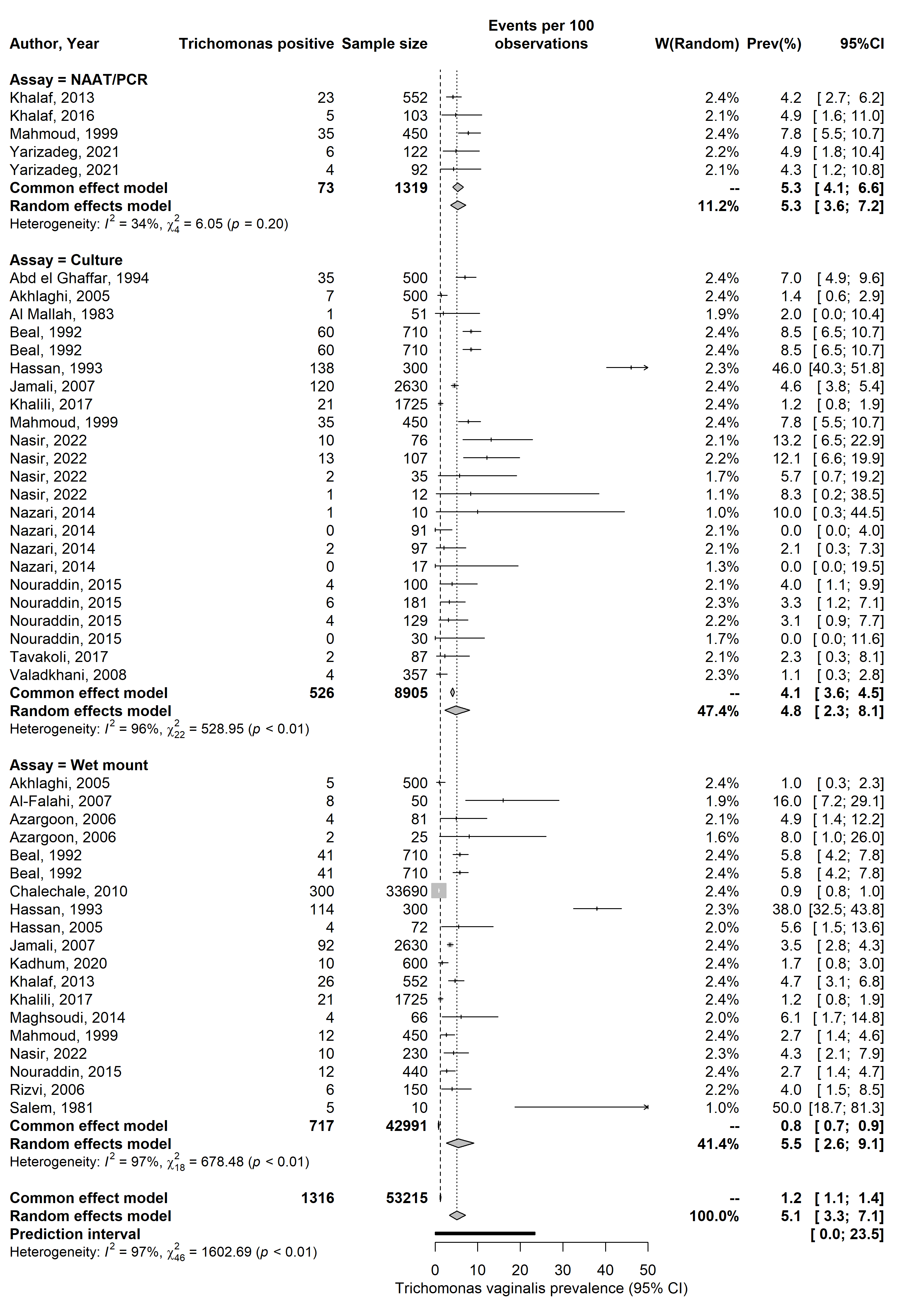
Other populations

Abbreviations: CI = Confidence interval, NAAT = Nucleic acid amplification test, PCR = Polymerase chain reaction.

# **Table S8.** Sensitivity analysis. Univariable and multivariable meta-regression analyses for *Trichomonas vaginalis* prevalence in the Middle East and North Africa, incorporating national income (in place of MENA subregion) and year of publication (in place of year of data collection) as variables. Prevalence measures conformed to the standard inclusion criteria for diagnostic methods.

|  | | | **Outcome measures** | **Sample size** | **Univariable analysis** | | | | **Multivariable analyses** | | | |
| --- | --- | --- | --- | --- | --- | --- | --- | --- | --- | --- | --- | --- |
|  |  |  | **Total n** | **Total N** | **PR (95% CI)** | **p-value** | **LR test p-value** | **Adjusted R^2^** | **Model 1^a^** | | **Model 2^b^** | |
|  |  |  |  |  |  |  |  |  | **APR (95% CI)** | **p-value** | **APR (95% CI)** | **p-value** |
| **Population characteristics** | **Population type** | General populations | 266 | 109,200 | 1.00 | - | <0.001 | 20.2 | 1.00 | - | 1.00 | - |
|  |  | Intermediate-risk populations | 10 | 2,360 | 3.29 (1.67-6.48) | 0.001 |  |  | 2.12 (1.18-3.81) | 0.012 | 2.02 (1.13-3.62) | 0.018 |
|  |  | Female sex workers | 14 | 5,102 | 1.87 (1.08-3.24) | 0.026 |  |  | 2.11 (1.18-3.80) | 0.012 | 2.27 (1.27-4.04) | 0.005 |
|  |  | Symptomatic women | 323 | 185,126 | 2.59 (2.16-3.11) | <0.001 |  |  | 2.18 (1.85-2.56) | <0.001 | 2.16 (1.83-2.54) | <0.001 |
|  |  | Symptomatic men | 3 | 265 | 3.00 (0.93-9.61) | 0.064 |  |  | 1.11 (0.41-3.05) | 0.83 | 1.18 (0.43-3.19) | 0.75 |
|  |  | Symptomatic patients (mixed sexes) | 3 | 108 | 1.71 (0.35-8.32) | 0.51 |  |  | 0.87 (0.21-3.57) | 0.85 | 0.89 (0.22-3.63) | 0.87 |
|  |  | Infertility clinic attendees | 7 | 675 | 1.33 (0.57-3.11) | 0.50 |  |  | 0.54 (0.26-1.15) | 0.11 | 0.54 (0.26-1.15) | 0.11 |
|  |  | Women with miscarriages or ectopic pregnancies | 7 | 580 | 1.18 (0.40-3.49) | 0.77 |  |  | 0.65 (0.25-1.71) | 0.39 | 0.66 (0.25-1.71) | 0.39 |
|  |  | STI clinic attendees | 8 | 3,951 | 0.35 (0.16-0.75) | 0.007 |  |  | 0.32 (0.16-0.62) | 0.001 | 0.31 (0.16-0.61) | 0.001 |
|  |  | HIV-positive individuals and individuals in HIV-discordant couples | 2 | 323 | 9.96 (2.50-39.58) | 0.001 |  |  | 7.99 (2.5-25.53) | <0.001 | 7.87 (2.48-24.95) | <0.001 |
|  |  | Other populations^c^ | 47 | 53,215 | 1.01 (0.72-1.43) | 0.94 |  |  | 1.06 (0.78-1.44) | 0.70 | 1.03 (0.76-1.39) | 0.86 |
|  | **Age group** | <20 years | 27 | 1,260 | 1.00 | - | <0.001 | 3.4 | 1.00 | - | 1.00 | - |
|  |  | 20-29 years | 50 | 5,745 | 1.17 (0.62-2.19) | 0.64 |  |  | 1.33 (0.80-2.22) | 0.28 | 1.39 (0.84-2.32) | 0.20 |
|  |  | 30-39 years | 47 | 5,009 | 1.11 (0.59-2.11) | 0.74 |  |  | 1.21 (0.72-2.04) | 0.46 | 1.26 (0.75-2.11) | 0.38 |
|  |  | 40-49 years | 29 | 2,129 | 0.74 (0.36-1.52) | 0.41 |  |  | 0.77 (0.42-1.40) | 0.39 | 0.80 (0.44-1.45) | 0.46 |
|  |  | ≥50 years | 15 | 2,494 | 0.28 (0.10-0.77) | 0.014 |  |  | 0.43 (0.19-1.02) | 0.054 | 0.46 (0.20-1.08) | 0.074 |
|  |  | Mixed ages | 522 | 344,268 | 0.66 (0.38-1.15) | 0.15 |  |  | 0.74 (0.47-1.17) | 0.20 | 0.77 (0.49-1.22) | 0.27 |
|  | **Sex** | Women | 669 | 356,664 | 1.00 | - | 0.74 | 0.0 | - | - | - | - |
|  |  | Men | 16 | 4,040 | 0.82 (0.45-1.50) | 0.51 |  |  | - | - | - | - |
|  |  | Mixed sexes | 5 | 201 | 0.75 (0.21-2.68) | 0.66 |  |  | - | - | - | - |
|  | **MENA subregion^d^** | Fertile crescent | 413 | 65,583 | 1.00 | - | <0.001^e^ | 15.0 | - | - | - | - |
|  |  | Horn of Africa | 22 | 6,276 | 1.53 (0.96-2.43) | 0.075 |  |  | - | - | - | - |
|  |  | Maghreb | 13 | 7,927 | 0.78 (0.43-1.41) | 0.41 |  |  | - | - | - | - |
|  |  | Gulf | 10 | 124,975 | 0.21 (0.10-0.42) | <0.001 |  |  | - | - | - | - |
|  |  | Iran | 205 | 149,568 | 0.44 (0.36-0.53) | <0.001 |  |  | - | - | - | - |
|  |  | Pakistan | 27 | 6,576 | 0.44 (0.27-0.71) | 0.001 |  |  | - | - | - | - |
|  | **National income** | LIC and LMIC | 187 | 47,363 | 1.00 | - | <0.001 | 5.5 | 1.00 | - | 1.00 | - |
|  |  | UMIC | 493 | 188,567 | 0.63 (0.52-0.77) | <0.001 |  |  | 0.71 (0.60-0.85) | <0.001 | 0.74 (0.62-0.87) | <0.001 |
|  |  | HIC | 10 | 124,975 | 0.20 (0.09-0.42) | <0.001 |  |  | 0.21 (0.12-0.39) | <0.001 | 0.22 (0.12-0.41) | <0.001 |
| **Study methodology characteristics** | **Assay type** | NAAT/PCR | 114 | 27,548 | 1.00 | - | 0.023 | 1.2 | 1.00 | - | 1.00 | - |
|  |  | Culture | 243 | 59,598 | 0.75 (0.57-0.96) | 0.032 |  |  | 0.77 (0.62-0.97) | 0.024 | 0.75 (0.6-0.94) | 0.012 |
|  |  | Wet mount | 328 | 273,109 | 0.67 (0.52-0.87) | 0.003 |  |  | 0.63 (0.51-0.79) | <0.001 | 0.62 (0.5-0.77) | <0.001 |
|  |  | Rapid test | 5 | 650 | 1.02 (0.37-2.82) | 0.96 |  |  | 0.44 (0.20-0.96) | 0.040 | 0.43 (0.2-0.95) | 0.037 |
|  | **Sample size** | <200 | 272 | 21,288 | 1.00 | - | <0.001 | 19.3 | 1.00 | - | 1.00 | - |
|  |  | ≥200 | 418 | 339,617 | 0.39 (0.33-0.46) | <0.001 |  |  | 0.45 (0.38-0.53) | <0.001 | 0.45 (0.38-0.52) | <0.001 |
|  | **Sampling method** | Probability based | 50 | 18,061 | 1.00 | - | 0.15 | 0.2 | 1.00 | - | 1.00 | - |
|  |  | Non-probability based | 640 | 342,844 | 1.30 (0.91-1.85) | 0.15 |  |  | 0.90 (0.66-1.23) | 0.50 | 0.90 (0.66-1.23) | 0.51 |
|  | **Response rate** | ≥80% | 25 | 10,042 | 1.00 | - | 0.037 | 0.9 | 1.00 | - | 1.00 | - |
|  |  | <80% | 6 | 505 | 4.05 (1.30-12.54) | 0.015 |  |  | 6.98 (2.72-17.91) | <0.001 | 7.32 (2.87-18.64) | <0.001 |
|  |  | Unclear | 660 | 360,905 | 1.61 (0.97-2.69) | 0.066 |  |  | 2.17 (1.35-3.48) | 0.001 | 2.30 (1.43-3.70) | 0.001 |
| **Temporal trend** | **Year of data collection category** | <2000 | 102 | 35,646 | 1.00 | - | 0.088^f^ | 0.5 | - | - | - | - |
|  |  | 2000-2009 | 253 | 244,359 | 0.75 (0.57-0.99) | 0.042 |  |  | - | - | - | - |
|  |  | ≥2010 | 335 | 80,900 | 0.89 (0.68-1.15) | 0.37 |  |  | - | - | - | - |
|  | **Year of data collection** | | 690 | 360,905 | 0.99 (0.98-1.00) | 0.24 | 0.24^f^ | 0.0 | - | - | - | - |
|  | **Year of publication category** | <2005 | 110 | 36,657 | 1.00 | - | 0.84 | 0.0 | 1.00 | - | - | - |
|  |  | 2005-2014 | 291 | 133,322 | 0.93 (0.71-1.21) | 0.58 |  |  | 0.86 (0.69-1.07) | 0.19 | - | - |
|  |  | ≥2015 | 289 | 190,926 | 0.93 (0.72-1.22) | 0.61 |  |  | 0.75 (0.60-0.94) | 0.013 | - | - |
|  | **Year of publication** | | 690 | 360,905 | 0.99 (0.98-1.00) | 0.055 | 0.055 | 0.4 | - | - | 0.99 (0.98-0.99) | <0.001 |

Abbreviations: APR = Adjusted prevalence ratio, CI = Confidence interval, HIC = High-income country, HIV = Human immunodeficiency virus, MENA = Middle East and North Africa, NAAT = Nucleic acid amplification test, LIC = Low-income country, LMIC = Low-middle-income country, LR test = Likelihood ratio test, PCR = Polymerase chain reaction, PR = Prevalence ratio, STI = Sexually transmitted infection, UMIC = Upper-middle-income country.

The PR represents the exponentiated beta coefficient calculated by the meta-regression model.

^a^ Adjusted R^2^ in the final multivariable model 1 = 44.3%. Model 1 includes population type, age group, national income, assay type, sample size, sampling method, response rate, and year of publication as a categorical variable. Other variables were not included either because their p-values in the univariable model were greater than 0.2 or due to collinearity with another variable included in the model.

^b^ Adjusted R^2^ in the final multivariable model 2 = 45.1%. Model 2 includes population type, age group, national income, assay type, sample size, sampling method, response rate, and year of publication as a continuous linear term. Other variables were not included either because their p-values in the univariable model were greater than 0.2 or due to collinearity with another variable included in the model.

^c^ Other populations include populations with an undetermined risk of acquiring *Trichomonas vaginalis* infection such as women with premature labor, cancer patients, patients suffering from diabetes, and mixed at-risk populations, among others.

^d^ Countries included in each MENA subregion are as follows: Fertile Crescent (Egypt, Iraq, Jordan, Lebanon, Palestine, Syria); Horn of Africa (Djibouti, Somalia, Sudan, Yemen); Maghreb (Algeria, Libya, Morocco, Tunisia); and Gulf (Bahrain, Kuwait, Oman, Qatar, Saudi Arabia, United Arab Emirates).

^e^ MENA subregion was not included in the multivariable model due to collinearity with national income variable.

^f^ Year of data collection was not included in the multivariable model due to collinearity with year of publication variable.

# **References**

1. Moher D, Liberati A, Tetzlaff J, Altman DG, Group P. Preferred reporting items for systematic reviews and meta-analyses: the PRISMA statement. *Journal of Clinical Epidemiology* 2009; **62**(10): 1006-12.

2. Page MJ, McKenzie JE, Bossuyt PM, et al. The PRISMA 2020 statement: an updated guideline for reporting systematic reviews. *BMJ* 2021; **372**: n71.

3. Hoy D, Brooks P, Woolf A, et al. Assessing risk of bias in prevalence studies: modification of an existing tool and evidence of interrater agreement. *Journal of clinical epidemiology* 2012; **65**(9): 934-9.

4. Munn Z, Moola S, Lisy K, Riitano D, Tufanaru C. Methodological guidance for systematic reviews of observational epidemiological studies reporting prevalence and cumulative incidence data. *JBI Evidence Implementation* 2015; **13**(3): 147-53.

5. World Bank. World Bank Country and Lending Groups. <https://datahelpdesk.worldbank.org/knowledgebase/articles/906519-world-bank-country-and-lending-groups>. (accessed in June, 2017). 2017.

6. IntHout J, Ioannidis JPA, Borm GF. The Hartung-Knapp-Sidik-Jonkman method for random effects meta-analysis is straightforward and considerably outperforms the standard DerSimonian-Laird method. *BMC Medical Research Methodology* 2014; **14**(1): 25.

7. Röver C, Knapp G, Friede T. Hartung-Knapp-Sidik-Jonkman approach and its modification for random-effects meta-analysis with few studies. *BMC Medical Research Methodology* 2015; **15**(1): 99.

8. Wiksten A, Rucker G, Schwarzer G. Hartung-Knapp method is not always conservative compared with fixed-effect meta-analysis. *Stat Med* 2016; **35**(15): 2503-15.
